# Transferring Substituents from Alkynes to Furans and Pyrroles through Heteronorbornadienes as Intermediates: Synthesis of β-Substituted Pyrroles/Furans

**DOI:** 10.1021/acs.joc.3c01145

**Published:** 2023-08-24

**Authors:** Javier García-Domínguez, Marina Carranza, Edijs Jansons, Ana T. Carmona, Inmaculada Robina, Antonio J. Moreno-Vargas

**Affiliations:** Departamento de Química Orgánica, Facultad de Química, Universidad de Sevilla, 41012 Sevilla, Spain

## Abstract

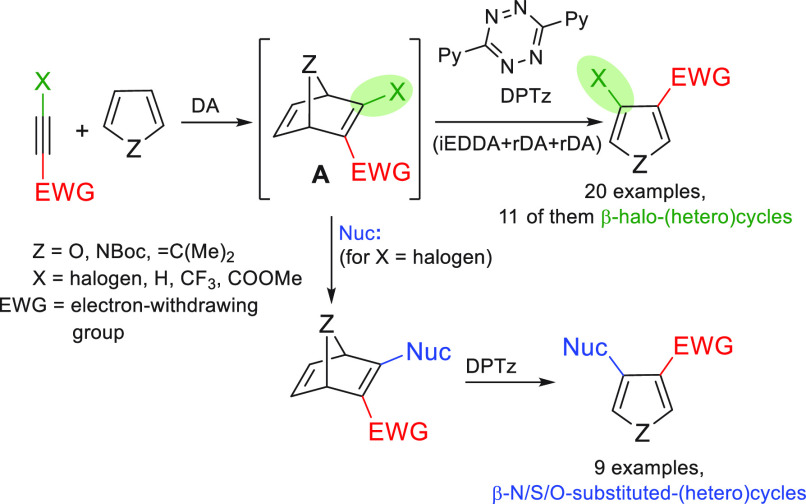

The use of 7-oxa/azanorbornadienes as synthetic intermediates for the preparation of 3/4-substituted (β-substituted) furans/pyrroles is presented. The method lies in the inverse electron demand Diels–Alder (iEDDA) cycloaddition between a substituted heteronorbornadiene and an electron-poor tetrazine followed by spontaneous fragmentation of the resulting cycloadduct *via* two retro-Diels–Alder (rDA) reactions affording a β-substituted furan/pyrrole. The scope of this tandem iEDDA/rDA/rDA reaction was explored in the preparation of 29 heterocycles. A one-pot procedure starting directly from the alkyne precursors of the heteronorbornadiene intermediates is also described.

Furan and pyrrole are two of the most representative five-membered heterocycles. Both structural motifs are ubiquitously present in natural products or pharmaceuticals with high biological activity^1^ and compounds of significance for material sciences.^2^ Both heterocycles can incorporate up to four substituents (five in the case of *N*-substituted pyrroles), however, the most challenging substitution pattern is 3,4-disubstitution (β-substitution).^3^ In fact, the regioselective synthesis of β-substituted furans/pyrroles continues to attract the attention of researchers in the last years.^4^ Classic syntheses of furan and pyrrole derivatives from acyclic carbonyl precursors, such as Paal-Knorr, Feist-Benary, and Hantzsch procedures, among others, generally work best for fully substituted heterocycles or for 2,5-disubstituted systems.^3a,5^ Procedures based on the electrophilic aromatic substitution of preformed heterocycles are not a good alternative for the 3,4-substituted derivatives as C-2 is usually much more reactive toward electrophiles.^6^ Moreover, lithiation of furans or *N*-protected pyrroles also occurs preferentially at C-2 and C-5.^7^ We have recently reported the tandem regioselective 1,3-dipolar cycloaddition and subsequent retro-Diels–Alder reaction between organic azides and 7-heteronorbornadienes (Scheme 1a).^4b^ This procedure allowed us to carry out efficiently the preparation of a small library of 3,4-disubstituted heterocycles. The regioselectivity of the 1,3-cycloaddition was controlled by tuning the electronic density of the electron-poor double bond using the adequate substituents (R^1^ and R^2^) on the heteronorbornadiene.

**Scheme 1:**
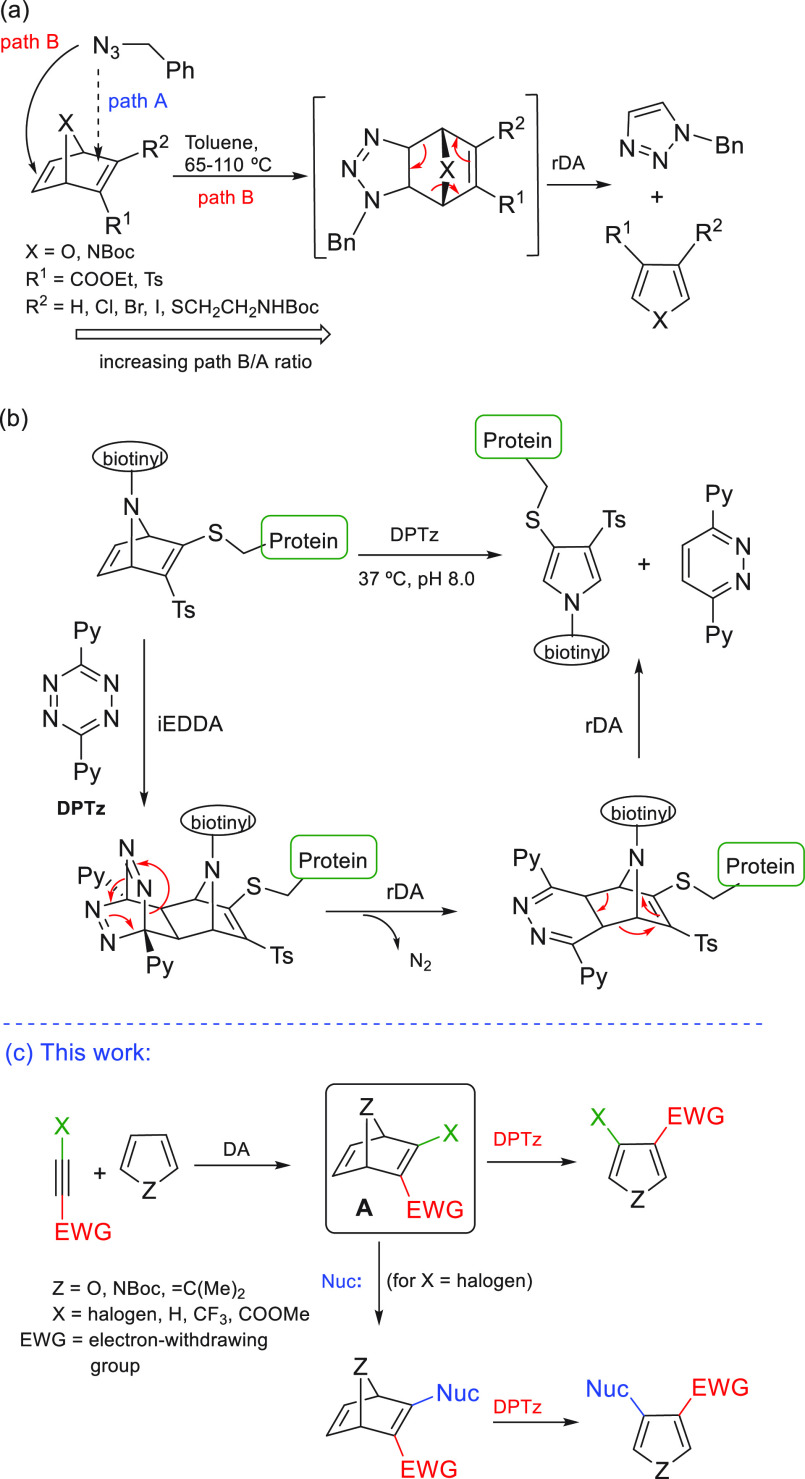
Our Previous Work on the Synthesis of β-Substituted Pyrroles/Furans through Heteronorbornadienic Intermediates and the New Proposal

As part of our program for the use of heteronorbornadienic systems in the selective modification of proteins, we have recently found that the treatment of an azanorbornadiene-modified protein with the electron-deficient 3,6-di(2-pyridyl)-*s*-tetrazine (DPTz) afforded the β-substituted pyrrole-modified protein (Scheme 1b) under mild conditions and with total regioselectivity.^8^ This transformation took place through a three-step cascade reaction: (i) ligation between a tetrazine and the available electron-rich alkene of the azanorbornadiene system through an inverse electron demand Diels–Alder (iEDDA) cycloaddition; (ii) first retro-Diels–Alder reaction (rDA) with extrusion of N_2_; (iii) second rDA with extrusion of 3,6-dipyridyl pyridazine. A similar reaction had been previously used by Warrener and co-workers for the preparation of isoindoles and isobenzofurans by reaction of heterobenzonorbornadienes with DPTz.^9^ The procedure was also exploited for the preparation of substituted cyclopentadienes from norbornadiene derivatives, however, the inherent instability of the resulting cyclopentadienes complicated their characterization.^10^

With these results in hand, in this work we envisaged that heteronorbornadienes of type **A** could be versatile substrates for the preparation of β-substituted(halo) pyrroles/furans (Scheme 1c). Bicyclic systems **A** contain an electron-poor and an electron-rich double bond, being the latter the only one able to react in the initial iEDDA that guarantees the regioselectivity of the process. Heteronorbornadienes of type **A** can be easily prepared from the adequate electron-deficient alkyne and furan/pyrrole via Diels–Alder (DA) cycloaddition. All together this strategy implies the transfer of the substituents of the activated alkyne (X and EWG) to the furan or pyrrole skeleton. When X = halogen, valued β-halopyrroles/furans are the resulting products. These compounds have special interest as direct precursors of metalated heterocycles that are excellent synthetic intermediates in C–C and C–S bond forming reactions.^7,11^ Moreover, halo-pyrroles/furans are also employed in C–N bond formation as they are substrates for the palladium-catalyzed amination.^12^ Additionally, the functionalization of halo-heteronorbornadienes of type **A** will be also explored in order to expand the scope of this methodology.

Most (hetero)norbornadienes were prepared by DA cycloaddition between furan/*N*-Boc-pyrrole and activated alkynes (commercial or synthetically achievable) as it was reported in previous works.^13^ Bicyclic bromovinyl phosphonates **9j**–**9m** were prepared here for the first time. With this (hetero)norbornadienic substrates in hand, we decided to explore the tandem iEDDA/rDA/rDA by reaction with DPTz as it is shown in Scheme 2 (Procedure A). Additionally, in order to simplify the experimental procedure, two additional approaches were assayed in selected cases: (i) reaction of the alkyne with a mixture of the cyclic diene and DPTz (Procedure B, only used when the heteronorbornadienes are efficiently obtained under mild conditions); (ii) synthesis of the heteronorbornadienic intermediate and subsequent addition of the tetrazine in one-pot (Procedure C).

**Scheme 2:**
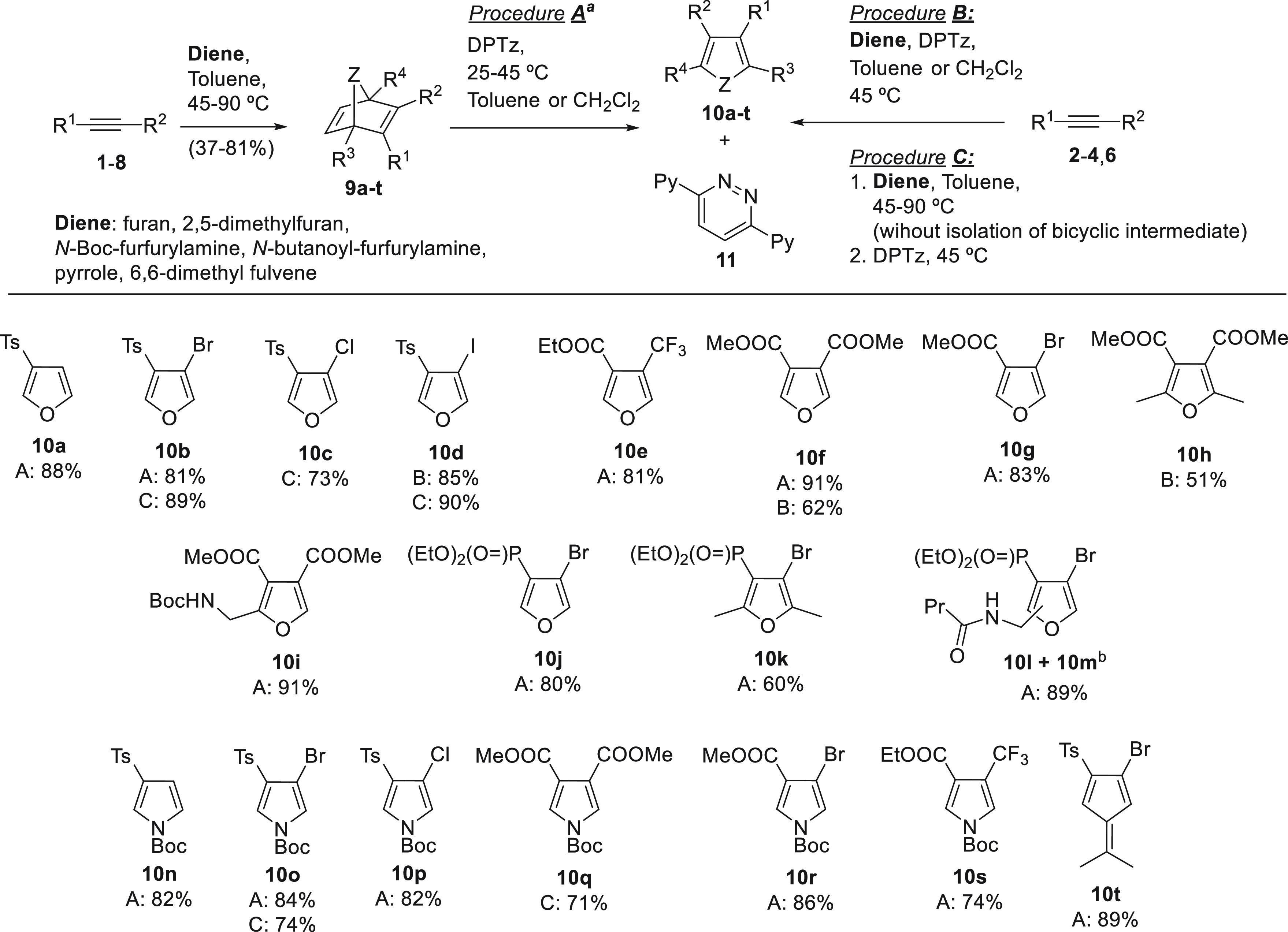
Synthesis of β-Substituted Furans/Pyrroles/Fulvenes via (Hetero)norbornadiene Intermediates^,^ Yields corresponding to Procedure A are referred exclusively to the iEDDA/rDA/rDA step from pure heteronorbornadienes **9a**-**t**. Regioisomers **10l** and **10m**, obtained from a regioisomeric mixture of oxanorbornadienes **9l** and **9m**, could be separated.

In general, all the five-membered heterocycles (and carbacycle **10t**) were obtained in excellent yields under mild conditions (25–45 °C) following the procedure A. Furan **10d**, **10f**, and **10h** were obtained in moderate-to-good yields using the procedure B. The procedure B could not be used in the synthesis of pyrrole derivatives because the Diels–Alder that leads to the formation of the azanorbornadienic intermediates requires higher temperature (∼90 °C) than for the oxa-analogues and, unfortunately, DPTz was unstable at this temperature. The procedure C was successfully assayed for the synthesis of pyrrole derivative **10q** and oxa-analogues **10b**−**10d**. It is important to highlight that the iEDDA reaction was totally regioselective, in contrast to the 1,3-dipolar cycloaddition of our previous strategy (Scheme 1a) where the regioselectivity was highly dependent on the substituents of the heteronorbornadiene. In addition, as the temperature required for the new experiments is lower than the one required for our previous strategy,^4b^ no Boc deprotection was observed for *N*-Boc pyrrole derivatives.^14^

Then we decided to exploit the presence of the halogen into the heteronorbornadienic system in order to increase the scope of the methodology. Thus, the bromo-azabicyclic system **9o**, that we had previously used for the modification of proteins,^8^ was first selected to explore the thio-functionalization of the C2 position. As expected, this compound reacted efficiently with *N*-Boc cysteamine under mild conditions (Scheme 3); a similar behavior was observed for the reaction of the oxa-analogues **9b,j,k** and carba-analogue **9t** with *N*-acetylcysteamine. The resulting thiovinyl sulfones/phosphonates **12**-**16** were isolated and subsequently made to react with DPTz under mild conditions affording β-thio(hetero)cycles **17**-**21** in excellent yields. Next, we explored the incorporation of *N*- and *O*-based nucleophiles (Scheme 4). Thus, bicyclic systems **9b** and **9o** reacted with diethylamine in the presence of triethylamine in a fast and clean reaction. The resulting enamines were not isolated and reacted with DPTz in a one-pot procedure furnishing the corresponding β-aminoheterocycles **22** and **23** in 46 and 51% yield (two steps), respectively. Following a similar strategy, the pyridinum salt **24** was also prepared from oxanorbornadiene **9b**, as the bromovinyl sulfone functionality is electrophilic enough to accept the attack of pyridine. Finally, the introduction of oxygenated nucleophiles was first attempted with oxabicyclic bromovinyl sulfone **9b**, using MeOH in the presence of DBU. However, besides the nucleophilic substitution, the conjugate addition also took place, affording undesired ketal **25** in excellent yield. Replacement of the tosyl electron-withdrawing group by a diethyl phosphonate decreased the reactivity of the oxanorbornadiene system and enol ether derivative **26** was obtained under the same reaction conditions. Treatment of **26** wih DPTz afforded the corresponding β-methoxyfuran **27**.

**Scheme 3:**
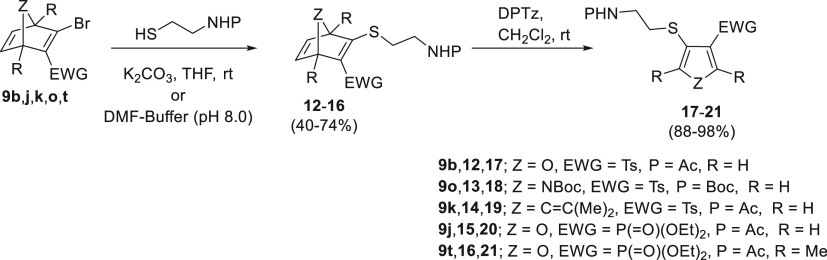
Thio-functionalization of (Hetero)norbornadienes **9b**,**j**,**k**,**o**,**t** at C2 Position: Synthesis of β-Thio-heterocycles

**Scheme 4:**
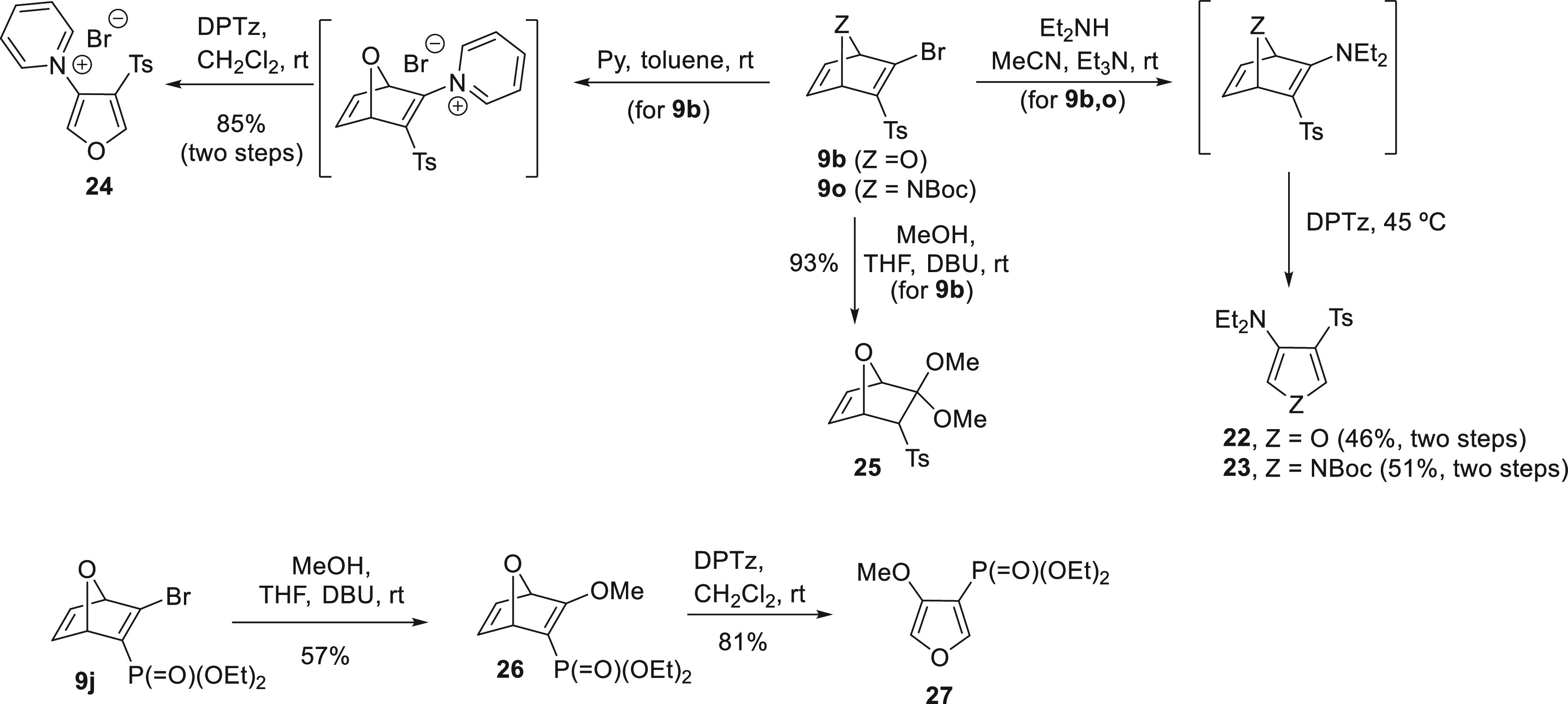
*N*/*O*-Functionalization of (Hetero)norbornadienes **9b**,**j**,**o** at C2 Position: Synthesis of β-Amino/pyridinium/alkoxy-heterocycles

## Conclusions

In summary, we have demonstrated that differently functionalized heteronorbornadienes react with an electron-poor tetrazine through a tandem iEDDA/rDA/rDA sequence affording β-substituted furans/pyrroles in excellent yields. As the heteronorbornadienes are prepared *via* DA reaction between electron-poor alkynes and cyclic dienes, the resulting bicyclic adducts always contain two double bonds with very different electronic density, that guarantees the total regioselectivity in the further iEDDA with the electron-poor tetrazine. The halovinyl sulfone/ester/phosphonate embedded in the bicyclic system allows the easy functionalization of the structure, expanding the scope of the methodology. When the initial DA reaction that leads to the heteronorbornadienes is feasible under mild conditions, the tandem process can be extended (DA/iEDDA/rDA/rDA) thus simplifying the experimental to a unique step. Altogether, the one-pot or the step-by-step procedure, allows the transfer of the substituents of the starting activated alkyne into the β-positions of a furan or pyrrole. The presence of a halogen on the activated alkyne offers an added value to the strategy given that the halovinyl sulfone embedded into the bicycle can easily accept the attack of different nucleophiles expanding the scope of β-substituents on the final heterocycle. This methodology constitutes one of the very few examples reported for the preparation of synthetically valuable β-halopyrroles/furans.

## Experimental Section

### General Methods

^1^H- and ^13^C NMR spectra were recorded with a Bruker AMX300 spectrometer for solutions in CDCl_3_ and CD_3_OD. δ are given in ppm and *J* in Hz. Chemical shifts are calibrated using residual solvent signals. High resolution mass spectra were recorded on a Q-exactive-quadrupole mass spectrometer. TLC was performed on silica gel 60 F_254_ (Merck), with detection by UV light charring with *p*-anisaldehyde, KMnO_4_, ninhydrin, phosphomolybdic acid, or with reagent [(NH_4_)_6_MoO_4_, Ce(SO_4_)_2_, H_2_SO_4_, H_2_O]. Purification by silica gel chromatography was carried out using either hand-packed glass columns (Silica gel 60 Merck, 40–60 and 63–200 μm) or Puriflash XS520 Plus Interchim system with prepacked cartridges.

### Synthesis of β-Substituted Furans/Pyrroles/Fulvene Derivatives 10a–t, 17–24, and 27

*General Procedure A.* To a solution of the corresponding 7-heteronorbornadiene (0.083 M) in the indicated solvent (3 mL/0.25 mmol) for each case (see Supporting Information, SI), 3,6-di-2-pyridyl-1,2,4,5-tetrazine (DPTz) (0.28–0.50 mmol, 1.1–2.0 equiv) was added. The reaction was vigorously stirred at 25–45 °C for the time indicated (5–24 h). Then, the solvent was removed under reduced pressure and purified by column chromatography on silica gel to afford the targeted furan/*N*-Boc-pyrrole derivatives.

*General Procedure B*. To a solution of the corresponding alkyne derivative (0.083 M) and furan or 2,5-dimethylfuran (3.0 mmol, 12 equiv) in the indicated solvent (3 mL/0.25 mmol of alkyne) for each case (see SI), DPTz (0.28–0.50 mmol, 1.1–2.0 equiv) was added. The reaction was vigorously stirred at 45 °C for 1–2 d. Then, the solvent was removed under reduced pressure and purified by column chromatography on silica gel to afford the targeted furan derivatives.

*General Procedure C*. A solution of the corresponding alkyne derivative (0.083 M) and furan/*N*-Boc-pyrrole (3.0 mmol, 12 equiv) in the indicated solvent (3 mL/0.25 mmol of alkyne) for each case, was vigorously stirred at 45 °C overnight. Then, DPTz (0.28–0.50 mmol, 1.1–2.0 equiv) was added, and the mixture stirred at 25–45 °C for 10–24 h. After that, the solvent was removed under reduced pressure and purified by column chromatography on silica gel to afford the targeted furan/*N*-Boc-pyrrole derivatives.

**3-Tosylfuran (10a).**(4b) General procedure A was followed starting from **9a** (75 mg, 0.30 mmol) and DPTz (78 mg, 0.33 mmol) in toluene (3 mL) for 5 h at r.t. Purification by column chromatography (EtOAc:Cy, 1:4 → 1:1), afforded **10a** (59 mg, 0.27 mmol, 88%, white solid). ^1^H NMR (300 MHz, CDCl_3_, 298 K, δ ppm, *J* Hz): δ 7.97–7.96 (m, 1H), 7.84–7,81 (m, 2H), 7.42–7.41 (m, 1H), 7.32–7.29 (m, 2H), 6.58–6.57 (m, 1H), 2.42 (s, 3H).

**3-Bromo-4-tosylfuran (10b)**.^4b^ General procedure A was followed starting from **9b** (75 mg, 0.23 mmol) and DPTz (108 mg, 0.46 mmol) in toluene (3 mL) for 18 h at r.t. Purification by flash automated chromatography (EtOAc:Cy, 1:24 → 5:3), afforded **10b** (56 mg, 0.19 mmol, 81%, white solid).

General procedure C was followed starting from **2** (60 mg, 0.23 mmol) and furan (0.2 mL, 2.8 mmol) in toluene (3 mL) for 1 d at 45 °C (oil bath). Then, DPTz (109 mg, 0.461 mmol) was added, stirring at r.t. for 15 h. Purification by column chromatography (EtOAc:Cy 1:8 → 1:3), afforded **10b** (62 mg, 0.21 mmol, 89%, white solid). ^1^H NMR (300 MHz, CDCl_3_, 298 K, δ ppm, *J* Hz): δ 8.06 (d, 1H, *J*_*H,H*_ = 1.8), 7.92–7.89 (m, 2H), 7.44 (d, 1H, *J*_*H,H*_ = 1.8), 7.34–7.31 (m, 2H), 2.42 (s, 3H).

**3-Chloro-4-tosylfuran (10c).**^**4b**^ General procedure C was followed starting from **3** (50 mg, 0.23 mmol) and furan (0.2 mL, 2.8 mmol) in toluene (3 mL) for 20 h at 45 °C (oil bath). Then, DPTz (110 mg, 0.465 mmol) was added, stirring at r.t. for 19 h. Purification by column chromatography (EtOAc:Cy, 1:10 → 1:1), afforded **10c** (43 mg, 0.17 mmol, 73%, white solid). ^1^H NMR (300 MHz, CDCl_3_, 298 K, δ ppm, *J* Hz): δ 8.04 (d, 1H, *J*_*H,H*_ = 1.9), 7.92–7.89 (m, 2H), 7.43 (d, 1H, *J*_*H,H*_ = 1.9), 7.34–7.32 (m, 2H), 2.43 (s, 3H).

**3-Iodo-4-tosylfuran (10d).**^**4b**^ General procedure B was followed starting from **4** (75 mg, 0.24 mmol), furan (0.2 mL, 2.8 mmol) and DPTz (116 mg, 0.490 mmol) in toluene (3 mL) for 2 d at 45 °C (oil bath) in a pressure tube. Purification by column chromatography (Et_2_O:Cy, 1:4 → 2:1), afforded **10d** (70 mg, 0.204 mmol, 85%, white solid).^15^

General procedure C was followed starting from 4 (77 mg, 0.25 mmol) and furan (0.420 mL, 6.0 mmol) in toluene (3 mL) for 1 d at 45 °C (oil bath). Then, DPTz (90 mg, 0.38 mmol) was added, stirring at r.t. for 17 h. Purification by column chromatography (DCM: Cy 2:1), afforded **10d** (79 mg, 0.23 mmol, 90%, white solid). ^1^H NMR (300 MHz, CDCl_3_, 298 K, δ ppm, *J* Hz): δ 8.08 (d, 1H, *J*_*H,H*_ = 1.8), 7.94–7.91 (m, 2H), 7.43 (d, 1H, *J*_*H,H*_ = 1.8), 7.34–7.31 (m, 2H), 2.43 (s, 3H).

**Ethyl 4-(Trifluoromethyl)furan-3-carboxylate (10e)**.^13c,16^ General procedure A was followed starting from **9e** (54 mg, 0.23 mmol) and DPTz (109 mg, 0.461 mmol) in DCM (3 mL) for 6 h at r.t. Purification by column chromatography (DCM:*n*-pentane, 1:1), afforded **10e** (39 mg, 0.19 mmol, 81%, colorless oil). ^1^H NMR (300 MHz, CDCl_3_, 298 K, δ ppm, *J* Hz): δ 8.06–8.05 (m, 1H), 7.79–7.78 (m, 1H), 4.33 (q, 2H, *J*_*H,H*_ = 7.1), 1.35 (t, 3H, *J*_*H,H*_ = 7.1).

**Dimethyl Furan-3,4-dicarboxylate (10f).**(17) General procedure A was followed starting from **9f** (75 mg, 0.36 mmol) and DPTz (93 mg, 0.39 mmol) in toluene (3 mL) for 10 h at r.t. Purification by flash automated chromatography (Et_2_O:Cy 1:10 → 9:1), afforded **10f** (60 mg, 0.32 mmol, 91%, white solid).

General procedure B was followed starting from **6** (40 mg, 0.28 mmol), furan (0.24 mL, 3.36 mmol) and DPTz (133 mg, 0.563 mmol) in toluene (3 mL) for 22 h at 45 °C (oil bath) in a pressure tube. Purification by column chromatography (Et_2_O:Cy, 1:2 → 2:1) afforded **10f** (32 mg, 0.17 mmol, 62%, white solid). ^1^H NMR (300 MHz, CDCl_3_, 298 K, δ ppm, *J* Hz): δ 7.93 (s, 2H), 3.84 (s, 6H).

**Methyl 4-Bromofuran-3-carboxylate (10g).**(4b) General procedure A was followed starting from **9g** (98 mg, 0.42 mmol) and DPTz (130 mg, 0.550 mmol) in DCM (3 mL) for 1 d at r.t. Purification by column chromatography (Et_2_O:*n*-pentane, 1:2), afforded **10g** (72 mg, 0.35 mmol, 83%, white solid). ^1^H NMR (300 MHz, CDCl_3_, 298 K, δ ppm, *J* Hz): δ 7.97 (d, 1H, *J*_*H,H*_ = 1.9), 7.46 (d, 1H, *J*_*H,H*_ = 1.9), 3.85 (s, 3H).

**Dimethyl 2,5-dimethylfuran-3,4-dicarboxylate (10h).**(18) General procedure B was followed starting from **6** (50 mg, 0.35 mmol), 2,5-dimethylfuran (0.2 mL, 1.8 mmol) and DPTz (108 mg, 0.457 mmol) in DCM (3 mL)) for 18 h at 45 °C (oil bath) in a pressure tube. Purification by column chromatography (Et_2_O:*n*-pentane, 1:3), afforded **10h** (38 mg, 0.18 mmol, 51%, yellowish oil). ^1^H NMR (300 MHz, CDCl_3_, 298 K, δ ppm, *J* Hz): δ 3.75 (s, 6H), 2.36 (s, 6H). ^13^C{^1^H} NMR (75 MHz, CDCl_3_, 298 K, δ ppm): δ 164.0, 155.8, 113.4, 51.7, 13.1. HRESIMS *m*/*z*: found, 235.0576; calcd. for C_10_H_12_O_5_Na [M + Na]^+^, 235.0577.

**Dimethyl 2-(((***tert***-butoxycarbonyl)amino)methyl)furan-3,4-dicarboxylate (10i).** General procedure A was followed starting from **9i** (100 mg, 0.29 mmol) and DPTz (91 mg, 0.38 mmol) in DCM (3 mL) for 1 d at r.t. Purification by column chromatography (Et_2_O:Cy, 1:4 → 2:1), afforded **10i** (84 mg, 0.27 mmol, 91%, white solid). ^1^H NMR (300 MHz, CDCl_3_, 298 K, δ ppm, *J* Hz): δ 7.79 (s, 1H), 5.17 (br. s, 1H), 4.48 (d, 1H, *J*_*H,H*_ = 5.9), 3.86 (s, 3H), 3.81 (s, 3H), 1.41 (s, 9H). ^13^C{^1^H} NMR (75 MHz, CDCl_3_, 298 K, δ ppm): δ 163.1, 162.1, 158.6, 155.5, 146.4, 118.8, 114.1, 80.0, 52.2, 52.0, 36.9, 28.3. HRESIMS *m*/*z*: found, 336.1051; calcd. for C_14_H_19_O_7_NNa [M + Na]^+^, 336.1054.

**Diethyl (4-Bromofuran-3-yl)phosphonate (10j).** General procedure A was followed starting from **9j** (100 mg, 0.324 mmol) and DPTz (99 mg, 0.42 mmol) in DCM (3 mL) for 4 h at r.t. Purification by column chromatography (DCM → DCM:Acetone, 9:1), afforded **10j** (73 mg, 0.26 mmol, 80%, colorless oil). ^1^H NMR (300 MHz, CDCl_3_, 298 K, δ ppm, *J* Hz): δ 7.81 (t, 1H, *J*_*H,H*_ = 1.9), 7.50 (dd, 1H, *J*_*H,H*_ = 2.4, 1.8), 4.24–4.03 (m, 4H), 1.33 (m, 6H). ^13^C{^1^H} NMR (75 MHz, CDCl_3_, 298 K, δ ppm, *J* Hz): δ 151.6 (d, 1C, *J*_*C,P*_ = 22.7), 143.1 (d, 1C, *J*_*C,P*_ = 12.8), 114.9 (d, 1C, *J*_*C,P*_ = 216.4), 100.3 (d, 1C, *J*_*C,P*_ = 7.9), 62.5 (d, 2C, *J*_*C,P*_ = 5.4), 16.2 (d, 2C, *J*_*C,P*_ = 6.6). HRESIMS *m*/*z*: found, 282.9736; calcd. for C_8_H_13_O_4_^79^BrP [M + H]^+^, 282.9740.

**3-Bromo-2,5-dimethyl-4-tosylfuran (10k).** General procedure A was followed starting from **9k** (100 mg, 0.297 mmol) and DPTz (91 mg, 0.39 mmol) in DCM (3 mL) for 1 d at r.t. Purification by column chromatography (DCM → DCM:Acetone, 15:1), afforded **10k** (56 mg, 0.18 mmol, 60%, colorless oil). ^1^H NMR (300 MHz, CDCl_3_, 298 K, δ ppm, *J* Hz): δ 4.20–3.98 (m, 4H), 2.53 (d, 3H, *J*_*H,H*_ = 2.2), 2.24 (s, 3H), 1.33 (t, 6H, *J*_*H,H*_ = 7.1). ^13^C{^1^H} NMR (75 MHz, CDCl_3_, 298 K, δ ppm, *J* Hz): δ 160.6 (d, 1C, *J*_*C,P*_ = 25.1), 148.9 (d, 1C, *J*_*C,P*_ = 13.0), 108.0 (d, 1C, *J*_*C,P*_ = 216.6), 96.9 (d, 1C, *J*_*C,P*_ = 8.1), 62.0 (d, 2C, *J*_*C,P*_ = 5.1), 16.2 (d, 2C, *J*_*C,P*_ = 6.8), 14.1, 11.6. HRESIMS *m*/*z*: found, 311.0048; calcd. for C_10_H_17_O_4_^79^BrP [M + H]^+^, 311.0053.

**Diethyl (4-Bromo-2-(butyramidomethyl)furan-3-yl)phosphonate (10l) and Diethyl (4-Bromo-5-(butyramidomethyl)furan-3-yl)phosphonate (10m).** General procedure A was followed starting from a 5:1 regioisomeric mixture of **9l** and **9m** (100 mg, 0.245 mmol) and DPTz (75 mg, 0.32 mmol) in DCM (3 mL) for 6 h at r.t. Purification by flash automated chromatography (DCM:MeOH:NH_4_OH, 150:1:0.1 → 85:14:1), afforded **10l** (72 mg, 0.19 mmol, 77%), and **10m** (11 mg, 0.029 mmol, 12%), both as colorless oils.

Data for compound **10l**: ^1^H NMR (300 MHz, CDCl_3_, 298 K, δ ppm, *J* Hz): δ 7.38 (d, 1H, *J*_*H,P*_ = 2.2), 7.13 (br. s, 1H), 4.63 (dd, 2H, *J* = 6.3, 1.4), 4.21–4.00 (m, 4H), 2.14 (t, 2H, *J*_*H,H*_ = 7.3), 1.62 (sextet, 2H, *J*_*H,H*_ = 7.5), 1.34 (t, 6H, *J*_*H,H*_ = 7.1), 0.90 (t, 3H, *J*_*H,H*_ = 7.4). ^13^C{^1^H} NMR (75 MHz, CDCl_3_, 298 K, δ ppm, *J* Hz): δ 172.6, 163.1 (d, 1C, *J*_*C,P*_ = 25.7), 140.7 (d, 1C, *J*_*C,P*_ = 12.8), 109.5 (d, 1C, *J*_*C,P*_ = 213.9), 101.0 (d, 1C, *J*_*C,P*_ = 7.4), 62.5 (d, 2C, *J*_*C,P*_ = 5.2), 38.4, 36.2, 18.9, 16.3 (d, 2C, *J*_*C,P*_ = 6.8), 13.8. Data for compound **10m**: ^1^H NMR (300 MHz, CDCl_3_, 298 K, δ ppm, *J* Hz): δ 7.78 (d, 1H, *J*_*H,P*_ = 2.3), 5.83 (br. s, 1H), 4.50 (d, 2H, *J*_*H,H*_ = 5.6), 4.24–4.05 (m, 4H), 2.19 (t, 2H, *J*_*H,H*_ = 7.3), 1.74–1.61 (m, 2H), 1.35 (t, 6H, *J*_*H,H*_ = 7.1), 0.94 (t, 3H, *J*_*H,H*_ = 7.4). ^13^C{^1^H} NMR (75 MHz, CDCl_3_, 298 K, δ ppm, *J* Hz): δ 172.9, 151.1 (d, 1C, *J*_*C,P*_ = 12.5), 150.4 (d, 1C, *J*_*C,P*_ = 22.2), 115.6 (d, 1C, *J*_*C,P*_ = 216.7), 98.6 (d, *J*_*C,P*_ = 7.8), 62.6 (d, 2C, *J*_*C,P*_ = 5.4), 38.4, 34.4, 19.0, 16.3 (d, 2C, *J*_*C,P*_ = 6.7), 13.7. HRESIMS *m*/*z*: found, 382.0405; calcd. for C_13_H_21_O_5_N^79^BrP [M + H]^+^, 382.0413.

***tert*-Butyl 3-tosyl-1H-pyrrole-1-carboxylate (10n).**(4b) General procedure A was followed starting from **9n** (75 mg, 0.22 mmol) and DPTz (56 mg, 0.24 mmol) in toluene (3 mL) for 1 d at 45 °C (oil bath). Purification by column chromatography (EtOAc:Cy, 1:4 → 1:1), afforded **10n** (59 mg, 0.18 mmol, 82%, white solid). ^1^H NMR (300 MHz, CDCl_3_, 298 K, δ ppm, *J* Hz): δ 7.84–7.80 (m, 2H), 7.78 (ap. dd, 1H, *J*_*H,H*_ = 1.8, *J*_*H,H*_ = 2.2), 7.30–7.27 (m, 2H), 7.21 (dd, 1H, *J*_*H,H*_ = 2.2, *J*_*H,H*_ = 3.4), 6.43 (dd, 1H, *J*_*H,H*_ = 1.8, *J*_*H,H*_ = 3.4), 2.39 (s, 3H), 1.58 (s, 9H).

***tert*****-Butyl 3-bromo-4-tosyl-1H-pyrrole-1-carboxylate (10o).**(4b) General procedure A was followed starting from **9o** (100 mg, 0.23 mmol) and DPTz (73 mg, 0.30 mmol) in toluene (2 mL) and DCM (1 mL) for 16 h at 45 °C. Purification by column chromatography (DCM:Cy, 1:1), afforded **10o** (79 mg, 0.20 mmol, 84%, yellowish solid).

General procedure C was followed starting from **2** (65 mg, 0.25 mmol) and *N*-Boc-pyrrole (0.5 mL, 3.0 mmol) in toluene (3 mL) for 6 h at 70 °C. Then, DPTz (120 mg, 0.51 mmol) was added, stirring at 45 °C for 22 h. Purification by column chromatography (Et_2_O:Cy 1:2), afforded **10o** (74 mg, 0.19 mmol, 74%, yellowish solid). ^1^H NMR (300 MHz, CDCl_3_, 298 K, δ ppm, *J* Hz): δ 7.91–7.88 (m, 3H), 7.31–7.28 (m, 2H), 7.26–7.25 (d, 1H, *J*_*H,H*_ = 2.6), 2.40 (s, 3H), 1.59 (s, 9H).

***tert*****-Butyl 3-chloro-4-tosyl-1H-pyrrole-1-carboxylate (10p).** General procedure A was followed starting from **9p** (103 mg, 0.27 mmol) and DPTz (82 mg, 0.35 mmol) in toluene (2 mL) and DCM (1 mL) for 17 h at 45 °C (oil bath). Purification by column chromatography (DCM:Cy, 1:1), afforded **10p** (79 mg, 0.22 mmol, 82%, yellowish oil). ^1^H NMR (300 MHz, CDCl_3_, 298 K, δ ppm, *J* Hz):  δ 7.89 (ap. d, 2H, *J*_*H,H*_ = 8.3), 7.86 (d, 1H, *J*_*H,H*_ = 2.6), 7.30 (ap. d, 2H, *J*_*H,H*_ = 8.0), 7.18 (d, 1H, *J*_*H,H*_ = 2.6), 2.40 (s, 3H), 1.59 (s, 9H). ^13^C{^1^H} NMR (75 MHz, CDCl_3_, 298 K, δ ppm): δ 146.5, 144.3, 138.1, 129.6, 127.8, 126.4, 124.2, 119.6, 113.1, 86.6, 27.7, 21.5. HRESIMS *m*/*z*: found, 378.0530; calcd. for C_16_H_18_O_4_N^35^ClNaS [M + Na]^+^, 378.0537.

**1-(*****tert*****-butyl) 3,4-dimethyl 1H-pyrrole-1,3,4-tricarboxylate (10q)**.^13g^ General procedure C was followed starting from **6** (43 μL, 0.35 mmol) and *tert*-butyl 1H-pyrrole-1-carboxylate (0.70 mL, 4.2 mmol) in toluene (3 mL) for 1 d at 45 °C (oil bath). Then, DPTz (92 mg, 0.39 mmol) was added and stirred at 45 °C for 22 h. Purification by flash automated chromatography (EtOAc:Cy, 1:24 → 4:1), afforded **10q** (70 mg, 0.25 mmol, 71%, yellowish oil). ^1^H NMR (300 MHz, CDCl_3_, 298 K, δ ppm, *J* Hz): δ 7.73 (s, 2H), 3.84 (s, 6H), 1.61 (s, 9H). ^13^C{^1^H} NMR (75 MHz, CDCl_3_, 298 K, δ ppm): δ 163.2, 147.1, 125.9, 118.1, 86.2, 51.8, 27.8. HRESIMS *m*/*z*: found, 306.0941; calcd. for C_13_H_17_O_6_NNa [M + Na]^+^, 306.0948.

**1-(*****tert*****-Butyl) 3-Methyl 4-bromo-1H-pyrrole-1,3-dicarboxylate (10r).** General procedure A was followed starting from **9r** (70 mg, 0.21 mmol) and DPTz (100 mg, 0.423 mmol) in toluene (3 mL) for 1 d at r.t. Purification by column chromatography (DCM:Cy, 2:1), afforded **10r** (55 mg, 0.18 mmol, 86%, yellowish solid). ^1^H NMR (300 MHz, CDCl_3_, 298 K, δ ppm, *J* Hz):  δ 7.79 (d, 1H, *J*_*H,H*_ = 2.6), 7.26 (d, 1H, *J*_*H,H*_ = 2.6), 3.83 (s, 1H), 1.59 (s, 9H). ^13^C{^1^H} NMR (75 MHz, CDCl_3_, 298 K, δ ppm): δ 162.8, 146.9, 125.6, 121.3, 117.2, 100.0, 85.8, 51.3, 27.7. HRESIMS *m*/*z*: found, 325.9993; calcd. for C_11_H_14_O_4_N^79^BrNa [M + Na]^+^, 325.9998.

**1-(*****tert*****-Butyl) 3-Ethyl 4-(trifluoromethyl)-1H-pyrrole-1,3-dicarboxylate (10s).** General procedure A was followed starting from **9s** (77 mg, 0.23 mmol) and DPTz (71 mg, 0.30 mmol) in toluene (3 mL) for 16 h at 45 °C (oil bath). Purification by column chromatography (DCM:Cy, 1:1), afforded **10s** (53 mg, 0.17 mmol, 74%, white solid). ^1^H NMR (300 MHz, CDCl_3_, 298 K, δ ppm, *J* Hz): δ 7.89–7.88 (m, 1H), 7.57–7.56 (m, 1H), 4.32 (q, *J*_*H,H*_ = 7.1, 2H), 1.63 (s, 9H), 1.35 (t, 3H, *J*_*H,H*_ = 7.1). ^13^C{^1^H} NMR (75 MHz, CDCl_3_, 298 K, δ ppm, *J* Hz): δ 161.9, 147.0, 127.2, 122.0 (q, 1C, *J*_*C,F*_ = 6.6), 121.9 (q, 1C, *J*_*C,F*_ = 267.2), 116.8 (q, 1C, *J*_*C,F*_ = 37.6), 116.29–116.27 (m, 1C), 86.5, 60.7, 27.7, 14.0. HRESIMS *m*/*z*: found, 330.0924; calcd. for C_13_H_16_O_4_NF_3_Na [M + Na]^+^, 330.0924.

**1-((5-Bromo-3-(propan-2-ylidene)cyclopenta-1,4-dien-1-yl)sulfonyl)-4-methylbenzene (10t).** General procedure A was followed starting from **9t** (85 mg, 0.23 mmol) and DPTz (60 mg, 0.26 mmol) in toluene (2 mL) and DCM (1 mL) for 14 h at r.t. Purification by column chromatography (DCM:Cy, 2:1), afforded **10t** (70 mg, 0.21 mmol, 89%, brownish solid). ^1^H NMR (300 MHz, CDCl_3_, 298 K, δ ppm, *J* Hz): δ 7.90–7.87 (m, 2H), 7.44 (d, 1H, *J*_*H,H*_ = 2.8), 7.29 (ap. d, 2H, *J*_*H,H*_ = 8.0), 6.67 (d, 1H, *J*_*H,H*_ = 2.8), 2.40 (s, 3H), 2.28 (s, 3H), 2.22 (s, 3H). ^13^C{^1^H} NMR (75 MHz, CDCl_3_, 298 K, δ ppm): δ 162.9, 144.1, 140.5, 138.1, 137.7, 129.1, 128.6, 128.3, 124.0, 113.9, 23.9, 23.5, 21.6. HRESIMS *m*/*z*: found, 360.9868; calcd. for C_15_H_15_O_2_^79^BrNaS [M + Na]^+^, 360.9874.

***N*****-(2-((4-Tosylfuran-3-yl)thio)ethyl)acetamide (17).** General procedure A was followed starting from **12** (85 mg, 0.23 mmol) and DPTz (110 mg, 0.466 mmol) in water (3 mL) for 18 h at r.t. Purification by flash automated chromatography (EtOAc:Cy, 1:8 → 9:1), afforded **17** (60 mg, 0.18 mmol, 76%, brownish oil). ^1^H NMR (300 MHz, CDCl_3_, 298 K, δ ppm, *J* Hz): δ 8.03 (d, 1H, *J*_*H,H*_ = 1.8), 7.92–7.89 (m, 2H), 7.52 (d, 1H, *J*_*H,H*_ = 1.8), 7.33–7.30 (m, 2H), 6.62 (br. s, 1H), 3.35 (q, 2H, *J*_*H,H*_ = 6.2), 2.87 (t, 2H, *J*_*H,H*_ = 6.2), 2.40 (s, 3H), 1.96 (s, 3H). ^13^C{^1^H} NMR (75 MHz, CDCl_3_, 298 K, δ ppm): δ 170.5, 148.2, 148.1, 145.0, 137.7, 131.2, 129.8, 127.0, 114.8, 38.0, 36.6, 23.2, 21.7. HRESIMS *m*/*z*: found, 362.0485; calcd. for C_15_H_17_O_4_NNaS_2_ [M + Na]^+^, 362.0491.

***tert*****-Butyl-3-((2-((*****tert*****-butoxycarbonyl)amino)ethyl)thio)-4-tosyl-1H-pyrrole-1-carboxylate (18)**.^4b^ General procedure A was followed starting from **13** (125 mg, 0.239 mmol) and DPTz (73 mg, 0.31 mmol) in DCM (3 mL) for 11 h at r.t. Purification by column chromatography (EtOAc:Cy, 1:6), afforded **18** (108 mg, 0.22 mmol, 91%, yellowish oil). ^1^H NMR (300 MHz, CDCl_3_, 298 K, δ ppm, *J* Hz):  δ 7.92 (ap. d, 2 H, *J*_*H,H*_ = 8.3), 7.88 (d, 1H, *J*_*H,H*_ = 2.5), 7.30–7.27 (m, 3H), 5.14 (br. s, 1H), 3.23–3.19 (m, 2H), 2.84 (t, 2H, *J*_*H,H*_ = 6.3), 2.39 (s, 3H), 1.59 (s, 9H), 1.42 (s, 9H). ^13^C{^1^H} NMR (75 MHz, CDCl_3_, 298 K, δ ppm): δ 155.8, 146.8, 144.2, 138.5, 129.9, 129.5, 128.0, 125.8, 125.7, 114.7, 85.4, 79.2, 39.3, 36.6, 28.4, 27.8, 21.5. HRESIMS *m*/*z*: found, 519.1590; calcd. for C_23_H_32_O_6_N_2_NaS_2_ [M + Na]^+^, 519.1594.

***N*****-(2-((3-(Propan-2-ylidene)-5-tosylcyclopenta-1,4-dien-1-yl)thio)ethyl)acetamide (19).** General procedure A was followed starting from **14** (405 mg, 1.00 mmol) and DPTz (261 mg, 1.10 mmol) in DCM (12 mL) for 5.5 h at r.t. Then, DCM was added and the organic layer was washed with 1 M aq. HCl and brine. The organic layer was dried on anhydrous Na_2_SO_4_, filtered and concentrated under reduced pressure. Purification by column chromatography (EtOAc), afforded **19** (336 mg, 0.890 mmol, 89%, orange solid). ^1^H NMR (300 MHz, CDCl_3_, 298 K, δ ppm, *J* Hz): δ 7.90–7.87 (m, 2H), 7.40 (d, 1H, *J*_*H,H*_ = 2.6), 7.29 (ap. d, 2H, *J*_*H,H*_ = 8.1), 6.50 (d, 1H, *J*_*H,H*_ = 2.6), 6.35 (br. s, 1H, *J*_*H,H*_ = 5.8), 3.48–3.41 (m, 2H), 2.96 (t, 2H, *J*_*H,H*_ = 6.4), 2.40 (s, 3H), 2.26 (s, 3H), 2.24 (s, 3H), 1.93 (s, 3H). ^13^C{^1^H} NMR (75 MHz, CDCl_3_, 298 K, δ ppm): δ 170.4, 159.5, 144.1, 141.9, 138.5, 138.0, 133.0, 129.53, 129.50, 128.0, 119.2, 37.9, 33.8, 23.7, 23.5, 23.1, 21.6. HRESIMS *m*/*z*: found, 400.1004; calcd. for C_19_H_23_O_3_NNaS_2_ [M + Na]^+^, 400.1012.

**Diethyl (4-((2-Acetamidoethyl)thio)furan-3-yl)phosphonate (20).** General procedure A was followed starting from **15** (50 mg, 0.14 mmol) and DPTz (44 mg, 0.19 mmol) in DCM (3 mL) for 4 h at r.t. Purification by column chromatography (DCM → DCM:MeOH, 50:1), afforded **20** (41 mg, 0.13 mmol, 93%, colorless oil). ^1^H NMR (300 MHz, CDCl_3_, 298 K, δ ppm, *J* Hz): δ 7.73 (t, 1H, *J*_*H,H*_ = 1.8), 7.61–7.58 (m, 2H), 4.23–4.14 (m, 4H), 3.35–3.30 (m, 2H), 2.92–2.88 (m, 2H), 1.96 (s, 3H), 1.36 (t, 6H, *J*_*H,H*_ = 7.1). ^13^C{^1^H} NMR (75 MHz, CDCl_3_, 298 K, δ ppm, *J* Hz): δ 170.7, 150.2 (d, 1C, *J*_*C,P*_ = 22.7), 148.0 (d, 1C, *J*_*C,P*_ = 14.7), 117.2 (d, 1C, *J*_*C,P*_ = 217.9), 116.6 (d, 1C, *J*_*C,P*_ = 12.4), 62.7 (d, 2C, *J*_*C,P*_ = 5.9), 37.9, 37.4, 23.0, 16.4 (d, 2C, *J*_*C,P*_ = 6.5). HRESIMS *m*/*z*: found, 322.0884; calcd. for C_12_H_21_O_5_NPS [M + H]^+^, 322.0876.

**Diethyl (4-((2-Acetamidoethyl)thio)-2,5-dimethylfuran-3-yl)phosphonate (21).** General procedure A was followed starting from **16** (50 mg, 0.13 mmol) and DPTz (41 mg, 0.17 mmol) in DCM (3 mL) for 18 h at r.t. Purification by flash automated chromatography (DCM:MeOH:NH_4_OH, 150:1:0.1 → 85:14:1), afforded **21** (32 mg, 0.091 mmol, 69%, colorless oil). ^1^H NMR (300 MHz, CDCl_3_, 298 K, δ ppm, *J* Hz): δ 7.84 (br. s, 1H), 4.19–4.09 (m, 4H), 3.27 (q, 2H, *J*_*H,H*_ = 5.7), 2.78–2.74 (m, 2H), 2.42 (d, 3H, *J*_*H,H*_ = 2.3), 2.31 (s, 3H), 1.96 (s, 3H), 1.35 (t, 6H, *J*_*H,H*_ = 7.1). ^13^C{^1^H} NMR (75 MHz, CDCl_3_, 298 K, δ ppm, *J* Hz): δ 170.6, 158.1 (d, 1C, *J*_*C,P*_ = 24.3), 156.1 (d, 1C, *J*_*C,P*_ = 15.0), 111.0 (d, 1C, *J*_*C,P*_ = 218.8), 110.8 (d, 1C, *J*_*C,P*_ = 12.9), 62.2 (d, 2C, *J*_*C,P*_ = 5.8), 37.8, 36.3, 23.2, 16.4 (d, 2C, *J*_*C,P*_ = 6.8), 14.1, 11.7. HRESIMS *m*/*z*: found, 350.1197; calcd. for C_14_H_25_O_5_NPS [M + H]^+^, 350.1187.

***N,N*****-Diethyl-4-tosylfuran-3-amine (22).** To a stirred solution of **9b** (100 mg, 0.31 mmol) in anhydrous MeCN (3 mL) under Ar, Et_3_N (47 μL, 0.34 mmol) and Et_2_NH (36 μL, 0.34 mmol) were added. The reaction mixture was stirred for 40 min at r.t. Then, DPTz (110 mg, 0.466 mmol) was added and the reaction mixture was heated to 45 °C (oil bath) and vigorously stirred for 18 h. The solvent was removed under reduced pressure and the crude was purified by chromatography column on silica gel (EtOAc:Cy, 1:8) to afford **22** (41 mg, 0.14 mmol, 46%, orange solid). ^1^H NMR (300 MHz, CDCl_3_, 298 K, δ ppm, *J* Hz): δ 7.90–7.86 (m, 3H), 7.29–7.26 (m, 2H), 7.05 (s, 1H), 2.98 (q, 4H, *J*_*H,H*_ = 7.1), 2.40 (s, 3H), 0.86 (t, 6H, *J*_*H,H*_ = 7.1). ^13^C{^1^H} NMR (75 MHz, CDCl_3_, 298 K, δ ppm): δ 147.8, 144.0, 138.7, 135.6, 135.3, 129.3, 127.7, 126.3, 47.0, 21.6, 11.3. HRESIMS *m*/*z*: found, 294.1160; calcd. for C_15_H_20_NO_3_S [M + H]^+^, 294.1158.

***tert*****-Butyl 3-(diethylamino)-4-tosyl-1H-pyrrole-1-carboxylate (23).** To a stirred solution of **9o** (150 mg, 0.35 mmol) in anhydrous MeCN (2 mL) under Ar, Et_3_N (53 μL, 0.38 mmol) and Et_2_NH (40 μL, 0.38 mmol) were added. The reaction mixture was stirred for 2 h. Then, DPTz (110 mg, 0.466 mmol) was added and the reaction mixture was heated to 45 °C (oil bath) and vigorously stirred for 6 h. The solvent was removed under reduced pressure and the crude was purified by chromatography column on silica gel (Et_2_O:Cy, 1:1) to afford **23** (70 mg, 0.18 mmol, 51%, orange oil). ^1^H NMR (300 MHz, CDCl_3_, 298 K, δ ppm, *J* Hz): δ 7.89 (ap. d, 2H, *J*_*H,H*_ = 8.4), 7.77 (d, 1H, *J*_*H,H*_ = 2.6), 7.25 (ap. d, 2H, *J*_*H,H*_ = 8.2), 6.77 (d, 1H, *J*_*H,H*_ = 2.3), 2.96 (q, 4H, *J*_*H,H*_ = 7.1), 2.39 (s, 3H), 1.60 (s, 9H), 0.84 (t, 6H, *J*_*H,H*_ = 7.1). ^13^C{^1^H} NMR (75 MHz, CDCl_3_, 298 K, δ ppm): δ 147.6, 143.5, 139.2, 136.7, 129.0, 127.6, 125.0, 124.3, 112.2, 85.3, 47.4, 27.8, 26.9, 21.5, 11.4. HRESIMS *m*/*z*: found, 393.1835; calcd. for C_20_H_29_N_2_O_4_S [M + H]^+^, 393.1843.

**1-(4-Tosylfuran-3-yl)pyridin-1-ium Bromide (24).** To a solution of pyridine (32 μL, 0.39 mmol) in toluene (1 mL), a solution of **9b** (75 mg, 0.23 mmol) in toluene (1 mL) was added. The reaction was stirred at r.t. overnight and concentrated under reduced pressure. Then, the residue was dissolved in DCM (3 mL) and DPTz (108 mg, 0.457 mmol) was added. The reaction was vigorously stirred at r.t. for 2.5 h. The reaction mixture was filtered to afford **24** (74 mg, 0.195 mmol, 85%, white solid). ^1^H NMR (300 MHz, CD_3_OD, 298 K, δ ppm, *J* Hz): δ 9.04–9.01 (m, 2H), 8.92 (tt, 1H, *J*_*H,H*_ = 8.0, *J*_*H,H*_ = 1.3), 8.64 (d, 1H, *J*_*H,H*_ = 1.7), 8.51 (d, 1H, *J*_*H,H*_ = 1.7), 8.33–8.29 (m, 2H), 7.55–7.52 (m, 2H), 7.42–7.39 (m, 2H), 2.46 (s, 3H). ^13^C{^1^H} NMR (75 MHz, CD_3_OD, 298 K, δ ppm): δ 148.2, 148.1, 146.8, 145.6, 142.9, 136.4, 129.7, 127.3, 126.5, 126.1, 125.1, 19.6. HRESIMS *m*/*z*: found, 300.0681; calcd. for C_16_H_14_O_3_NaS [M-Br]^+^, 300.0689.

**Diethyl (4-Methoxyfuran-3-yl)phosphonate (27).** General procedure A was followed starting from **26** (45 mg, 0.17 mmol) and DPTz (53 mg, 0.22 mmol) in DCM (3 mL) for 4 h at r.t. Then, it was diluted with DCM, washed with 1M HCl (×5), with sat. aq. NaHCO_3_ and with brine. The organic layer was dried with Na_2_SO_4_, filtered and concentrated in vacuo to afford **27** (33 mg, 0.14 mmol, 81%, brownish oil). ^1^H NMR (300 MHz, CDCl_3_, 298 K, δ ppm, *J* Hz): δ 7.61 (t, 1H, *J*_*H,H*_ = 1.9), 7.10 (dd, 1H, *J*_*H,H*_ = 2.8, 1.7), 4.21–4.02 (m, 4H), 3.73 (s, 3H), 1.31 (t, 6H, *J*_*H,H*_ = 7.1). ^13^C{^1^H} NMR (75 MHz, CDCl_3_, 298 K, δ ppm, *J* Hz): δ 150.1 (d, 1C, *J*_*C,P*_ = 21.0), 149.8 (d, 1C, *J*_*C,P*_ = 3.1), 124.4 (d, 1C, *J*_*C,P*_ = 4.4), 106.8 (d, 1C, *J*_*C,P*_ = 212.0), 62.4 (d, 2C, *J*_*C,P*_ = 5.4), 58.6, 16.3 (d, 2C, *J*_*C,P*_ = 6.5). HRESIMS *m*/*z*: found, 235.0729; calcd. for C_9_H_16_O_5_P [M + H]^+^, 235.0730.

## Data Availability

The data underlying this study are available in the published article and its SI.

## Supporting Information Available

Details of the experimental procedures for the preparation of heteronorbornadienes and NMR spectra for the new compounds (PDF)
