# Supplementary material for: Transferring Substituents from Alkynes to Furans and Pyrroles through Heteronorbornadienes as Intermediates: Synthesis of β-Substituted Pyrroles/Furans
Source: J Org Chem. 2023 Aug 24;88(18):13331–8. doi: 10.1021/acs.joc.3c01145 (PMC10507663; doi:10.1021/acs.joc.3c01145)
Supplement: Supplementary file 1 — jo3c01145_si_001.pdf [file jo3c01145_si_001.pdf]

SUPPORTING INFORMATION

Transferring Substituents from Alkynes to Furans and Pyrroles through Heteronorbornadienes as Intermediates: Synthesis of β-Substituted Pyrroles/Furans

Javier García-Domínguez,[+] Marina Carranza,[+] Edijs Jansons, Ana T. Carmona,* Inmaculada Robina and Antonio J. Moreno-Vargas*

Departamento de Química Orgánica, Facultad de Química, Universidad de Sevilla, 41012 Sevilla, Spain

E-mail: anatere@us.es, ajmoreno@us.es

[+]These authors contributed equally to this work

TABLE OF CONTENTS

1. Synthesis of activated alkynes **1-8** S2

2. Details for the preparation of cyclic dienes S2

3. Synthesis of heteronorbornadienic systems **9a**, **9b**, **9e-g**, **9i-p**, **9r-t**, **12-16** and **25-26**. S3

4. ^{1}H-NMR spectra for known compounds. S8

5. ^{1}H and ^{13}C-NMR spectra for new compounds. S14

6. References. S43

General methods.

^{1}H- and ^{13}C-NMR spectra were recorded with a Bruker AMX300 spectrometer for solutions in $CDCl_3$, and CD_3OD. δ are given in ppm and *J* in Hz. Chemical shifts are calibrated using residual solvent signals. High resolution mass spectra were recorded on a Q-Exactive spectrometer. TLC was performed on silica gel 60 F_{254} (Merck), with detection by UV light charring with *p*-anisaldehyde, $KMnO_4$, ninhydrin, phosphomolybdic acid or with reagent [$(NH_4)_6MoO_4$, $Ce(SO_4)_2$, H_2SO_4, H_2O]. Purification by silica gel chromatography was carried out using either hand-packed glass columns (Silica gel 60 Merck, 40-60 and 63-200 µm) or Puriflash XS520 Plus Interchim system with prepacked cartridges.

1. Synthesis of activated alkynes 1-8

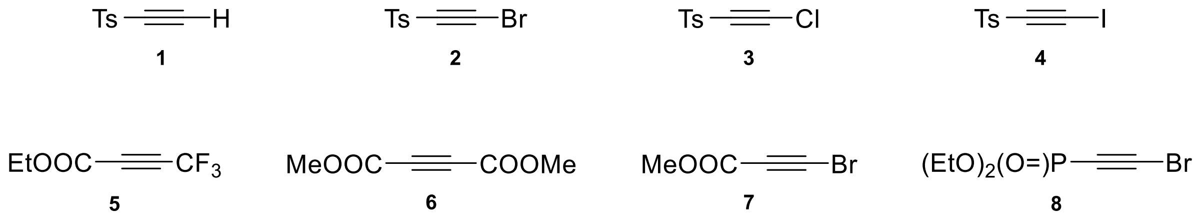

- Alkyne **1** was prepared following a procedure previously described by our research group[1] and previously characterized.[2]
- Alkynes **2**,[3] **3**[4] and **7**[5] were synthesized from commercially available acetylenes, following the standard protocols previously reported.
- Alkyne **4** was previously prepared and characterized in our research group.[6]
- Alkynes **5** and **6** were purchased from Aldrich.
- Alkyne **8** was synthesized following a reported procedure.[7]

2. Details for the preparation of dienes.

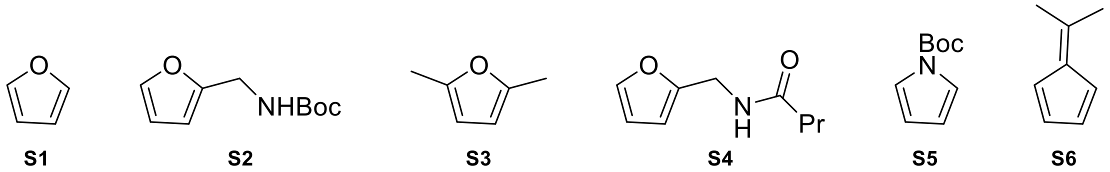

- Dienes **S1**, **S3**, **S5** and **S6** were purchased from Aldrich.
- Diene **S2** was synthesized following a reported procedure.[8]
- Diene **S4** was synthesised here for the first time.

***N*-(furan-2-ylmethyl)butyramide (S4).**

To a solution of furfurylamine (1.8 mL, 21 mmol) in anhydrous dichloromethane (DCM anh., 50 mL) at 0 °C under Ar atmosphere, butyryl chloride (4.3 mL, 41 mmol) and triethylamine (5.7 mL, 41 mmol) were added. The reaction was stirred for 1.5 h at r.t. Then, the reaction mixture was diluted with DCM and it was washed with HCl (1M) and, subsequently, with NH_4Cl (aq. sat.). The organic layer was separated, dried with anhydrous Na_2SO_4, and concentrated in vacuo. The residue was purified by column chromatography on silica gel (EtOAc:Cy, 1:3→ 2:1) to afford **S4** (2.8 g, 17 mmol, 81%, white solid). **^{1}H NMR (300 MHz, $CDCl_3$, 298 K, δ ppm, *J* Hz):** δ 7.32-7.31 (m, 1H), 6.29-6.28 (m, 1H), 6.19-6.18

(m, 1H), 6.07 (br. s, 1H), 4.40 (d, $J_{H,H}$ = 5.4, 2H), 2.16 (t, $J_{H,H}$ = 7.5, 2H), 1.70-1.58 (m, 2H), 0.91 (t, $J_{H,H}$ = 7.4, 3H). **$^{13}C\{^1H\}$ NMR (75 MHz, CDCl$_3$, 298 K, δ ppm):** δ 172.8, 151.4, 142.0, 110.3, 107.2, 38.4, 36.3, 19.0, 13.6. **HRESIMS m/z:** found, 190.0838; calcd. for $C_9H_{13}NO_2$ $[M+H]^+$: 190.0836.

3. Synthesis of heteronorbornadienic systems 9a, 9b, 9e-g, 9i-p, 9r-t, 12-16 and 25-26.

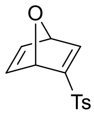

9a

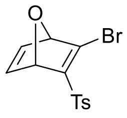

9b

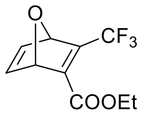

9e

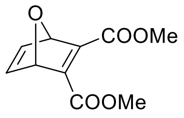

9f

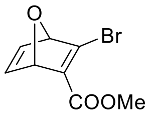

9g

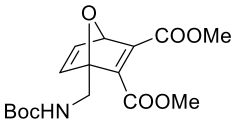

9i

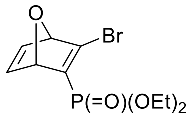

9j

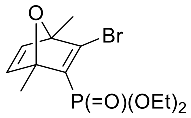

9k

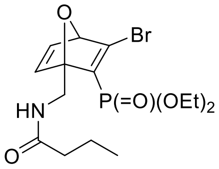

9l

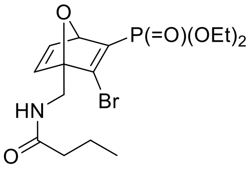

9m

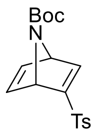

9n

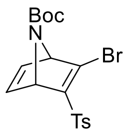

9o

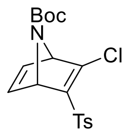

9p

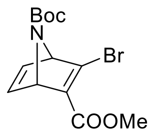

9r

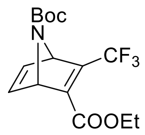

9s

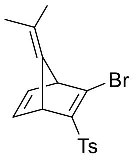

9t

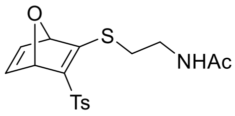

12

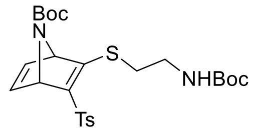

13

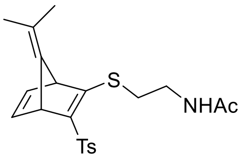

14

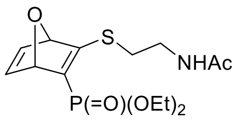

15

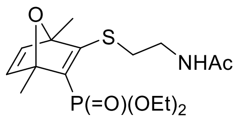

16

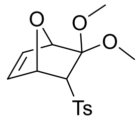

25

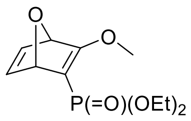

26

- Bicyclic systems **9a**, **9b**, **9e**, **9f**, **9g**, **9i**, **9n**, **9o**, **9p**, **9r** and **9s** were synthesized from alkynes **1-8** and dienes **S1-S6**, following the standard protocols previously reported.[9]

- Byciclic systems **9t** and **13** were prepared according the procedures previously reported by our research group.[10,9t]

- Bicyclic systems **9j**, **9k**, **9l**, **9m**, **12**, **14-16 and 25-26** have been prepared here for the first time.

General procedure for the synthesis of oxanorbornadienic phosphonates (9j -9m).

A solution of alkyne **8** (200 mg, 0.83 mmol) and the corresponding furan (4-12 equiv.) in 3 mL of a EtOH/H_2O mixture (2/3) was stirred in a pressure tube at 70 °C in an oil bath for 16h. Then, the solvent was removed in vacuo and the crude was purified by column chromatography on silica gel.

Diethyl ((*rac*)-3-bromo-7-oxabicyclo[2.2.1]hepta-2,5-dien-2-yl)phosphonate (9j).

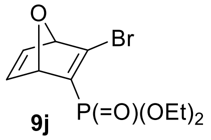

9j

General procedure was followed starting from **S1** (0.72 mL, 10 mmol). Purification (EtOAc:Cy, 1:5 → 2:5) afforded **9j** (120 mg, 0.39 mmol, 47%, yellowish oil). **^{1}H NMR (300 MHz, $CDCl_3$, 298 K, δ ppm, *J* Hz):** δ 7.20-7.15 (m, 2H), 5.68-5.67 (m, 1H), 5.32-5.31 (m, 1H), 4.16-4.00 (m, 4H), 1.35-1.29 (m, 6H). **$^{13}C\{^1H\}$ NMR (75 MHz, $CDCl_3$, 298 K, δ ppm, *J* Hz):** δ 151.5 (d, 1C, $J_{C,P}$ = 6.7), 144.5 (d, 1C, $J_{C,P}$ = 1.4), 141.0 (d, 1C, $J_{C,P}$ = 2.0), 140.0 (d, 1C, $J_{C,P}$ = 212.0) 87.6 (d, 1C, $J_{C,P}$ = 12.7), 86.8 (d, 1C, $J_{C,P}$ = 14.3), 62.4-62.3 (m, 2C), 16.3 (d, 2C, $J_{C,P}$ = 6.6). **HRESIMS m/z:** found, 330.9705; calcd. for $C_{10}H_{14}O_4{}^{79}BrNaP$ $[M+Na]^+$: 330.9706.

Diethyl ((*rac*)-3-bromo-1,4-dimethyl-7-oxabicyclo[2.2.1]hepta-2,5-dien-2-yl)phosphonate (9k).

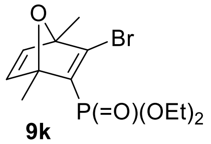

9k

General procedure was followed starting from **S3** (0.36 mL, 3.3 mmol). Purification (EtOAc:Cy, 1:5 → 2:5) afforded **9k** (142 mg, 0.42 mmol, 51%, brownish oil). **^{1}H NMR (300 MHz, $CDCl_3$, 298 K, δ ppm, *J* Hz):** δ 6.90 (d, 1H, $J_{H,H}$ = 5.1), 6.85 (d, 1H, $J_{H,H}$ =5.1), 4.17-3.97 (m, 4H), 1.86 (s, 3H), 1.69 (d, 1H, $J_{H,H}$ =1.1), 1.35-1.29 (m, 6H). **$^{13}C\{^1H\}$ NMR (75 MHz, $CDCl_3$, 298 K, δ ppm, *J* Hz):** δ 157.7 (d, 1C, $J_{C,P}$ = 6.0), 148.4 (d, 1C, $J_{C,P}$ = 1.3), 145.3 (d, 1C, $J_{C,P}$ = 1.7), 142.0 (d, 1C, $J_{C,P}$ = 206.7), 94.6-93.9 (m, 2C), 62.2-62.0 (m, 2C), 16.9-15.6 (m, 2C). **HRESIMS m/z:** found, 359.0018; calcd. for $C_{12}H_{18}O_4{}^{79}BrNaP$ $[M+Na]^+$: 359.0012.

Diethyl((*rac*)-3-bromo-1-(butyramidomethyl)-7-oxabicyclo[2.2.1]hepta-2,5-dien-2-yl)phosphonate (9l) and diethyl((*rac*)-1-(butyramidomethyl)-7-oxabicyclo[2.2.1]hepta-2,5-diene-2,3-diyl)bis(phosphonate) (9m).

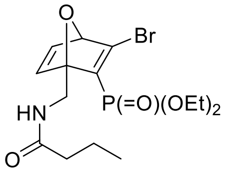

9l

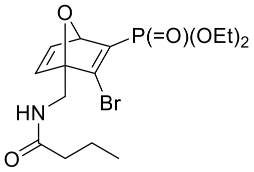

9m

General procedure was followed starting from **S4** (555 mg, 3.32 mmol). Purification (EtOAc → EtOAc:MeOH, 20:1) afforded **9l** and **9m** in a 5:1 ratio (127 mg, 0.311 mmol, 37%, yellowish oil). A small fraction was further purified for characterisation purposes. Data for compound **9l**: **^{1}H NMR (300 MHz, $CDCl_3$, 298 K, δ ppm, *J* Hz):** δ 7.15 (dd, 1H, $J_{H,H}$ = 5.3, 1.9), 6.98 (d, 1H, $J_{H,H}$ = 5.3), 6.77 (br. s, 1H), 5.26

(dd, 1H, $J_{H,H}$ = 1.7, 0.6), 4.33 (dd, 1H, $J_{H,H}$ = 14.6, 7.1), 4.37-4.00 (m, 4H), 3.90 (dd, 1H, $J_{H,H}$ = 14.7, 5.2), 2.19 (t, 2H, $J_{H,H}$ = 7.3), 1.73-1.61 (m, 2H), 1.37 (t, 3H, $J_{H,H}$ = 7.0), 1.33 (t, 3H, $J_{H,H}$ = 7.0), 0.94 (t, 3H, $J_{H,H}$ = 7.4). **$^{13}C\{^1H\}$ NMR (75 MHz, $CDCl_3$, 298 K, δ ppm, *J* Hz):** δ 173.2, 154.4 (d, 1C, $J_{C,P}$ = 6.4), 144.9 (d, 1C, $J_{C,P}$ = 1.1), 142.2 (d, 1C, $J_{C,P}$ = 2.1), 139.3 (d, 1C, $J_{C,P}$ = 207.0), 98.6 (d, 1C, $J_{C,P}$ = 14.9), 89.1 (d, 1C, $J_{C,P}$ = 12.8), 62.8 (d, 1C, $J_{C,P}$ = 5.6), 62.5 (d, 1C, $J_{C,P}$ = 5.8), 38.6, 19.1, 16.3 (m, 2C), 13.8. Data for compound **9m**: **^1H NMR (300 MHz, $CDCl_3$, 298 K, δ ppm, *J* Hz):** δ 7.18 (dd, 1H, $J_{H,H}$ = 5.3, 1.6), 6.95 (d, 1H, $J_{H,H}$ = 5.3), 5.69-5.64 (m, 2H), 4.26 (ddd, 1H, $J_{H,H}$ = 14.8, 6.5, 1.2), 4.17-4.00 (m, 4H), 3.80 (dd, 1H, *J* = 14.8, 4.8), 2.17 (t, 2H, $J_{H,H}$ = 7.3), 1.71-1.59 (m, 2H), 1.33 (td, 6H, $J_{H,H}$ = 7.1, 3.7), 0.93 (t, 3H, $J_{H,H}$ = 7.4). **$^{13}C\{^1H\}$ NMR (75 MHz, $CDCl_3$, 298 K, δ ppm, *J* Hz):** δ 173.1, 151.4 (d, 1C, $J_{C,P}$ = 6.4), 146.1 (d, 1C, $J_{C,P}$ = 1.5), 142.6 (d, 1C, $J_{C,P}$ = 210.8), 141.5 (d, 1C, $J_{C,P}$ = 2.1), 97.1 (d, 1C, $J_{C,P}$ = 12.4), 86.0 (d, 1C, $J_{C,P}$ = 13.8), 62.5-62.4 (m, 2C), 38.5, 38.8, 19.1, 16.3 (d, 2C, $J_{C,P}$ = 6.4), 13.7. **HRESIMS m/z:** found, 408.0570; calcd. for $C_{15}H_{24}O_5N^{79}BrP$ $[M+H]^+$: 408.0570.

***N*-(2-(((*rac*)-3-tosyl-7-oxabicyclo[2.2.1]hepta-2,5-dien-2-yl)thio)ethyl)acetamide (12).**

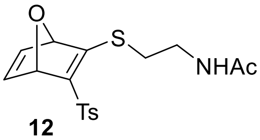

12

To a solution of **9b** (380 mg, 1.04 mmol) in THF (2 mL) and H_2O (1 mL), a solution of *N*-acetylcysteamine (104 mg, 0.87 mmol) in THF (1 mL) was added. Then, K_2CO_3 was added in portions (120 mg, 0. 87 mmol each 30 min). The reaction was vigorously stirred at r.t. for 2 h. Then, the mixture was diluted with DCM and washed twice with water. The organic layer was dried with anhydrous Na_2SO_4, filtered and concentrated under reduced pressure. The crude was purified by column chromatography on silica gel (EtOAc:Cy, 10:1 → EtOAc) to afford **12** (235 mg, 0.64 mmol, 74%, brownish oil). **^1H NMR (300 MHz, $CDCl_3$, 298 K, δ ppm, *J* Hz):** δ 7.72 (ap. d, 2H, $J_{H,H}$ = 8.4), 7.32 (ap. d, 2H, $J_{H,H}$ = 8.0), 6.95 (dd, 1H, $J_{H,H}$ = 1.8, $J_{H,H}$ = 5.2), 6.89 (dd, 1H, $J_{H,H}$ = 1.8, $J_{H,H}$ = 5.2), 6.27 (br. s, 1H, $J_{H,H}$ = 5.7), 5.81-5.80 (m, 1H), 5.52-5.50 (m, 1H), 3.54-3.51 (m, 2H), 3.24-2.98 (m, 2H), 2.43 (s, 3H), 1.96 (s, 3H). **$^{13}C\{^1H\}$ NMR (75 MHz, $CDCl_3$, 298 K, δ ppm):** δ 170.9, 165.8, 144.6, 143.3, 140.7, 139.4, 136.9, 129.9, 127.0, 86.5, 84.9, 40.6, 31.6, 23.0, 21.6. **HRESIMS m/z:** found, 388.0641; calcd. for $C_{17}H_{19}O_4NNaS_2$ $[M+Na]^+$, 388.0647.

***N*-(2-(((*rac*)-7-(propan-2-ylidene)-3-tosylbicyclo[2.2.1]hepta-2,5-dien-2-yl)thio)ethyl)acetamide (14).**

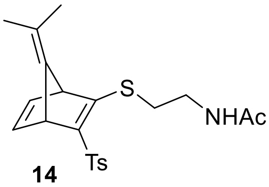

14

To a solution of **9t** (135 mg, 0.369 mmol) in THF (0.8 mL) and H_2O (0.8 mL), a solution of *N*-acetylcysteamine (40 mg, 0.34 mmol) in THF (0.8 mL) was added. Then, K_2CO_3 was added in portions (46 mg, 0.34 mmol each 30 min). The reaction was vigorously stirred at r.t. for 1 h. Then, the mixture was diluted in DCM and washed twice with water and once with brine. The organic layer was dried with anhydrous Na_2SO_4, filtered and concentrated under reduced pressure. The crude was purified by column chromatography on silica gel (EtOAc:Cy, 1:1 → 10:1) to afford **14** (85 mg, 0.21 mmol, 63%, brownish oil). **^1H NMR (300 MHz, $CDCl_3$, 298 K, δ ppm, *J* Hz):** δ 7.70 (ap. d, 2H, $J_{H,H}$ = 8.3), 7.28 (ap. d, 2H, $J_{H,H}$ =8.0), 6.71-6.65 (m, 2H), 6.28 (br. s, 1H), 4.61-4.59 (m, 1H), 4.29-4.27 (m, 1H), 3.52-3.29 (m,

2H), 3.18-2.97 (m, 2H), 2.41 (s, 3H), 1.95 (s, 3H), 1.47 (s, 3H), 1.37 (s, 3H). **^{13}C{^{1}H} NMR (75 MHz, $CDCl_3$, 298 K, δ ppm):** δ 170.6, 163.6, 157.3, 144.0, 142.6, 141.7, 138.8, 137.5, 129.6, 126.9, 100.7, 56.1, 52.9, 40.4, 31.9, 23.1, 21.6, 18.5, 18.3. **HRESIMS m/z:** found, 426.1165; calcd. for $C_{21}H_{25}O_3NNaS_2$ $[M+Na]^+$, 426.1168.

Diethyl((*rac*)-3-((2-acetamidoethyl)thio)-7-oxabicyclo[2.2.1]hepta-2,5-dien-2-yl)phosphonate (15).

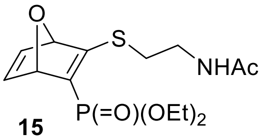

15

To a solution of **9j** (370 mg, 1.20 mmol) in DMF (20 mL), a previously prepared solution of *N*-acetylcysteamine (119 mg, 0.998 mmol) in DMF (10 mL) and sodium phosphate buffer (pH 8, 20 mL) was added. After 4.5 h at r.t., the solvent was removed under reduced pressure. The residue was diluted in EtOAc and washed once with H_2O and the aqueous layer was extracted twice with EtOAc. The combined organic layers were dried on anhydrous Na_2SO_4, filtered and concentrated in vacuo. The crude was purified by column chromatography on silica gel (EtOAc → EtOAc:MeOH, 15:1) to afford **15** (199 mg, 0.57 mmol, 57%, colourless oil). **^{1}H NMR (300 MHz, CD_3OD, 298 K, δ ppm, *J* Hz):** δ 7.20 (dd, 1H, $J_{H,H}$ = 5.4, 1.9), 7.15 (dd, 1H, $J_{H,H}$ =5.4, 1.7), 5.88 (dd, 1H, $J_{H,H}$ =3.2, 1.5), 5.67 (dd, 1H, $J_{H,H}$ =2.7, 1.3), 4.09-3.94 (m, 4H), 3.39 (t, 2H, $J_{H,H}$ = 6.9), 3.29-3.19 (m, 1H), 3.15-3.02 (m, 1H), 1.95 (s, 3H), 1.35-1.28 (m, 6H). **^{13}C{^{1}H} NMR (75 MHz, CD_3OD, 298 K, δ ppm, *J* Hz):** δ 174.0 (d, 1C, $J_{C,P}$ = 12.7), 173.6, 145.2 (d, 1C, $J_{C,P}$ = 1.7), 141.8 (d, 1C, $J_{C,P}$ = 2.1), 129.0 (d, 1C, $J_{C,P}$ = 216.8), 87.6 (d, 1H, $J_{C,P}$ = 16.3), 87.0 (d, 1C, $J_{C,P}$ = 14.5), 63.4 (d, 2C, $J_{C,P}$ = 5.5), 41.8, 31.9, 22.5, 16.6 (d, 2C, $J_{C,P}$ = 6.5). **HRESIMS m/z:** found, 370.0849; calcd. for $C_{14}H_{22}O_5NNaPS$ $[M+Na]^+$, 370.0844.

Diethyl ((*rac*)-3-((2-acetamidoethyl)thio)-1,4-dimethyl-7-oxabicyclo[2.2.1]hepta-2,5-dien-2-yl)phosphonate (16).

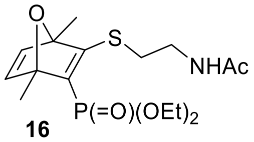

16

To a solution of **9k** (270 mg, 0.829 mmol) in DMF (12 mL), a previously prepared solution of *N*-acetylcysteamine (79 mg, 0.66 mmol) in DMF (6 mL) and sodium phosphate buffer (pH 8, 12 mL) was added. After 5 h at r.t., the solvent was removed under reduced pressure. The residue was diluted in EtOAc and washed once with H_2O and the aqueous layer was extracted twice with EtOAc. The combined organic layers were dried on anhydrous Na_2SO_4, filtered and concentrated in vacuo. The crude was purified by column chromatography on silica gel (EtOAc → EtOAc:MeOH, 15:1) to afford **16** (99 mg, 0.26 mmol, 40%, colourless oil). **^{1}H NMR (300 MHz, CD_3OD, 298 K, δ ppm, *J* Hz):** δ 6.94 (ap. t, 2H, $J_{H,H}$ = 5.4), 4.16-4.06 (m, 4H), 3.41-3.30 (m, 2H), 3.26-3.19 (m, 2H), 1.98 (s, 3H), 1.84 (s, 3H), 1.75 (d, 3H, $J_{H,H}$ = 1.6), 1.41-1.35 (m, 6H). **^{13}C{^{1}H} NMR (75 MHz, CD_3OD, 298 K, δ ppm, *J* Hz):** δ 174.7 (d, 1C, $J_{C,P}$ = 12.0), 173.3, 149.0 (d, 1C, $J_{C,P}$ = 1.9), 146.3 (d, 1C, $J_{C,P}$ = 1.9), 139.6 (d, 1C, $J_{C,P}$ = 210.1), 96.3 (d, 1C, $J_{C,P}$ = 14.4), 94.9 (d, 1C, $J_{C,P}$ = 15.8), 63.5-63.3 (m, 2C), 40.3, 33.8, 22.6, 17.5, 16.7-16.6 (m, 2C), 16.2. **HRESIMS m/z:** found, 398.1162; calcd. for $C_{16}H_{26}O_5NNaPS$ $[M+Na]^+$, 398.1151.

(*rac*)-5,5-Dimethoxy-6-tosyl-7-oxabicyclo[2.2.1]hept-2-ene (25).

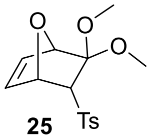

25

To a solution of **9b** (50 mg, 0.15 mmol) in 1 mL of anhydrous THF and 1 mL of anhydrous MeOH under Ar atmosphere, DBU (20 µL, 0.14 mmol) was added. After 1h at r.t. the mixture was concentrated in vacuo. Purification by column chromatography on silica gel (EtOAc:Cy, 1:3) afforded **25** (39 mg, 0.13 mmol, 90%, yellowish oil). **^{1}H NMR (300 MHz, $CDCl_3$, 298 K, δ ppm, *J* Hz):** δ 7.80-7.76 (m, 2H), 7.36-7.32 (m, 2H), 6.75 (dd, 1H, $J_{H,H}$ = 5.9, 1.6), 6.58 (dd, 1H, $J_{H,H}$ = 5.9, 1.8), 4.87-4.84 (m, 1H), 4.82-4.81 (m, 1H), 3.73 (d, 1H, $J_{H,H}$ =4.1), 3.26 (s, 3H), 3.18 (s, 3H), 2.43 (s, 3H). **^{13}C{^{1}H} NMR (75 MHz, $CDCl_3$, 298 K, δ ppm):** δ 145.0, 137.4, 136.2, 132.8, 129.8, 128.7, 110.6, 85.4, 79.7, 70.3, 51.4, 51.3, 21.7. **HRESIMS m/z:** found, 333.0768; calcd. for $C_{15}H_{18}O_5NaS$ $[M+Na]^+$, 333.0767.

Diethyl ((*rac*)-3-methoxy-7-oxabicyclo[2.2.1]hepta-2,5-dien-2-yl)phosphonate (26).

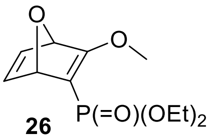

26

To a solution of **9j** (35 mg, 0.11 mmol) in 1 mL of anhydrous THF and 1 mL of anhydrous MeOH under Ar atmosphere, DBU (51 µL, 0.34 mmol) was added. After 1d at r.t. the mixture was concentrated in vacuo. Purification by column chromatography on silica gel (Et_2O:Cy, 5:1 → Et_2O) afforded **27** (17mg, 0.07 mmol, 57%, colourless oil). **^{1}H NMR (300 MHz, $CDCl_3$, 298 K, δ ppm, *J* Hz):** δ 7.27 (dd, 1H, J = 5.4, 1.7), 7.01 (dd, 1H, J = 5.4, 2.0), 5.60 (dd, 1H, J = 3.2, 1.5), 5.03 (dd, 1H, J = 3.6, 1.9), 4.10-3.93 (m, 5H), 1.36-1.23 (m, 6H). **^{13}C{^{1}H} NMR (75 MHz, $CDCl_3$, 298 K, δ ppm, *J* Hz):** δ 187.3 (d, $J_{C,P}$ = 10.1), 148.0 (d, $J_{C,P}$ = 1.5), 139.0 (d, $J_{C,P}$ = 1.9 Hz), 101.7 (d, $J_{C,P}$ = 221.3), 87.1 (d, $J_{C,P}$ = 12.8), 84.3 (d, $J_{C,P}$ = 12.4), 61.9, 61.7 (d, $J_{C,P}$ = 5.2), 61.5 (d, $J_{C,P}$ = 5.3), 16.4 (m, 2C). **HRESIMS m/z:** found, 283.0704; calcd. for $C_{11}H_{17}O_5NaP$ $[M+Na]^+$, 283.0704.

4. ¹H-NMR spectra for known compounds.

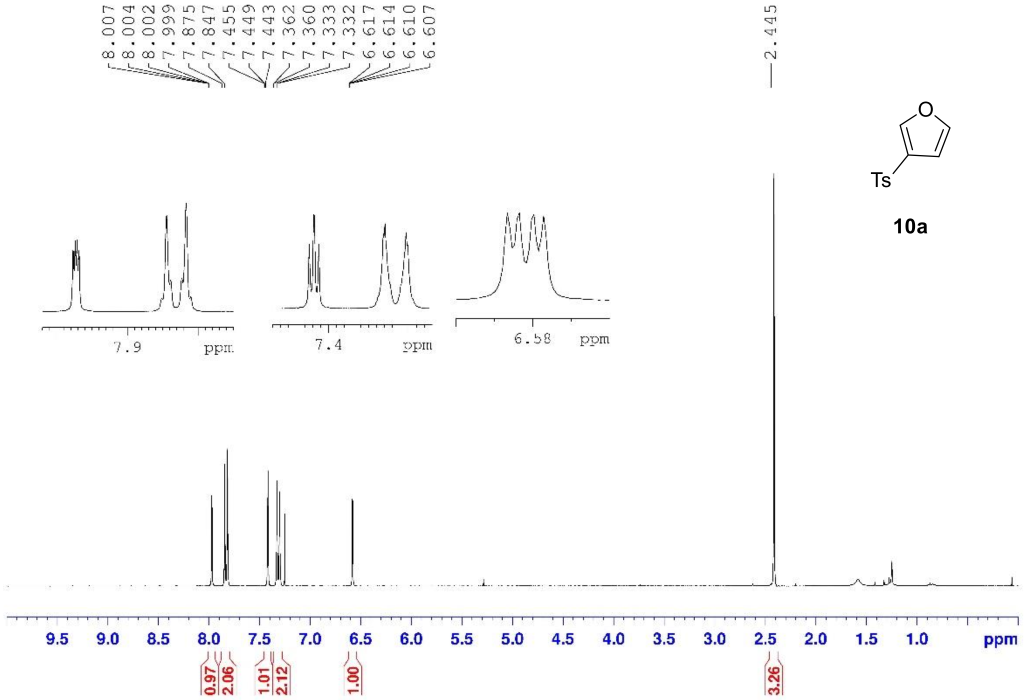

¹H-NMR (300 MHz, $CDCl_3$) of **10a**.

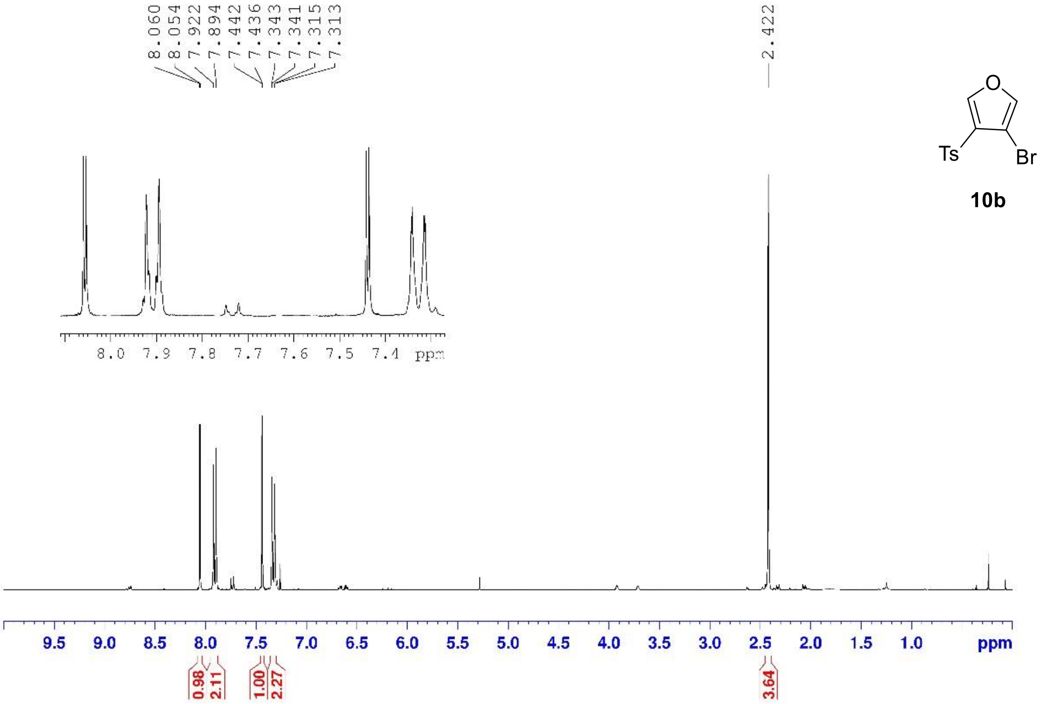

¹H-NMR (300 MHz, $CDCl_3$) of **10b**.

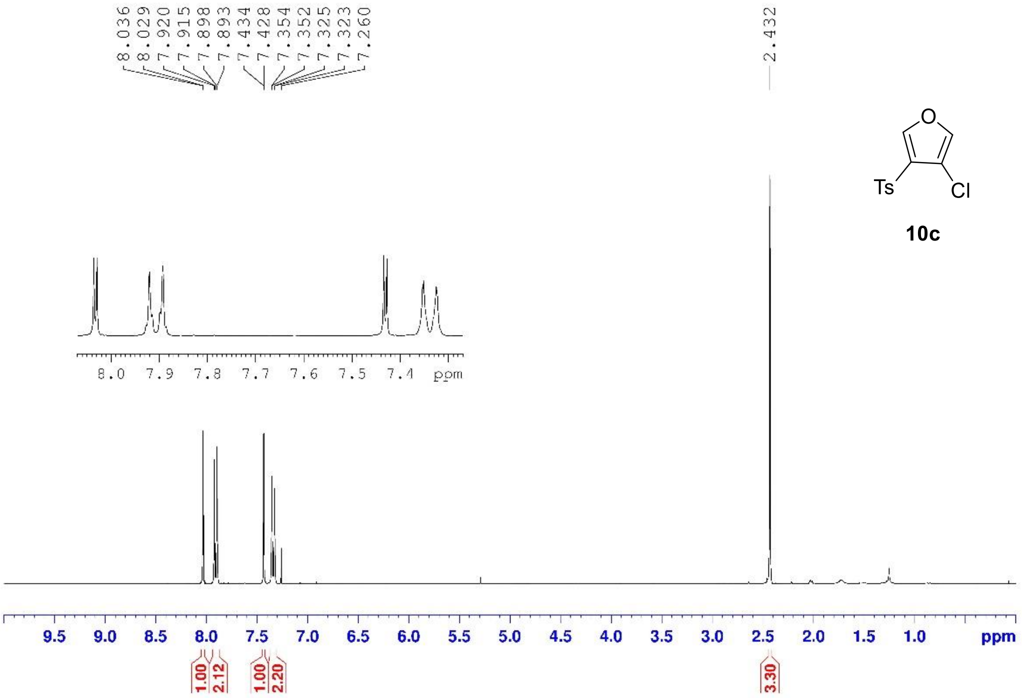

^{1}H-NMR (300 MHz, $CDCl_3$) of **10c**.

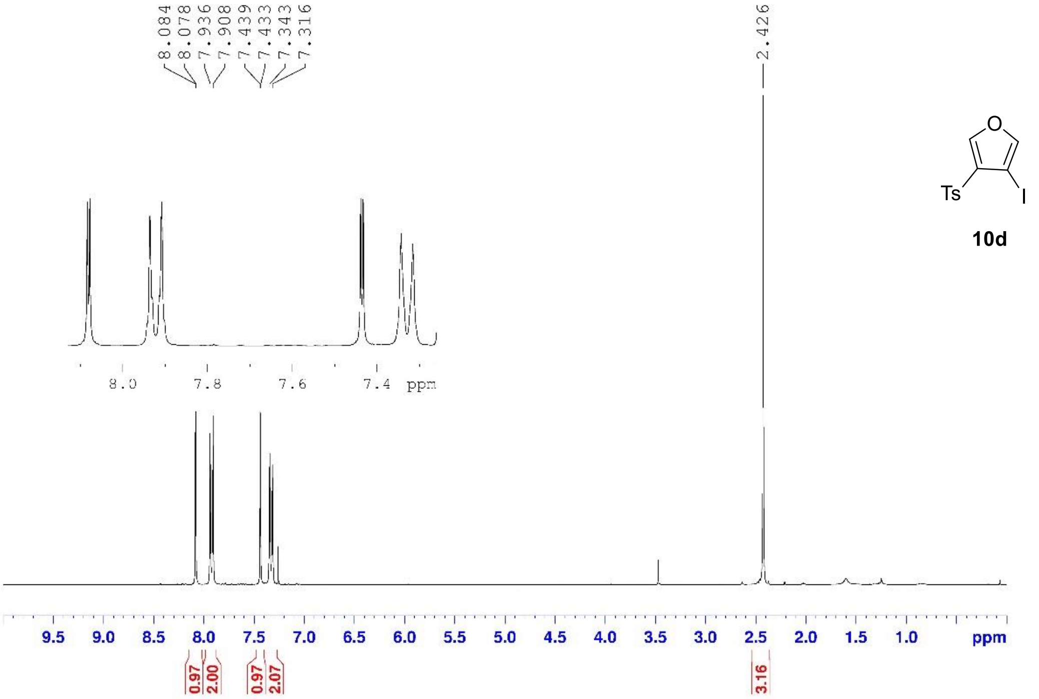

^{1}H-NMR (300 MHz, $CDCl_3$) of **10d**.

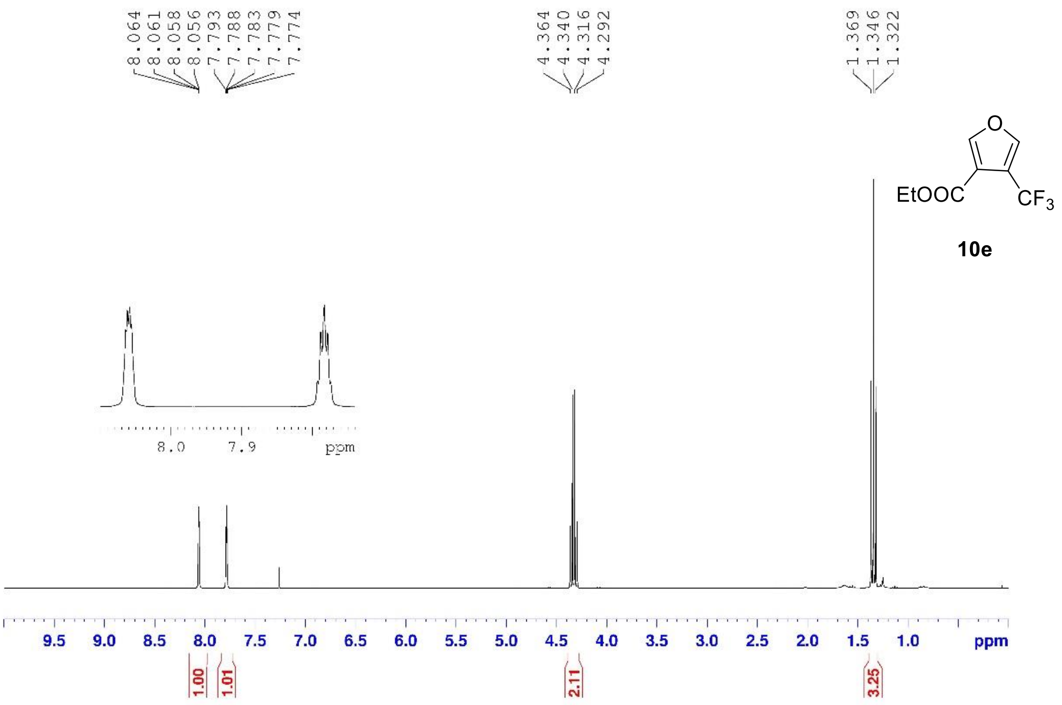

^{1}H-NMR (300 MHz, $CDCl_3$) of **10e**.

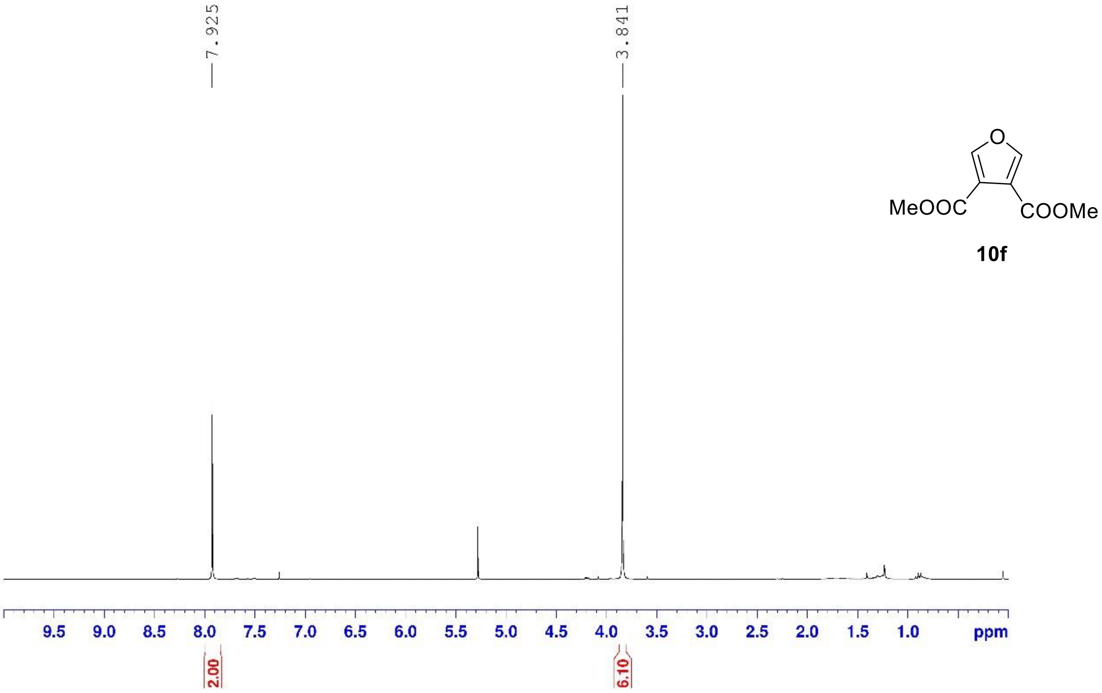

^{1}H-NMR (300 MHz, $CDCl_3$) of **10f**.

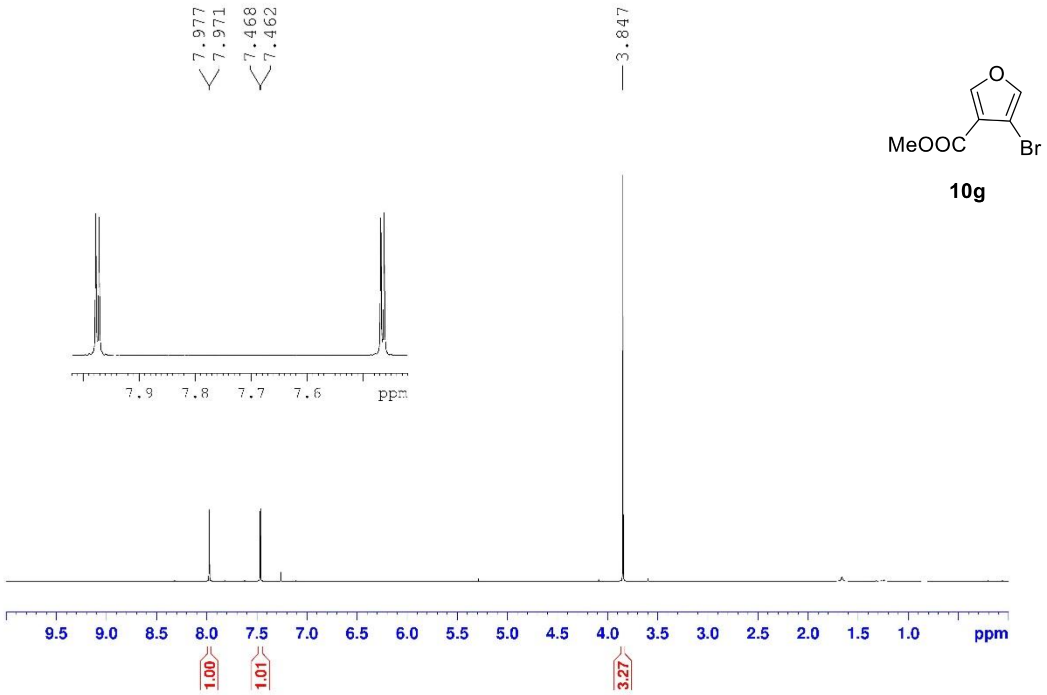

^{1}H-NMR (300 MHz, $CDCl_3$) of **10g**.

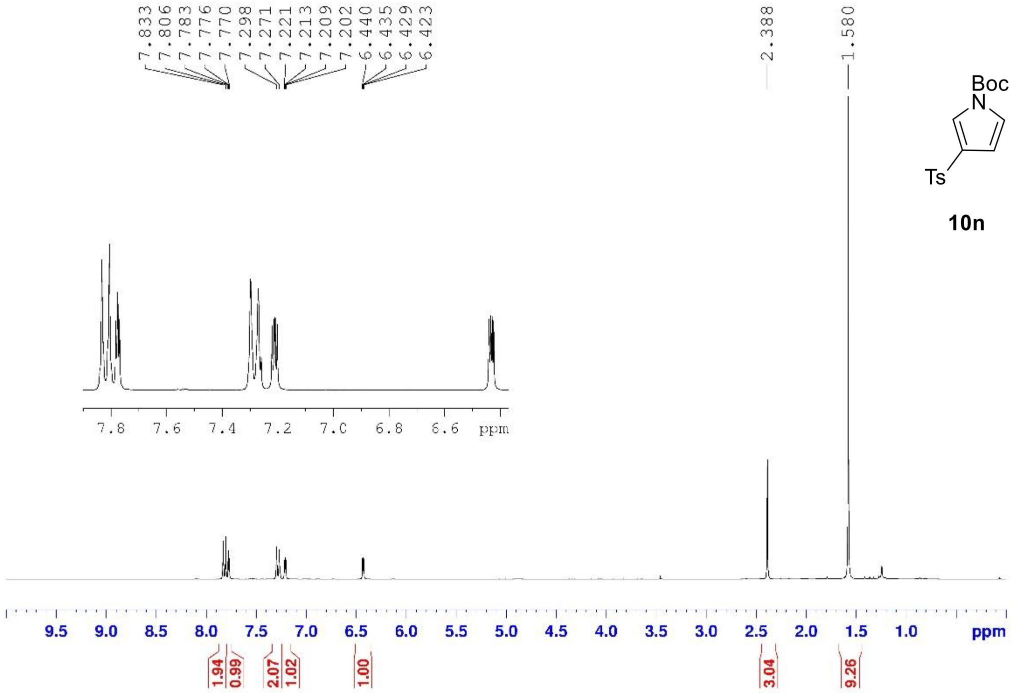

^{1}H-NMR (300 MHz, $CDCl_3$) of **10n**.

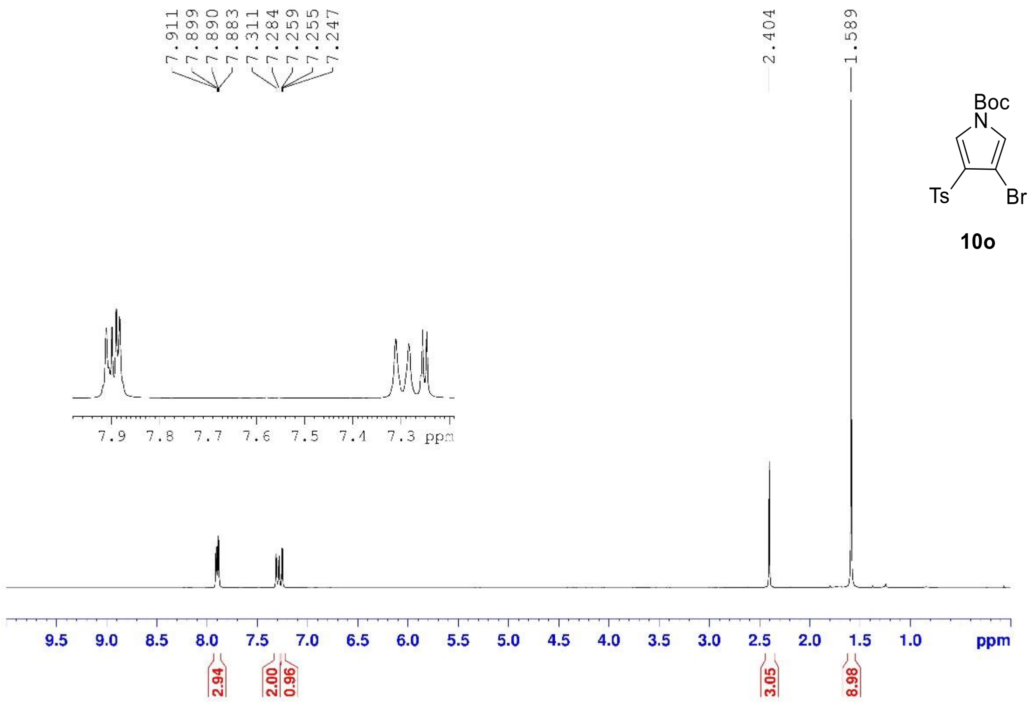

^{1}H-NMR (300 MHz, $CDCl_3$) of **10o**.

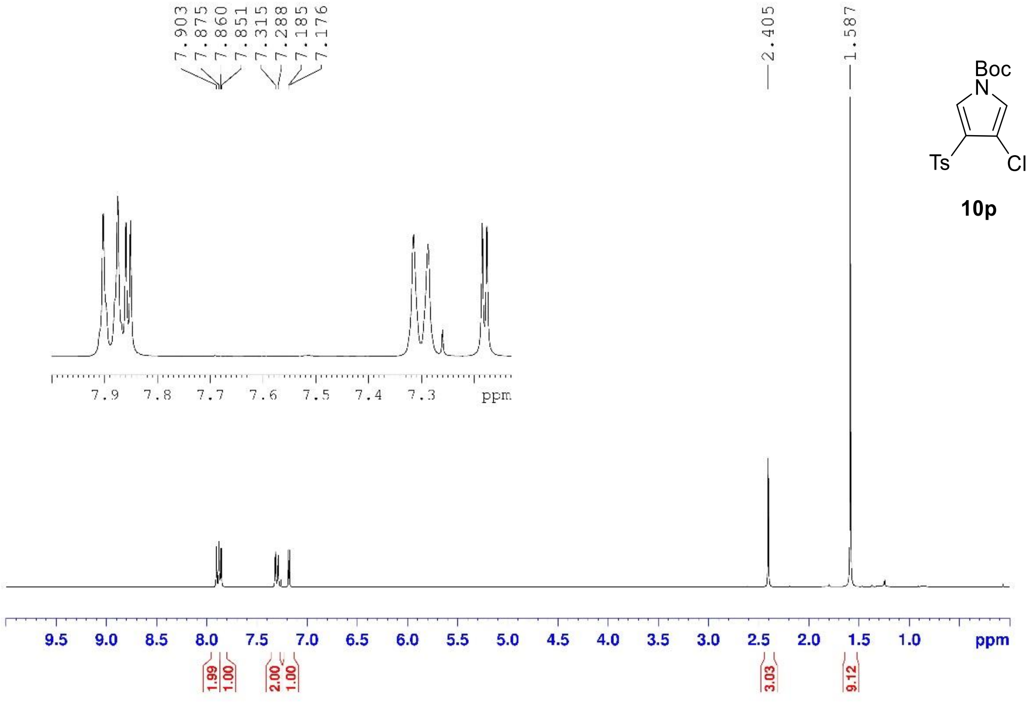

^{1}H-NMR (300 MHz, $CDCl_3$) of **10p**.

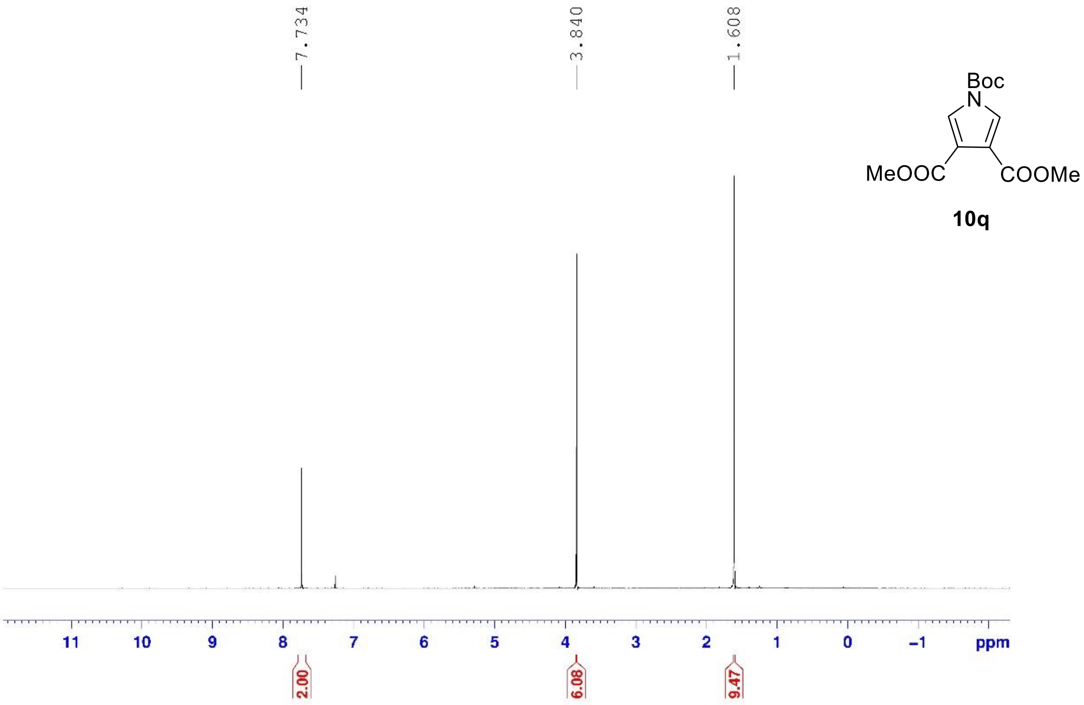

^{1}H-NMR (300 MHz, $CDCl_3$) of **10q**.

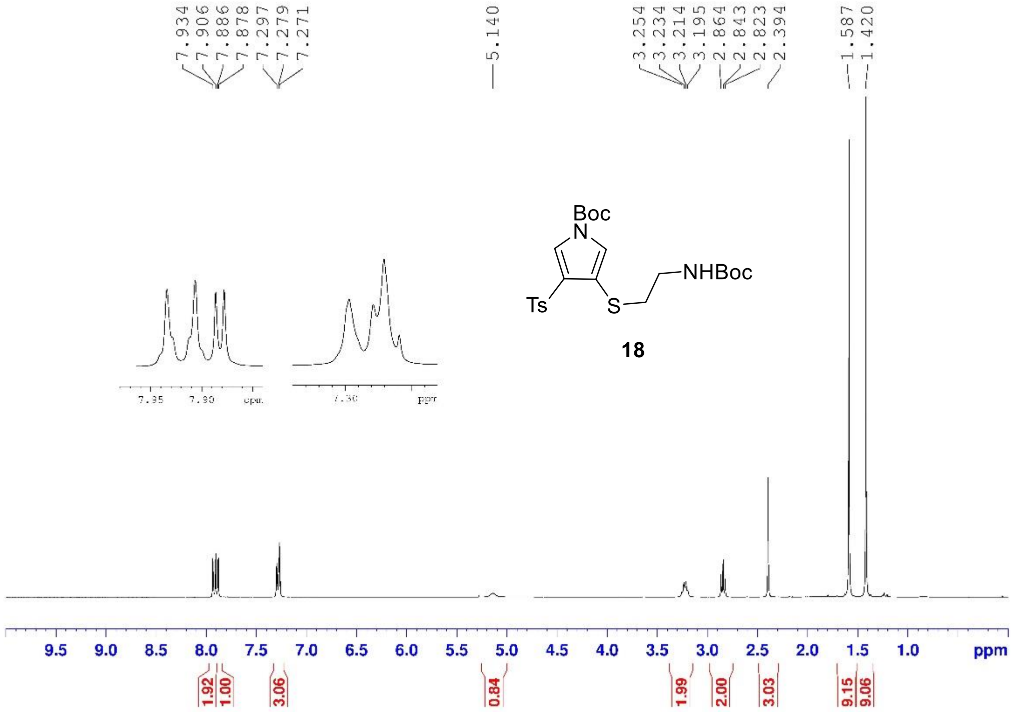

^{1}H-NMR (300 MHz, $CDCl_3$) of **18**.

5. **^{1}H-NMR and ^{13}C-NMR spectra of new compounds.**

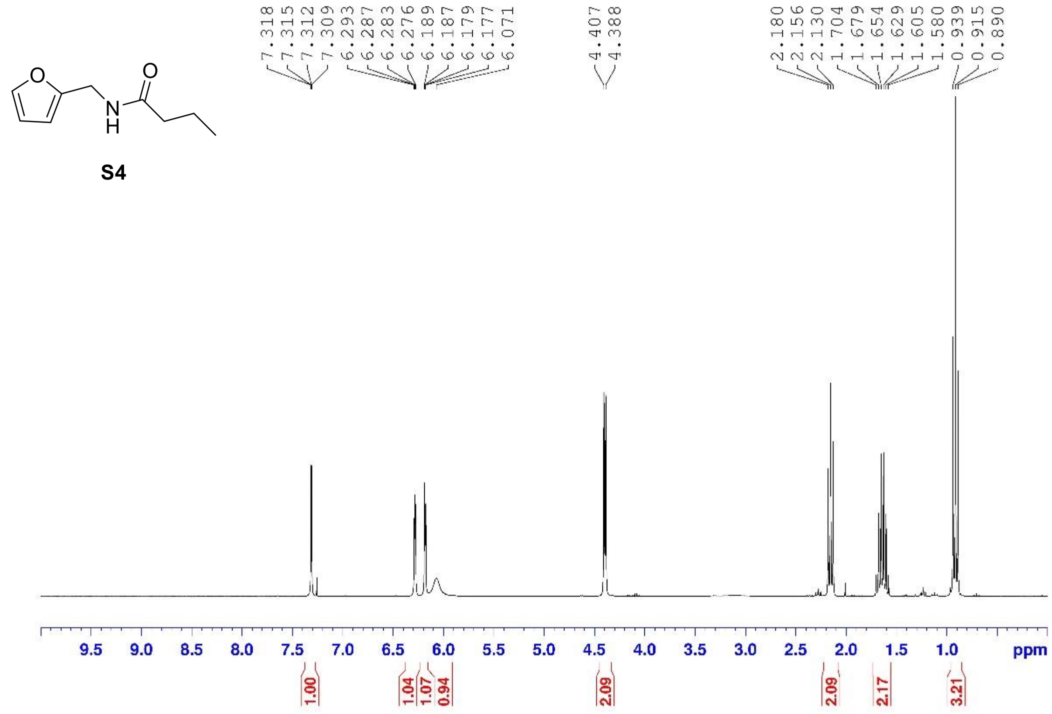

^{1}H-NMR (300 MHz, $CDCl_3$) of **S4**.

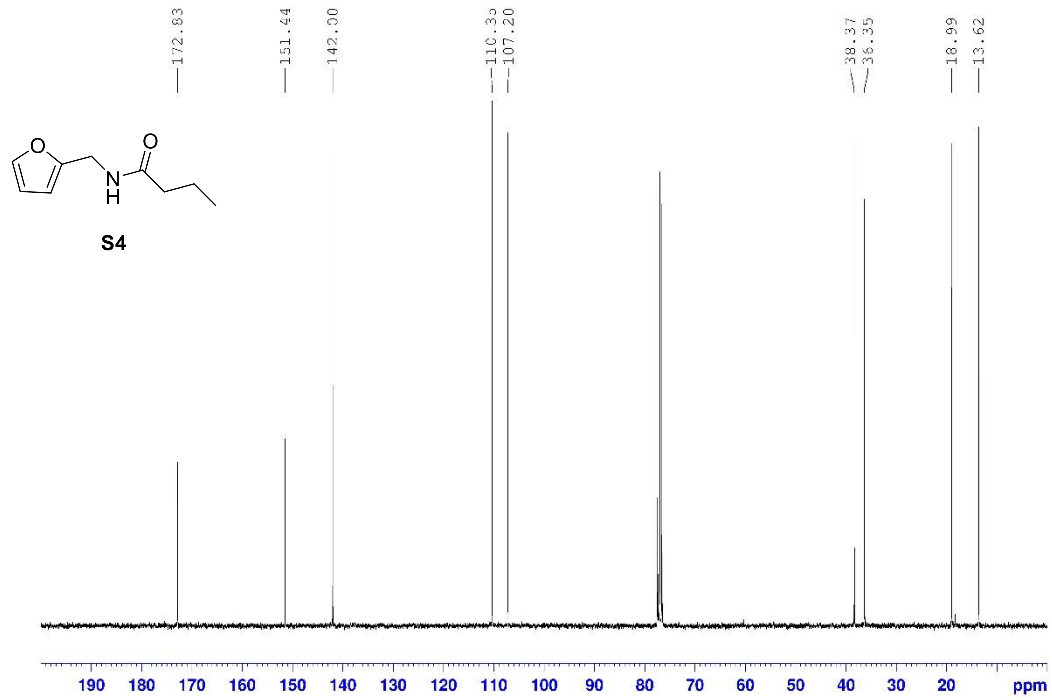

^{13}C {^{1}H} NMR (75.4 MHz, $CDCl_3$) of **S4**.

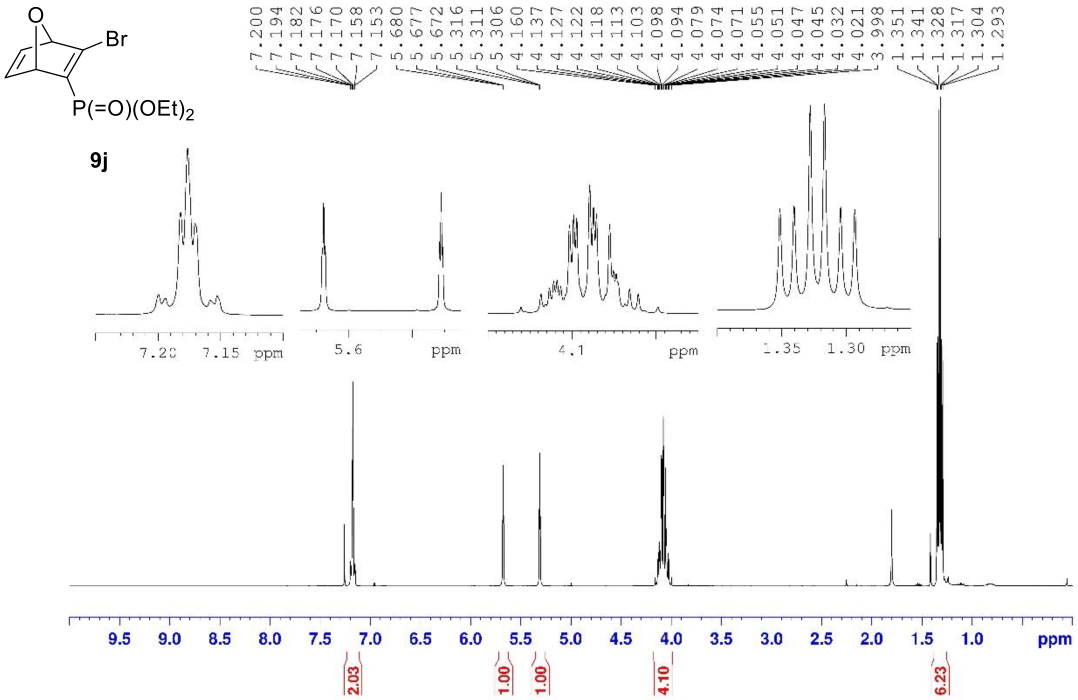

^{1}H-NMR (300 MHz, $CDCl_3$) of **9j**.

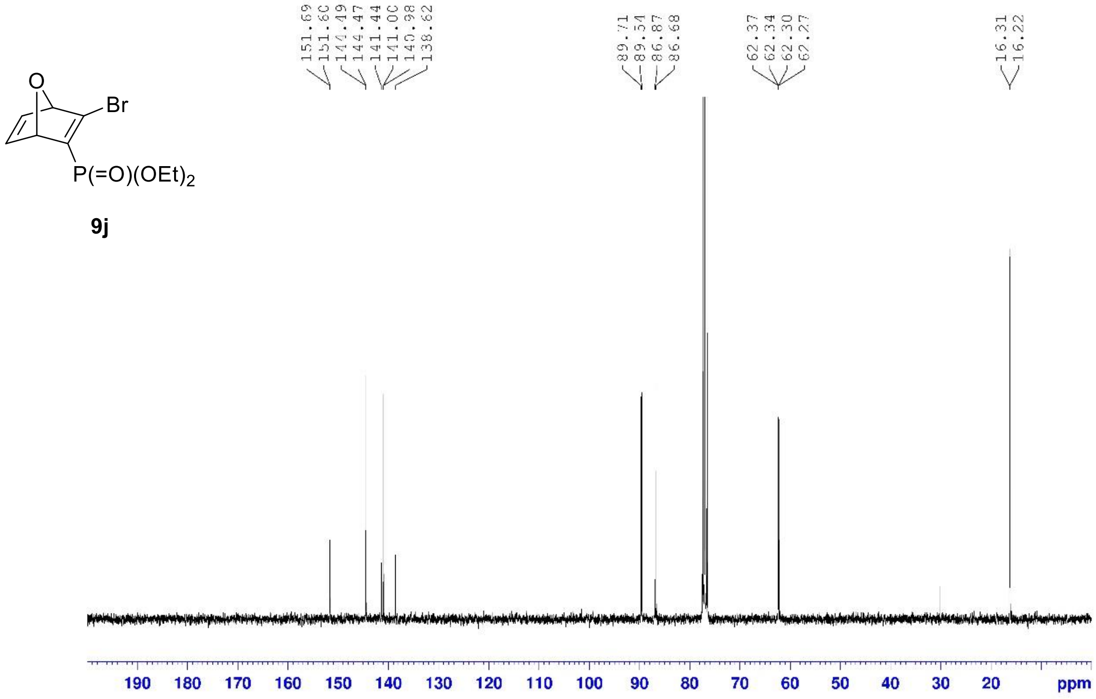

$^{13}C\{^{1}H\}$ NMR (75.4 MHz, $CDCl_3$) of **9j**.

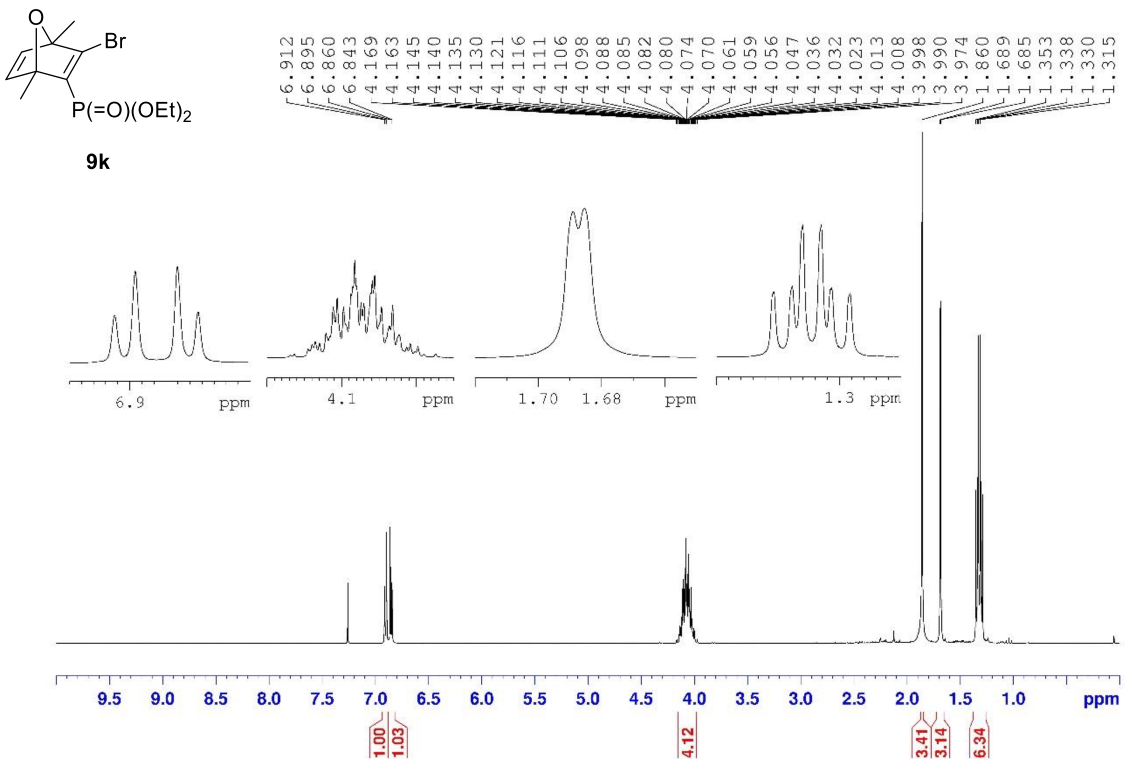

^{1}H-NMR (300 MHz, $CDCl_3$) of **9k**.

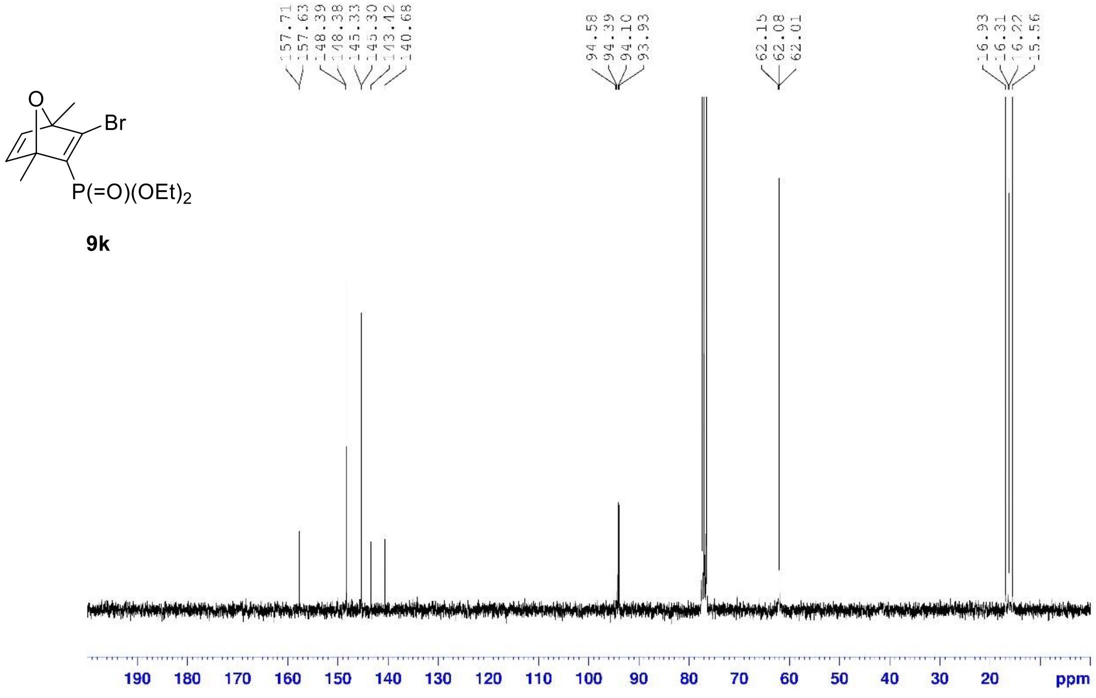

^{13}C{^{1}H } NMR (75.4 MHz, CD_3OD) of **9k**.

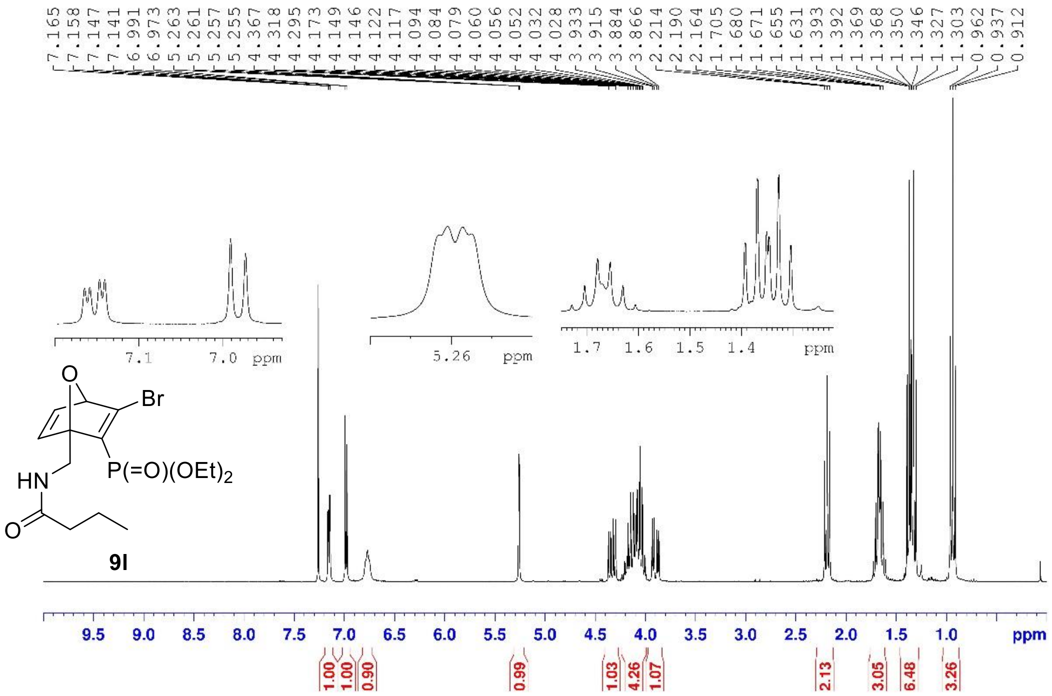

^{1}H-NMR (300 MHz, CDCl$_3$) of **9l**.

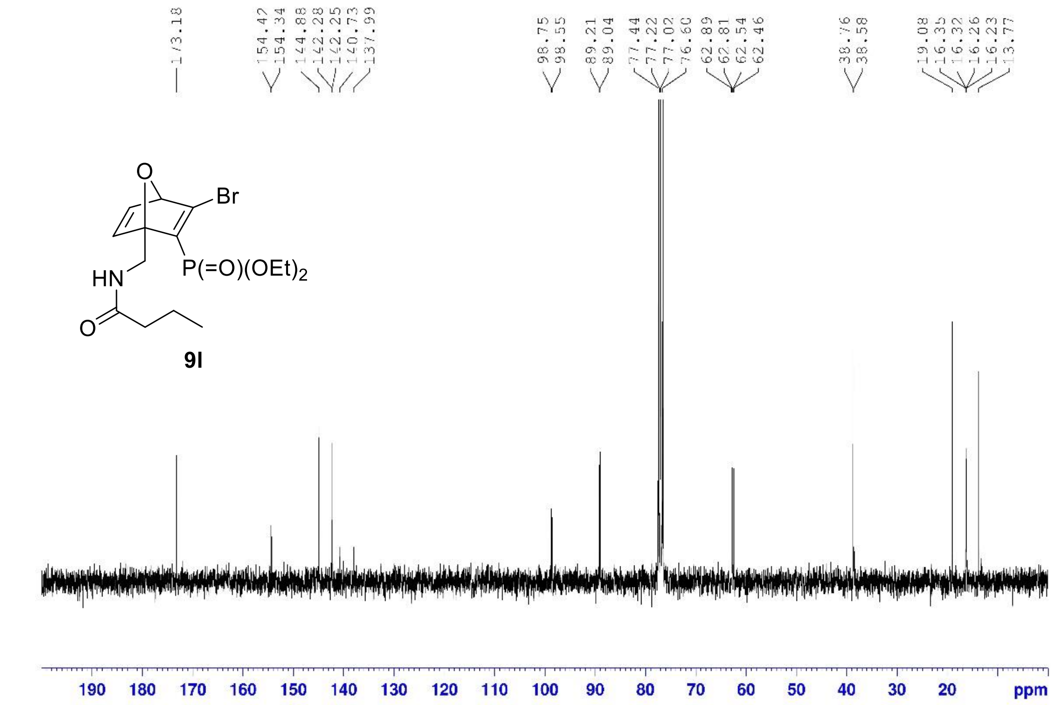

^{13}C{^{1}H } NMR (75.4 MHz, CDCl$_3$) of **9l**.

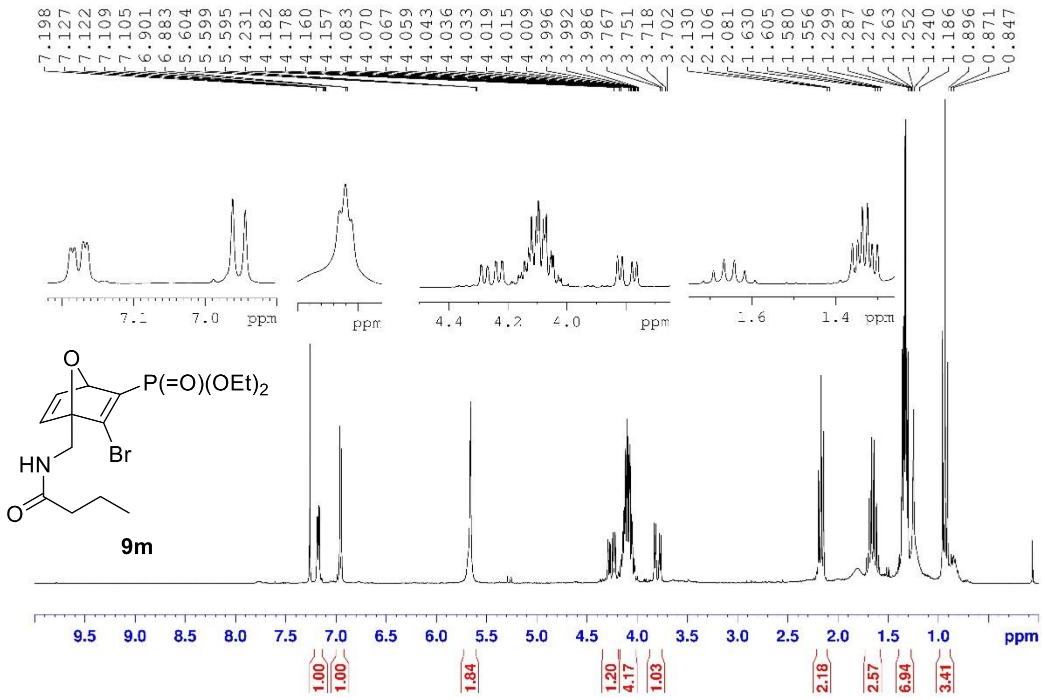

^{1}H-NMR (300 MHz, $CDCl_3$) of **9m**.

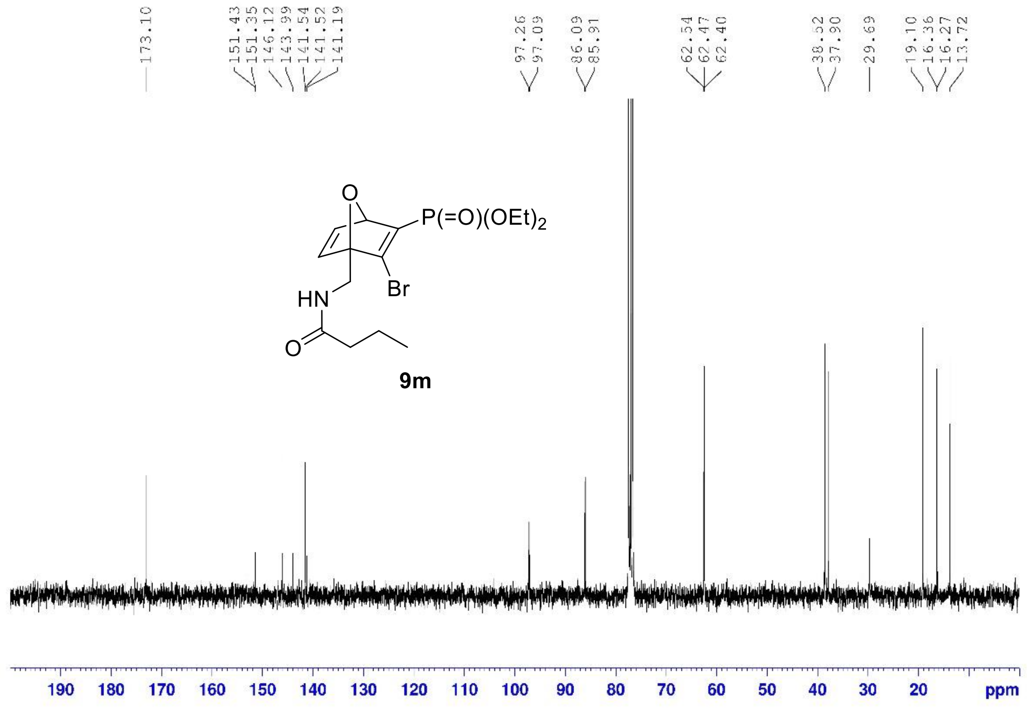

^{13}C{^{1}H } NMR (75.4 MHz, $CDCl_3$) of **9m**.

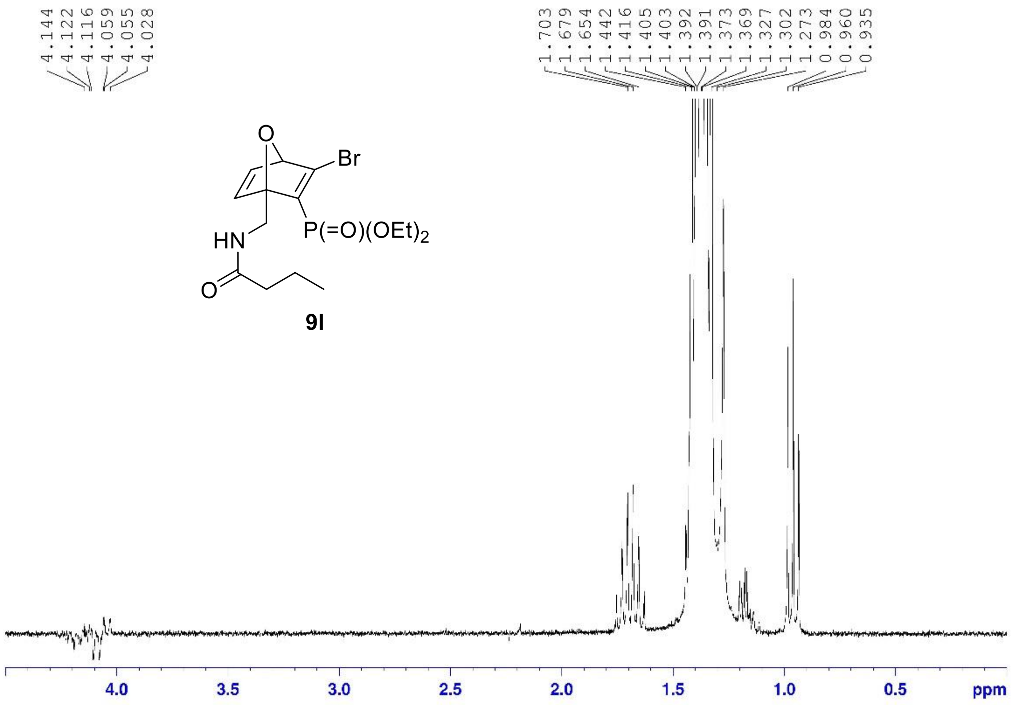

1D-NOE (300 MHz, $CDCl_3$) of **9l**.

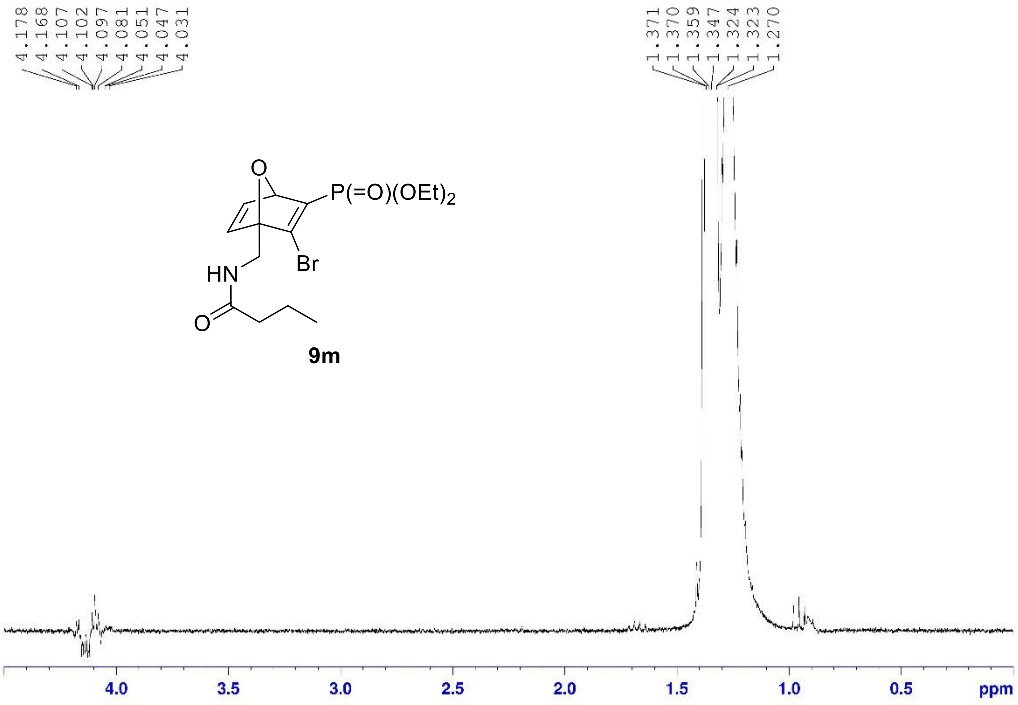

1D-NOE (300 MHz, $CDCl_3$) of **9m**.

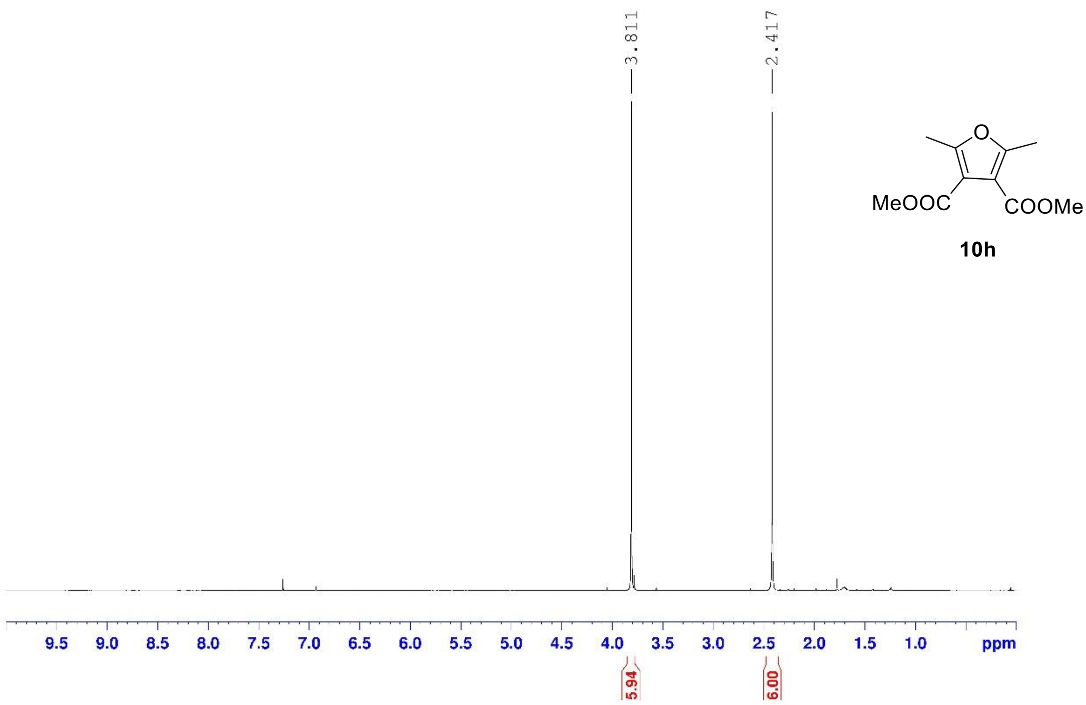

10h

^{1}H-NMR (300 MHz, $CDCl_3$) of **10h**.

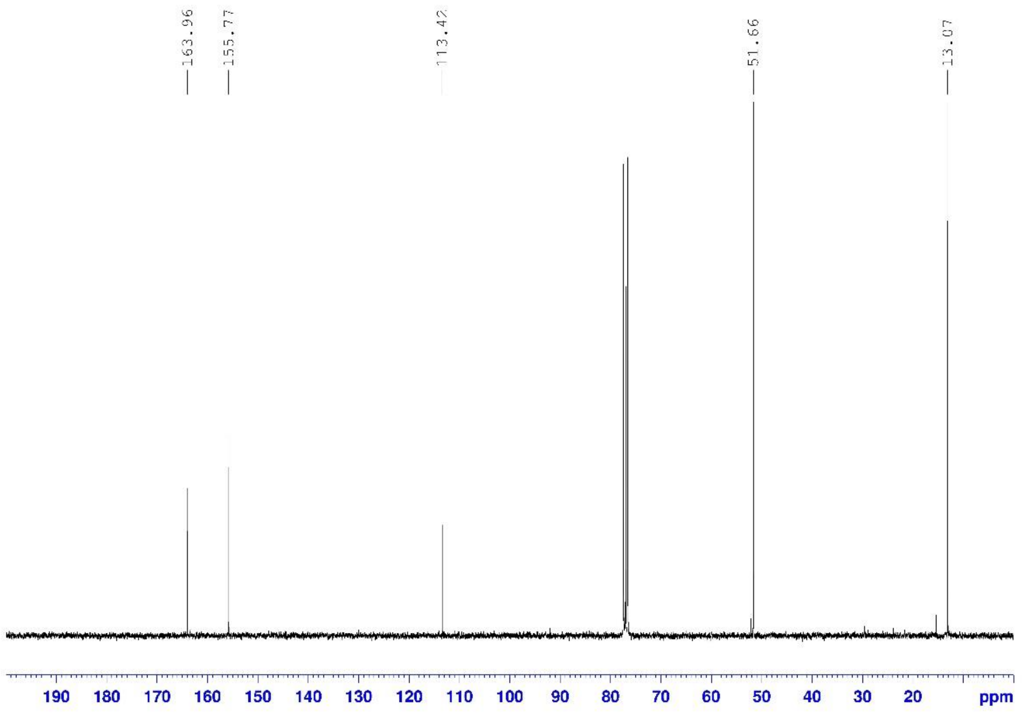

$^{13}C\{^{1}H\}$ NMR (75.4 MHz, $CDCl_3$) of **10h**.

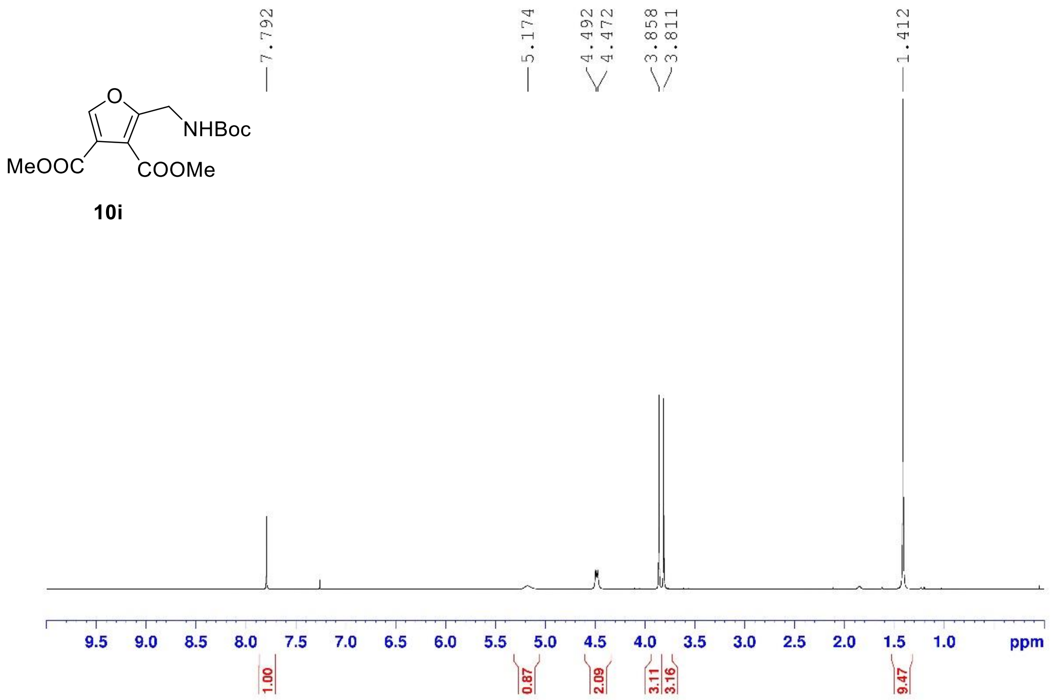

^{1}H-NMR (300 MHz, $CDCl_3$) of **10i**.

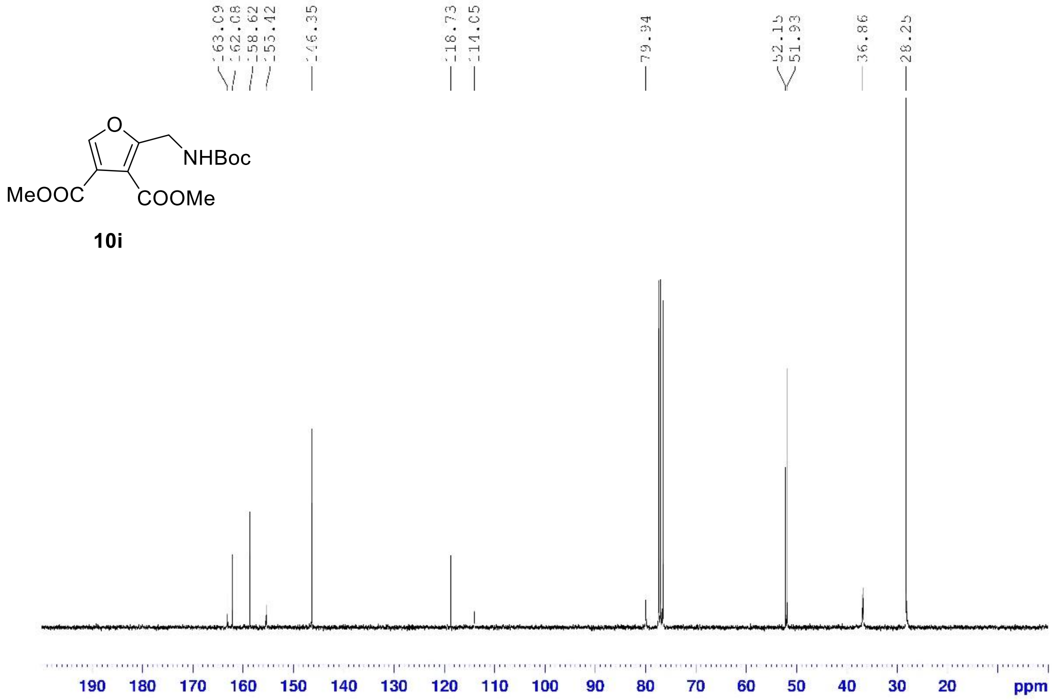

$^{13}C\{^{1}H\}$ NMR (75.4 MHz, $CDCl_3$) of **10i**.

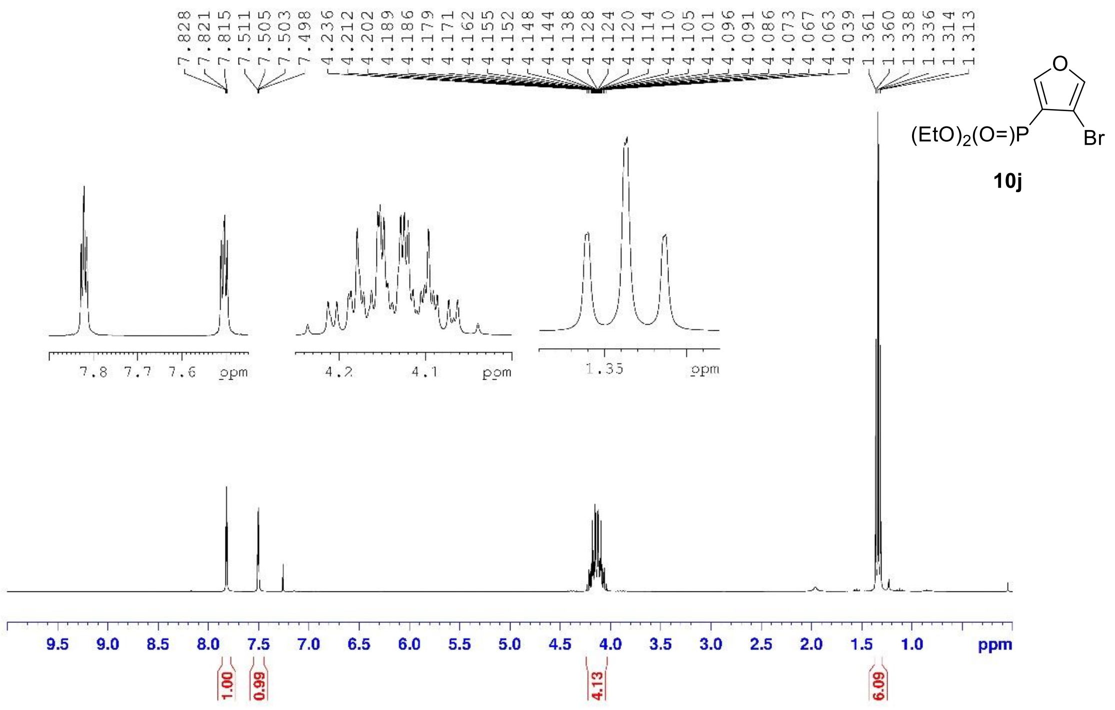

^{1}H-NMR (300 MHz, $CDCl_3$) of **10j**.

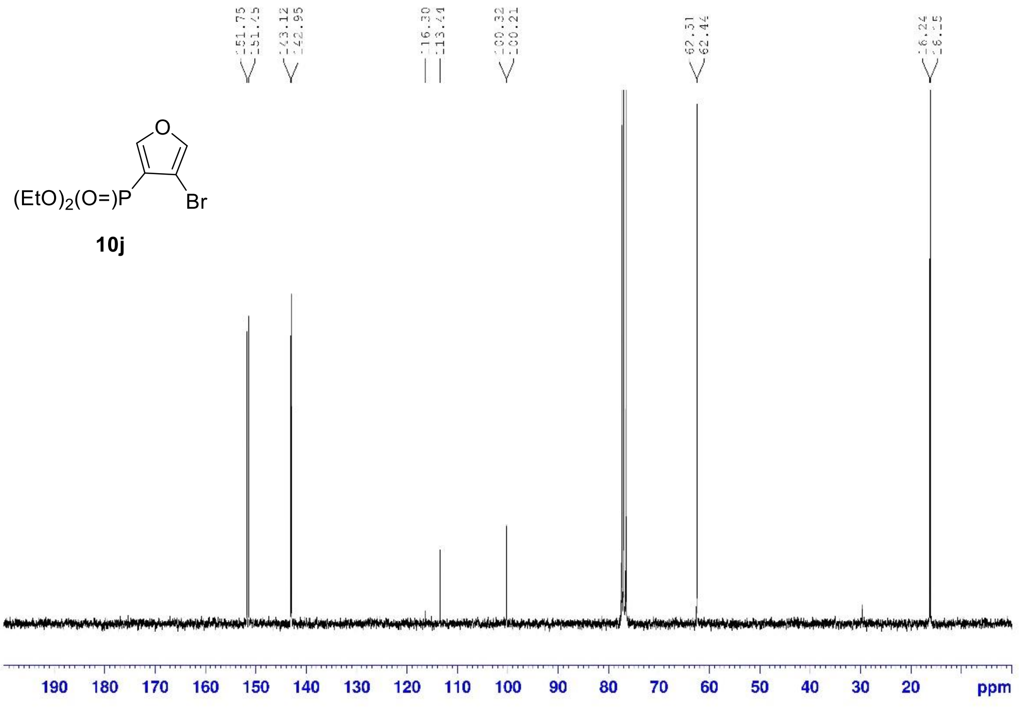

^{13}C{^{1}H } NMR (75.4 MHz, $CDCl_3$) of **10j**.

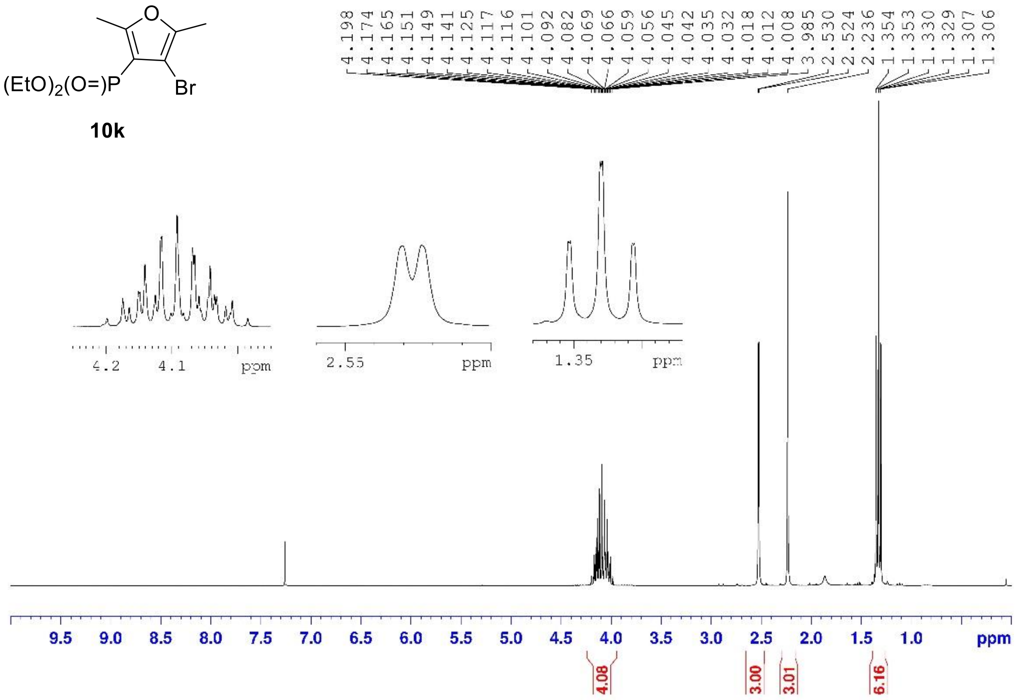

^{1}H-NMR (300 MHz, $CDCl_3$) of **10k**.

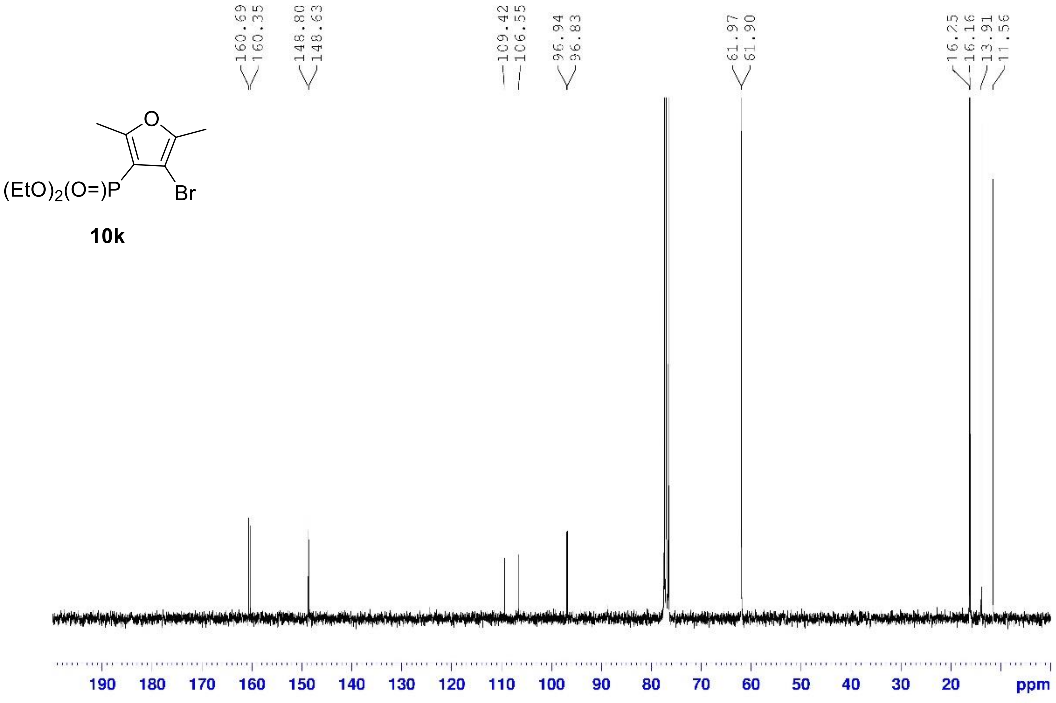

$^{13}C\{^{1}H\}$ NMR (75.4 MHz, $CDCl_3$) of **10k**.

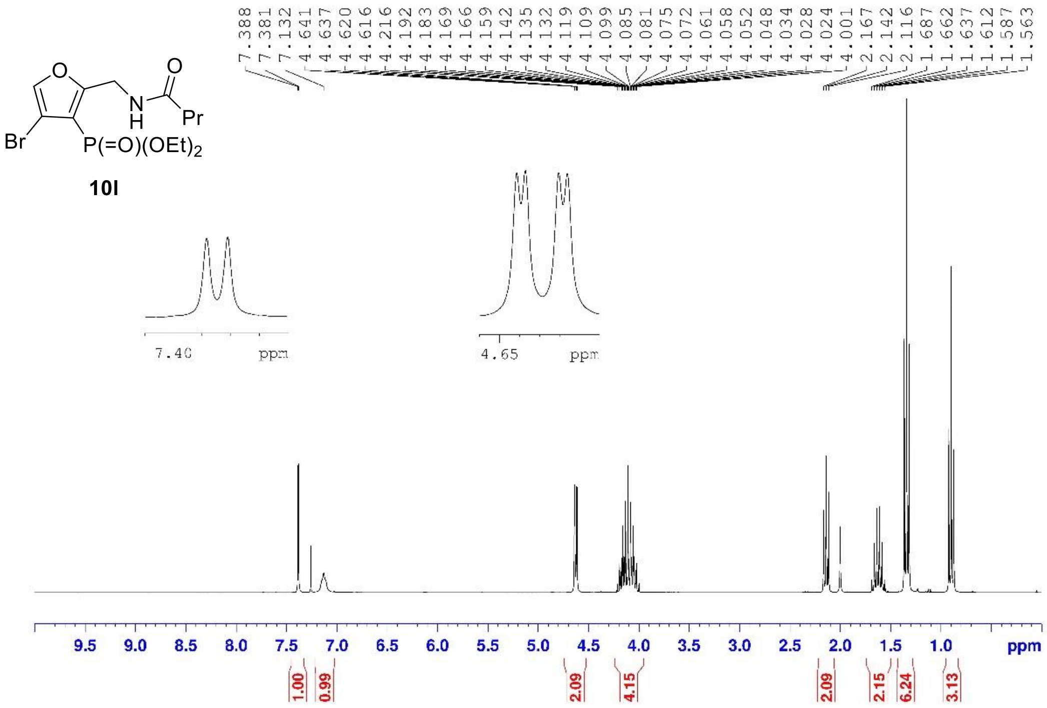

^{1}H-NMR (300 MHz, CDCl$_3$) of **10l**.

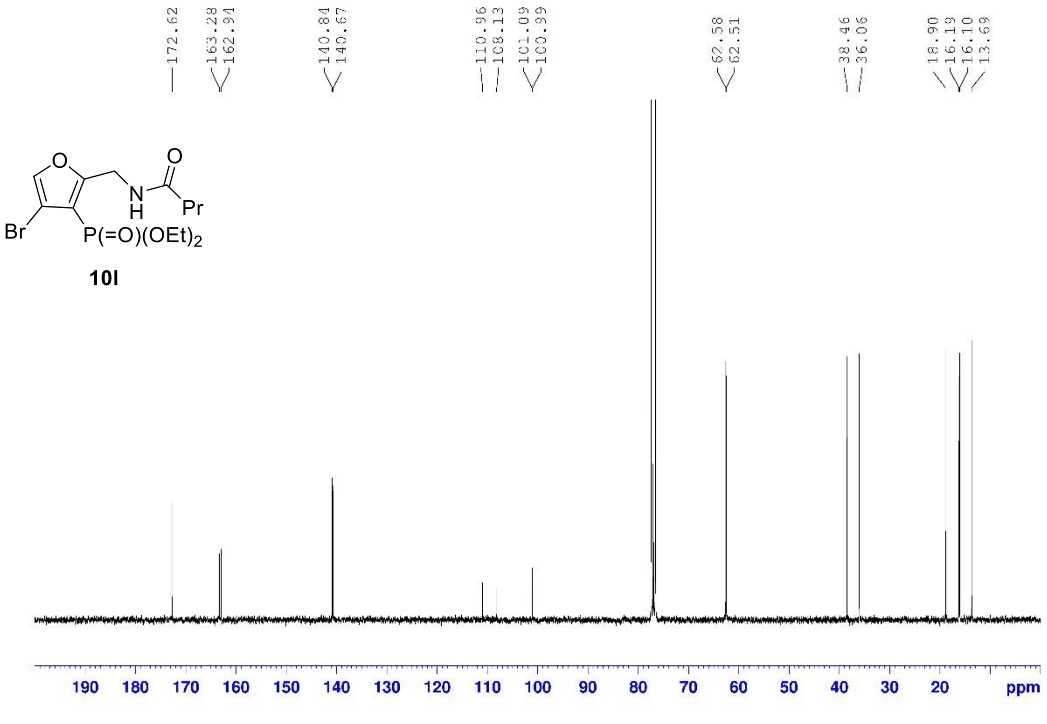

^{13}C{^{1}H } NMR (75.4 MHz, CDCl$_3$) of **10l**.

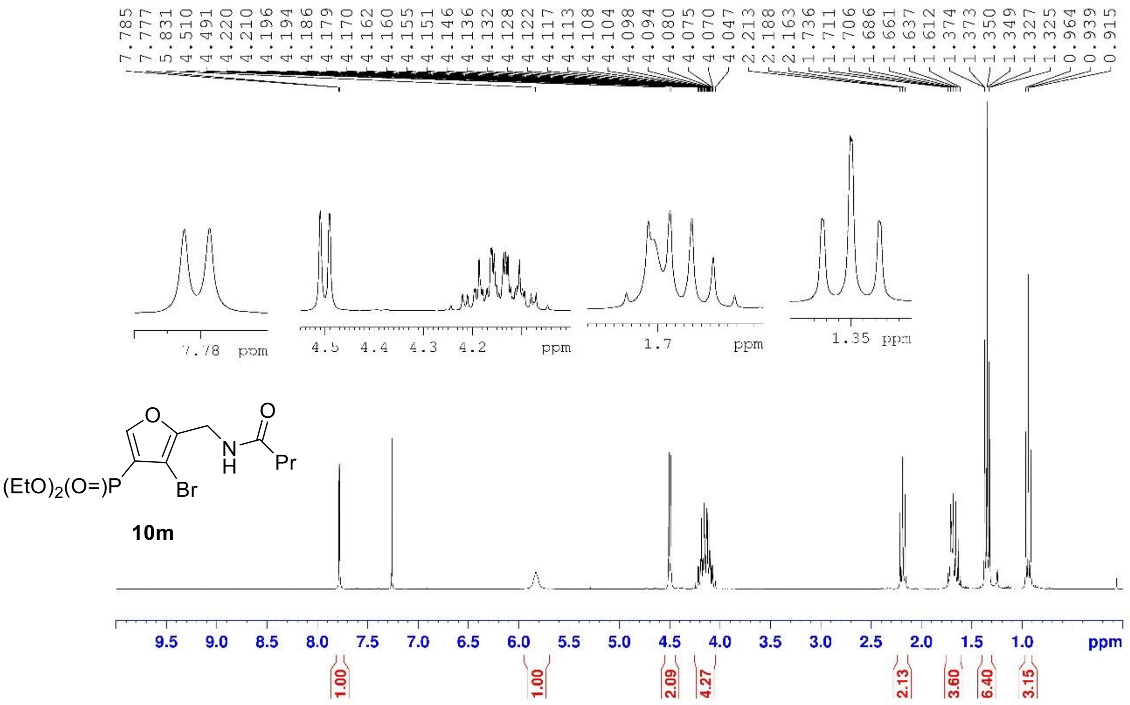

^{1}H-NMR (300 MHz, $CDCl_3$) of **10m**.

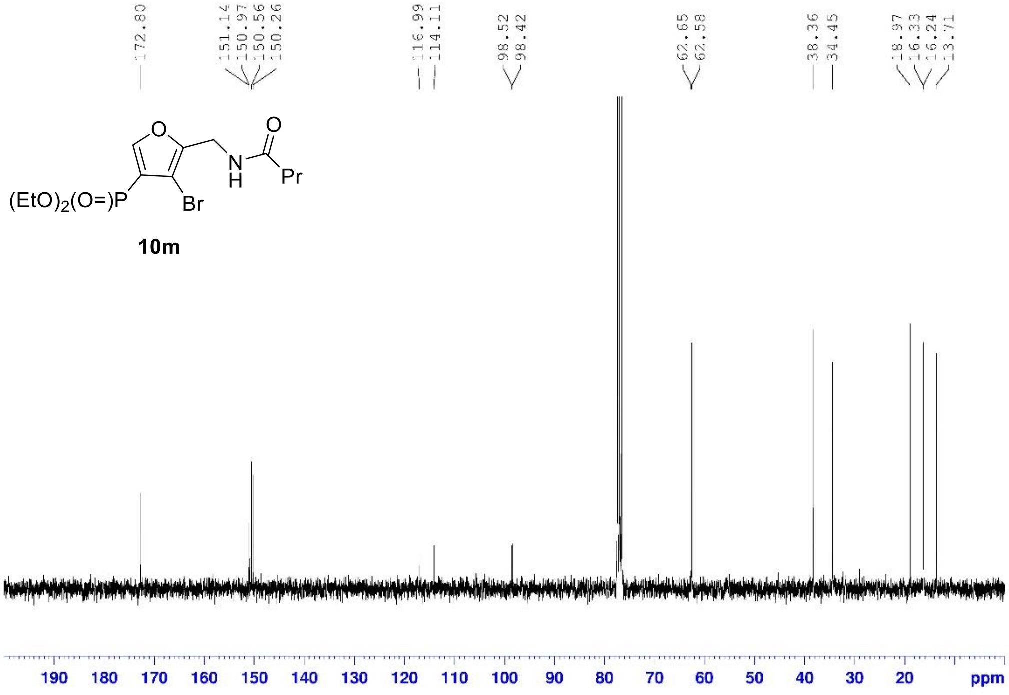

$^{13}C\{^{1}H\}$ NMR (75.4 MHz, $CDCl_3$) of **10m**.

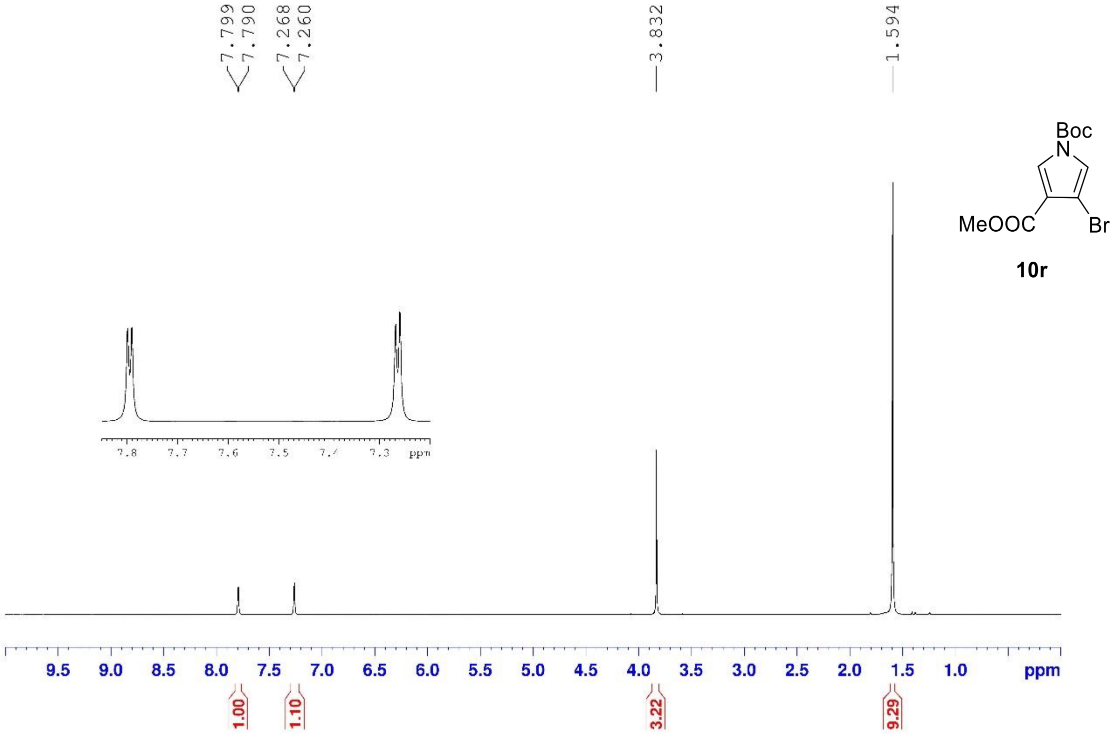

10r

^{1}H-NMR (300 MHz, $CDCl_3$) of **10r**.

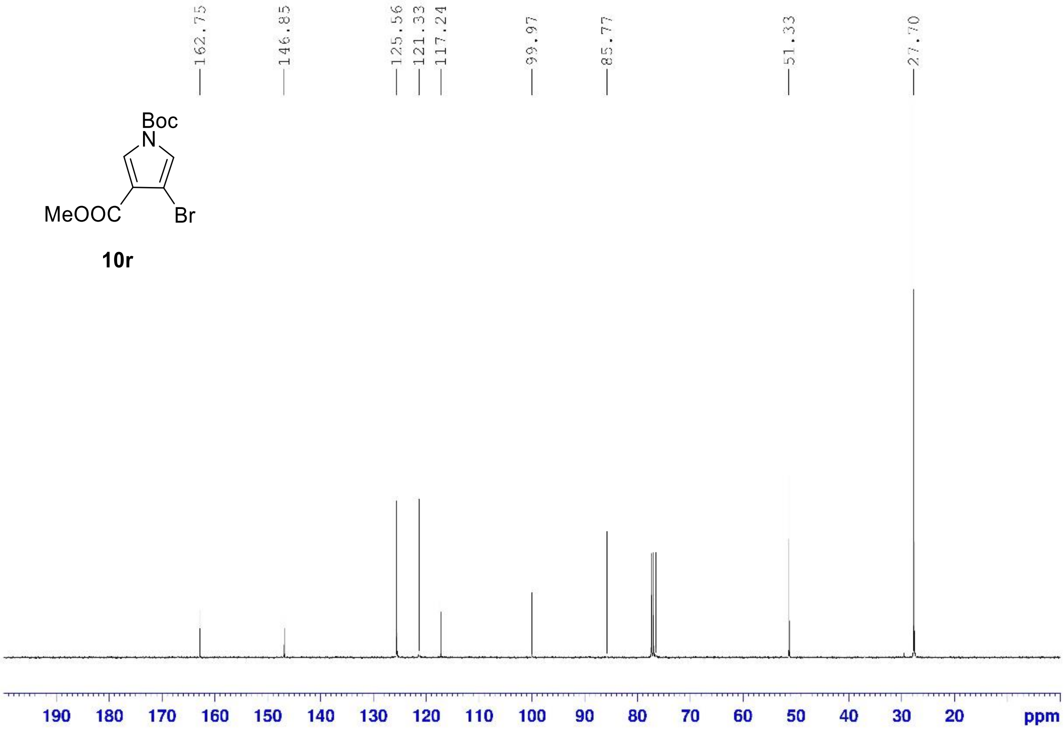

10r

^{13}C{^{1}H } NMR (75.4 MHz, $CDCl_3$) of **10r**.

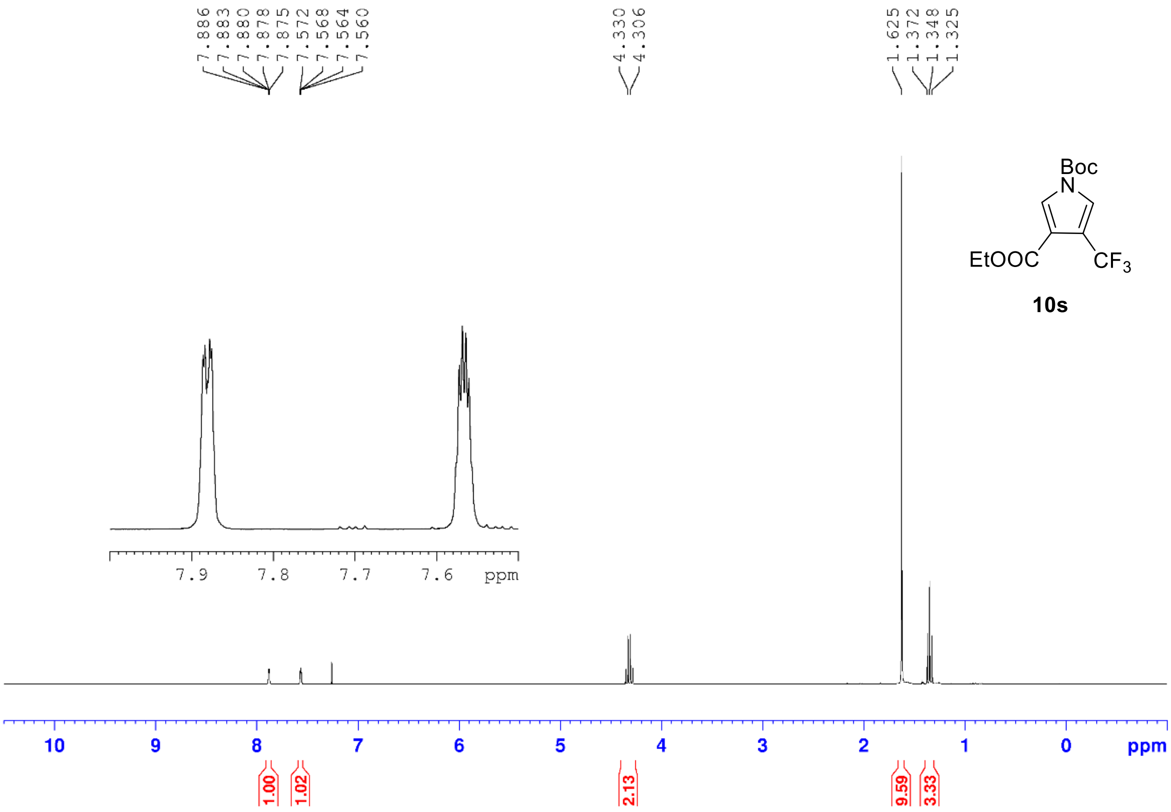

^{1}H-NMR (300 MHz, CDCl$_3$) of **10s**.

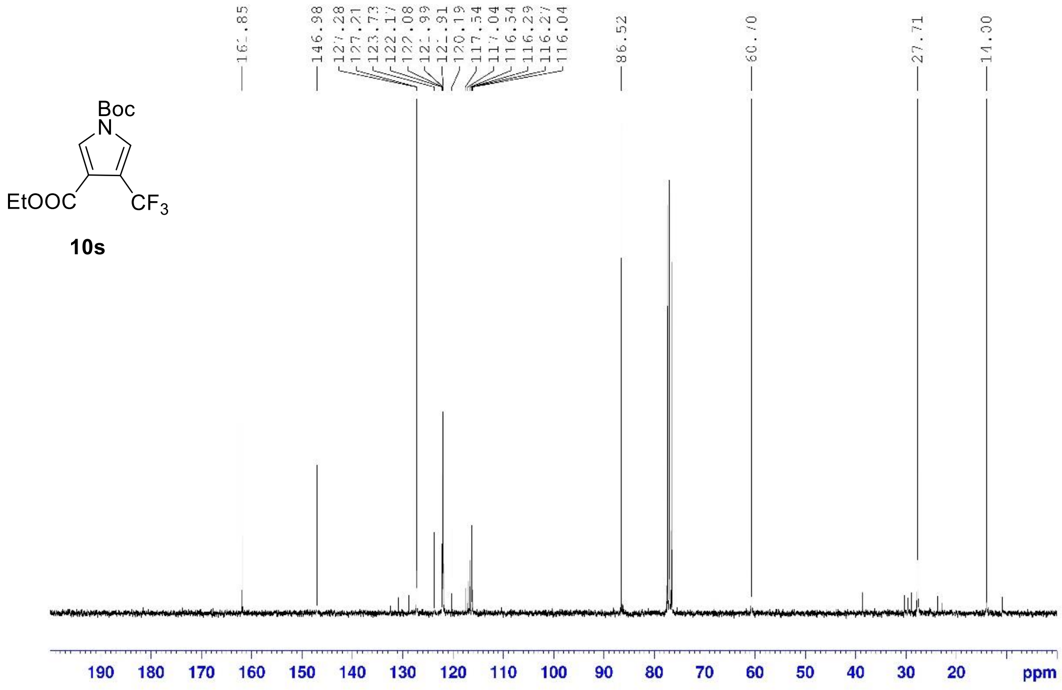

^{13}C{^{1}H } NMR (75.4 MHz, CDCl$_3$) of **10s**.

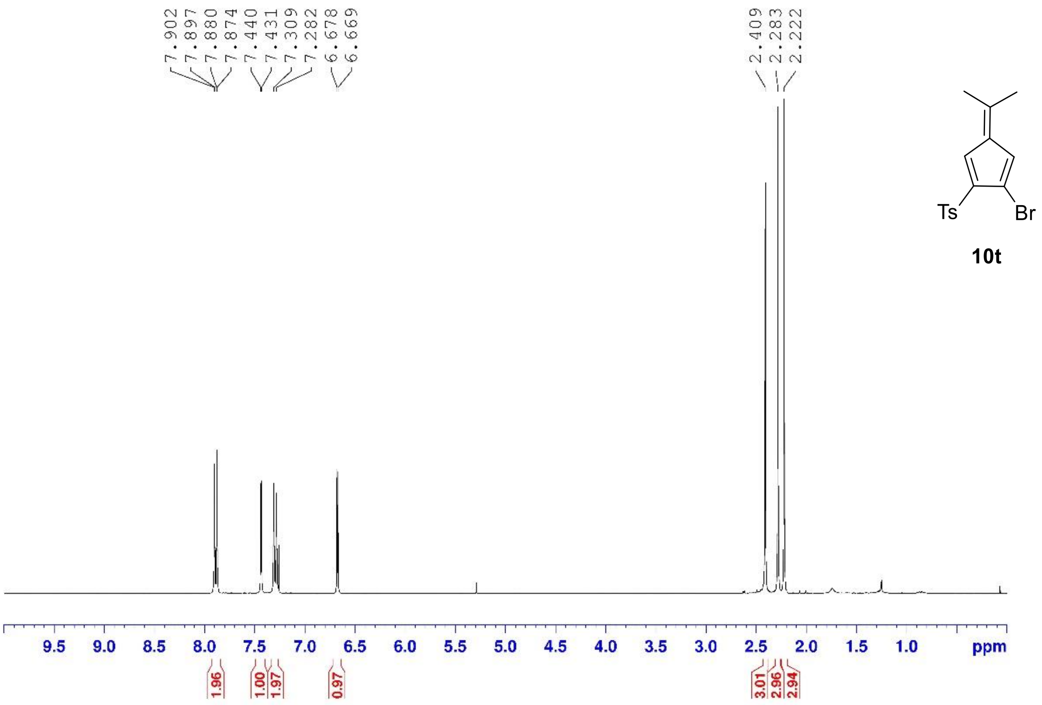

^{1}H-NMR (300 MHz, $CDCl_3$) of **10t**.

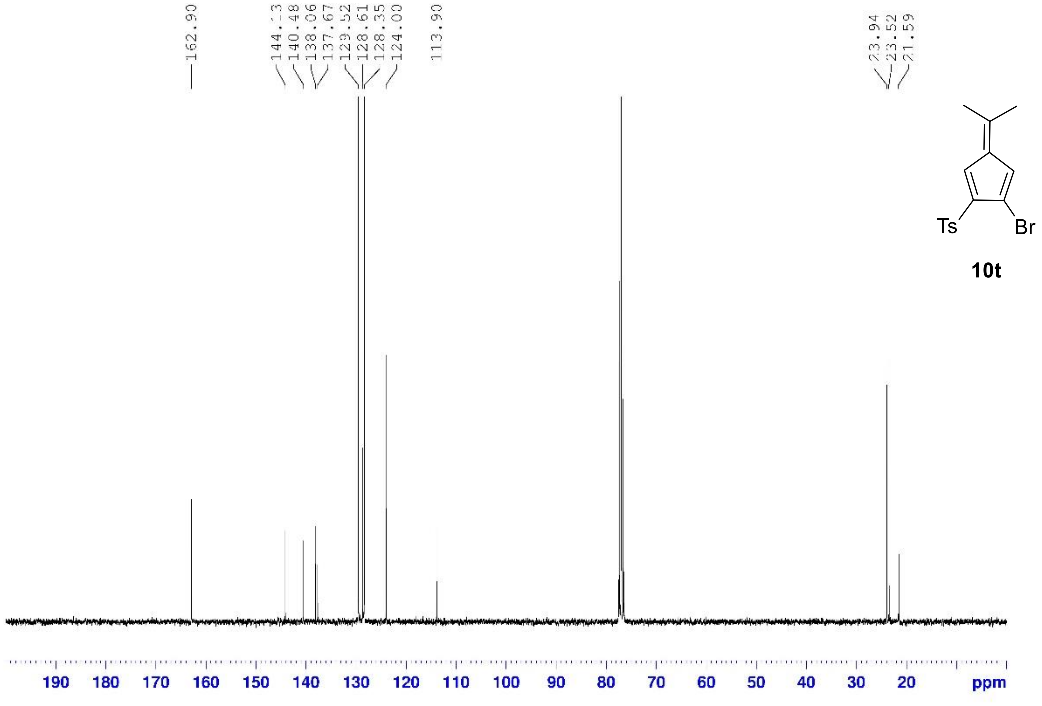

^{13}C{^{1}H } NMR (75.4 MHz, $CDCl_3$) of **10t**.

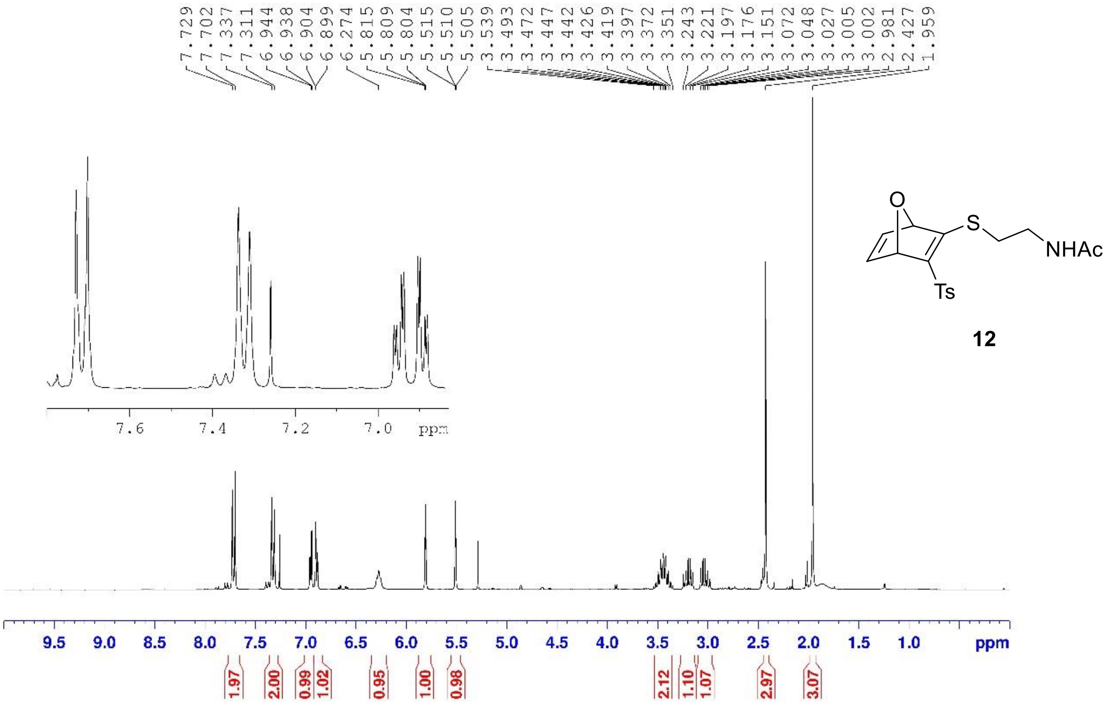

^{1}H-NMR (300 MHz, $CDCl_3$) of **12**.

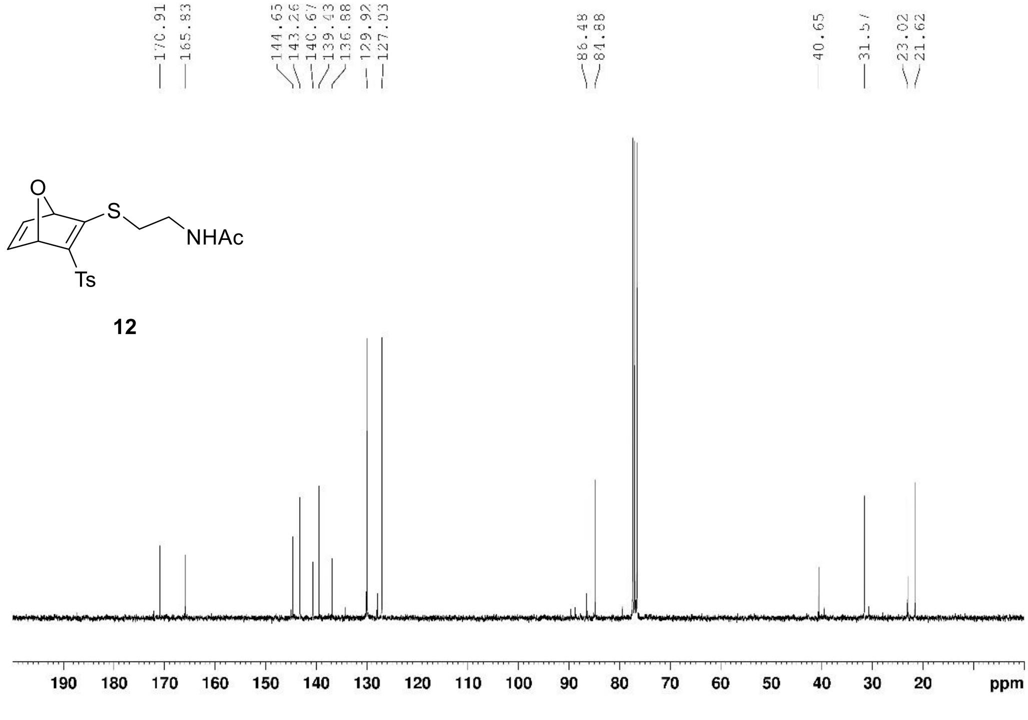

^{13}C{^{1}H } NMR (75.4 MHz, $CDCl_3$) of **12**.

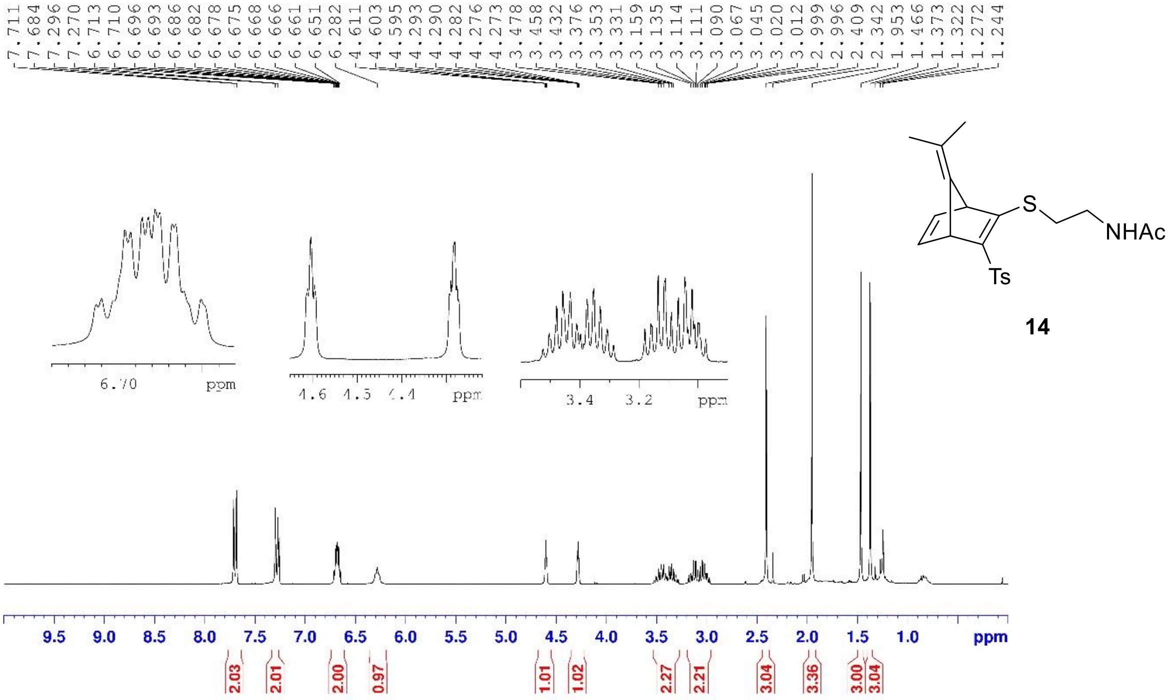

^{1}H-NMR (300 MHz, $CDCl_3$) of **14**.

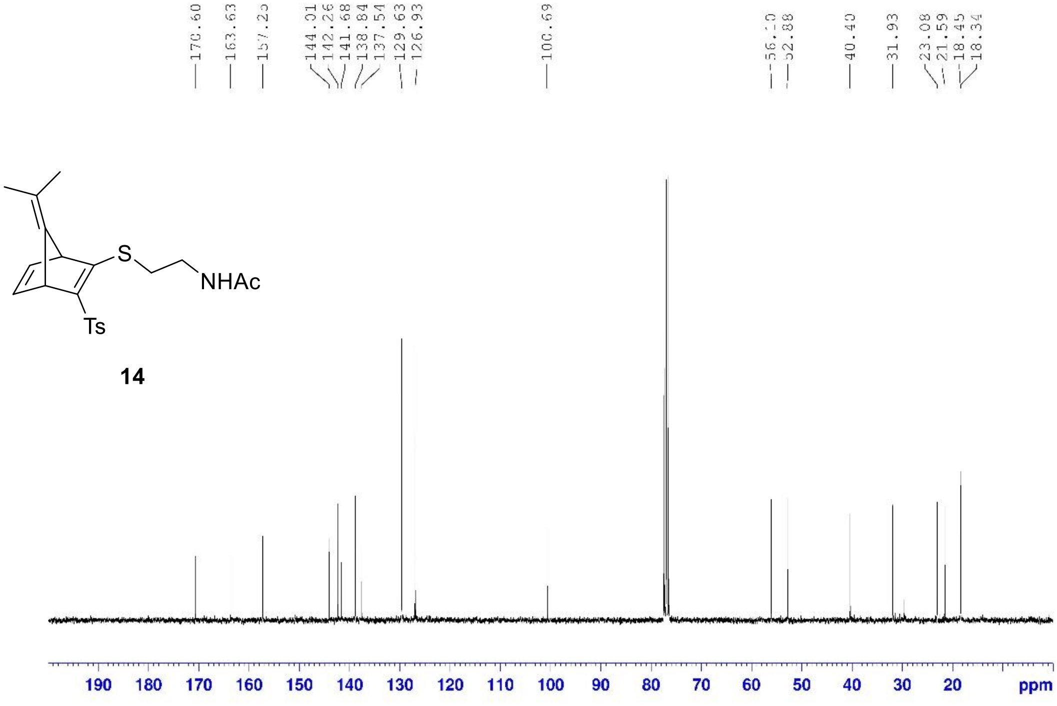

^{13}C{^{1}H } NMR (75.4 MHz, $CDCl_3$) of **14**.

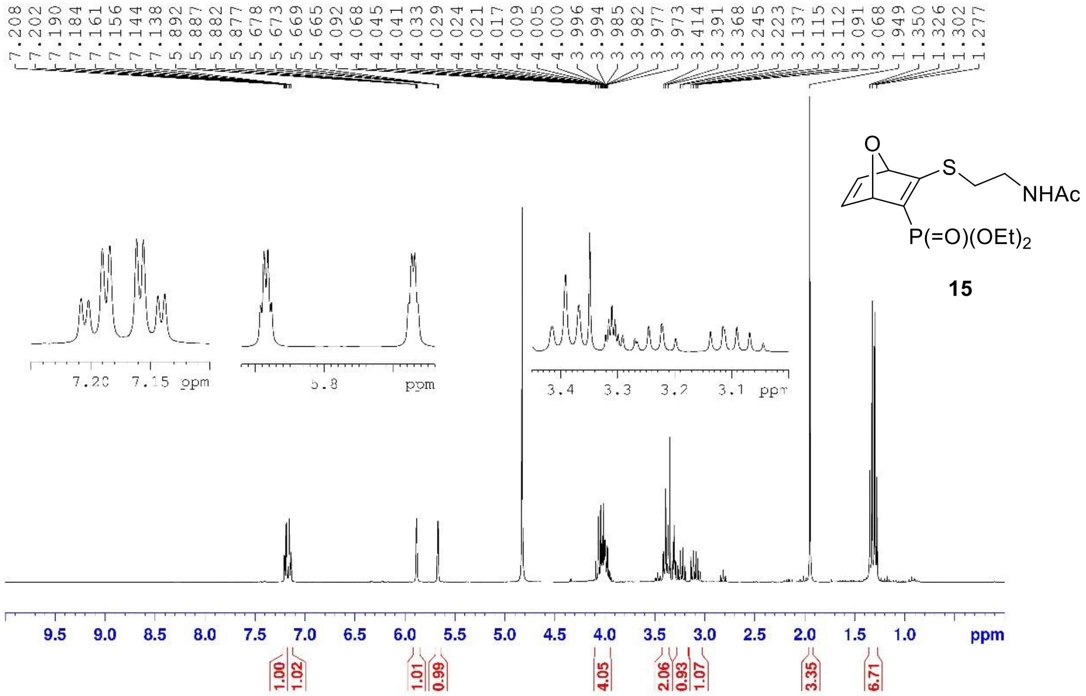

^{1}H-NMR (300 MHz, CD_3OD) of **15**.

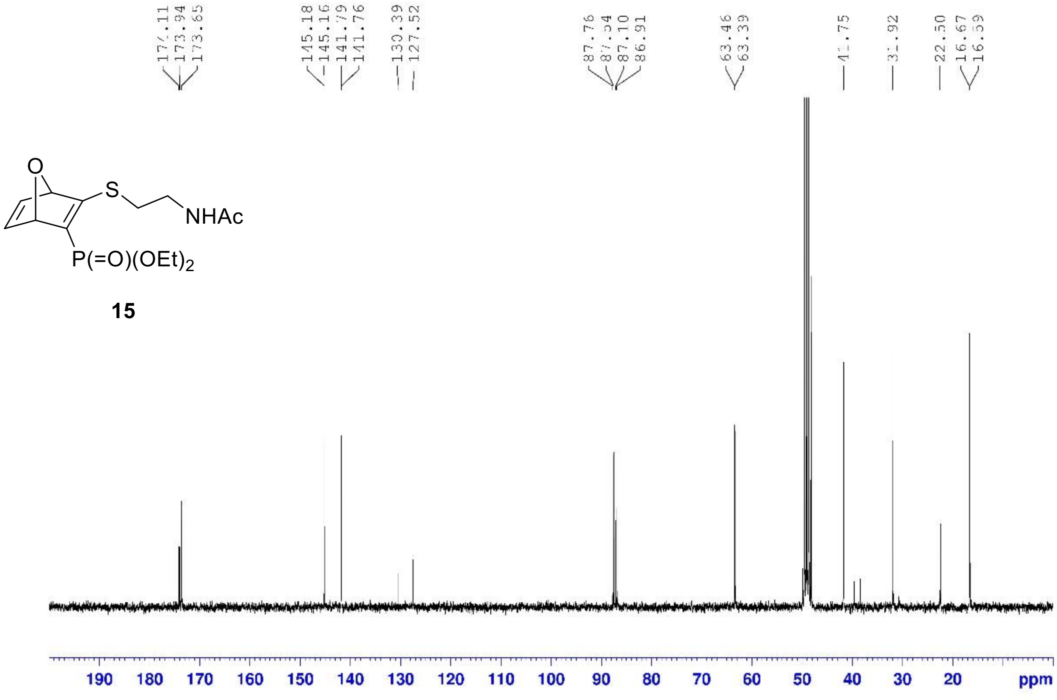

$^{13}C\{^{1}H\}$ NMR (75.4 MHz, CD_3OD) of **15**.

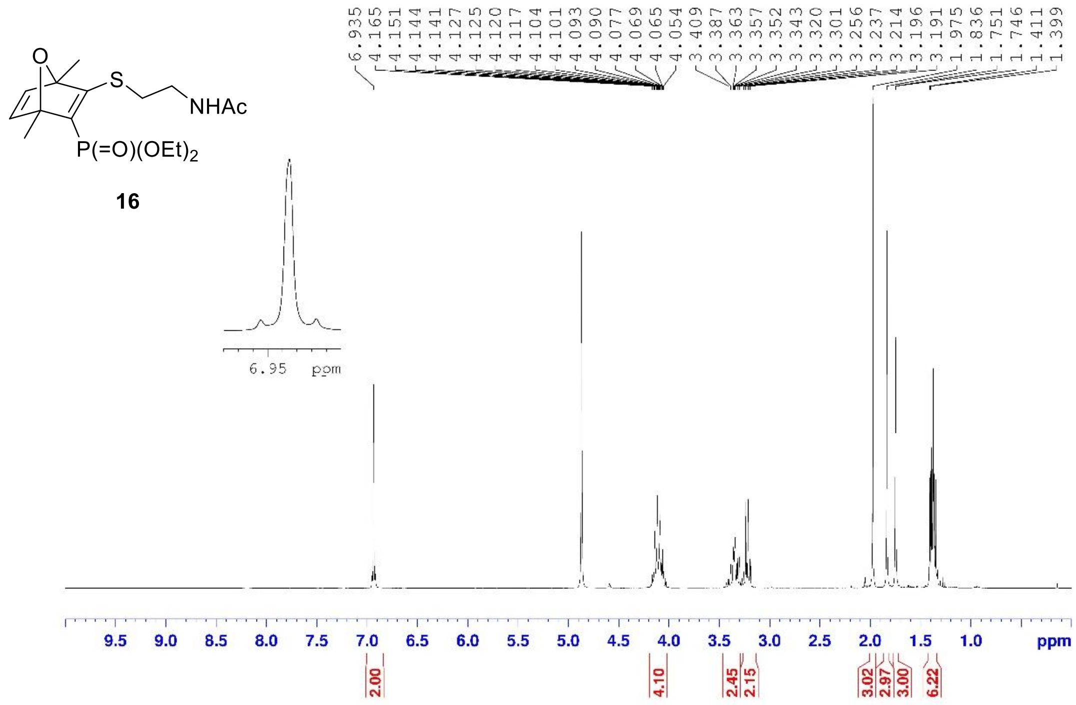

16

^{1}H-NMR (300 MHz, CD_3OD) of **16**.

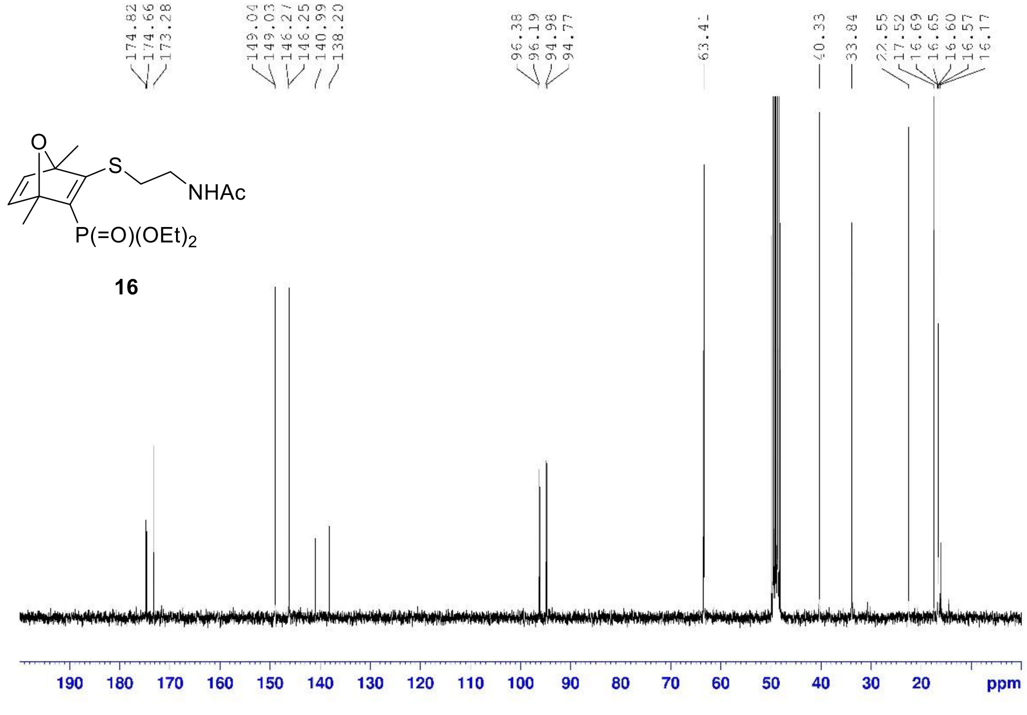

16

^{13}C{^{1}H } NMR (75.4 MHz, CD_3OD) of **16**.

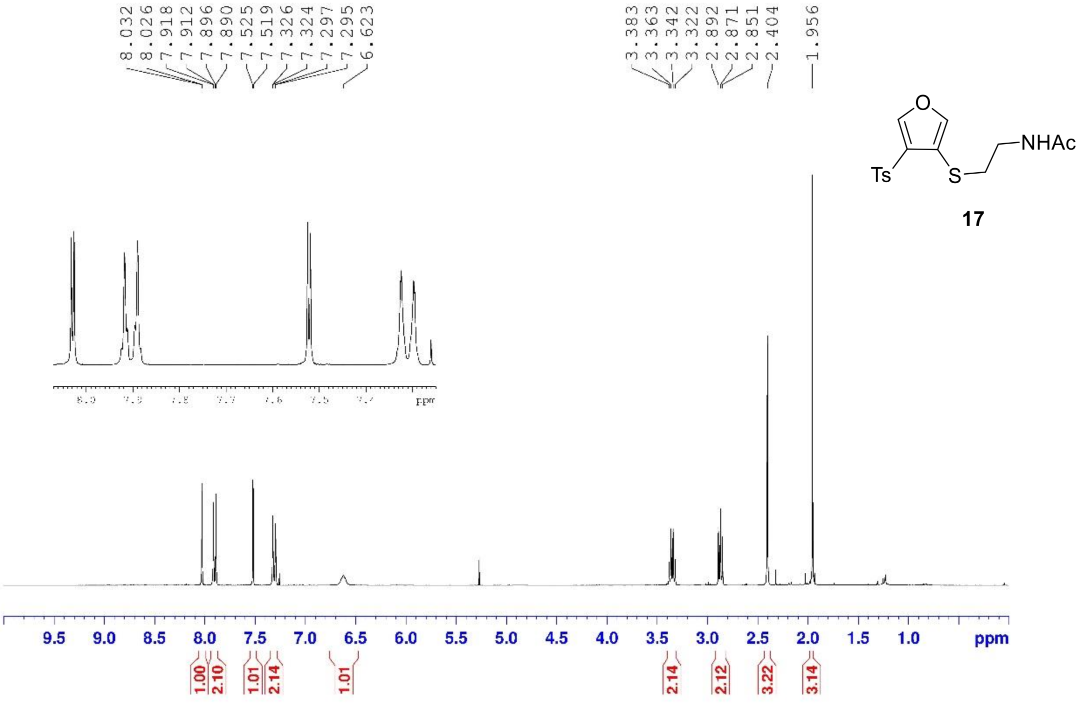

^{1}H-NMR (300 MHz, $CDCl_3$) of **17**.

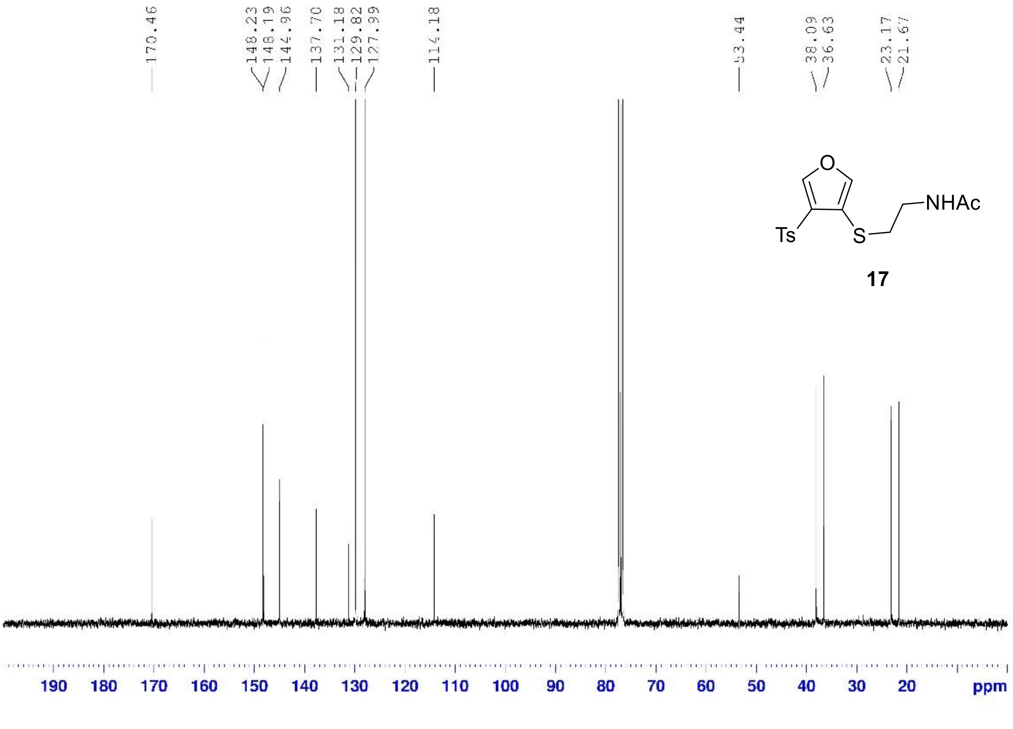

^{13}C{^{1}H } NMR (75.4 MHz, $CDCl_3$) of **17**.

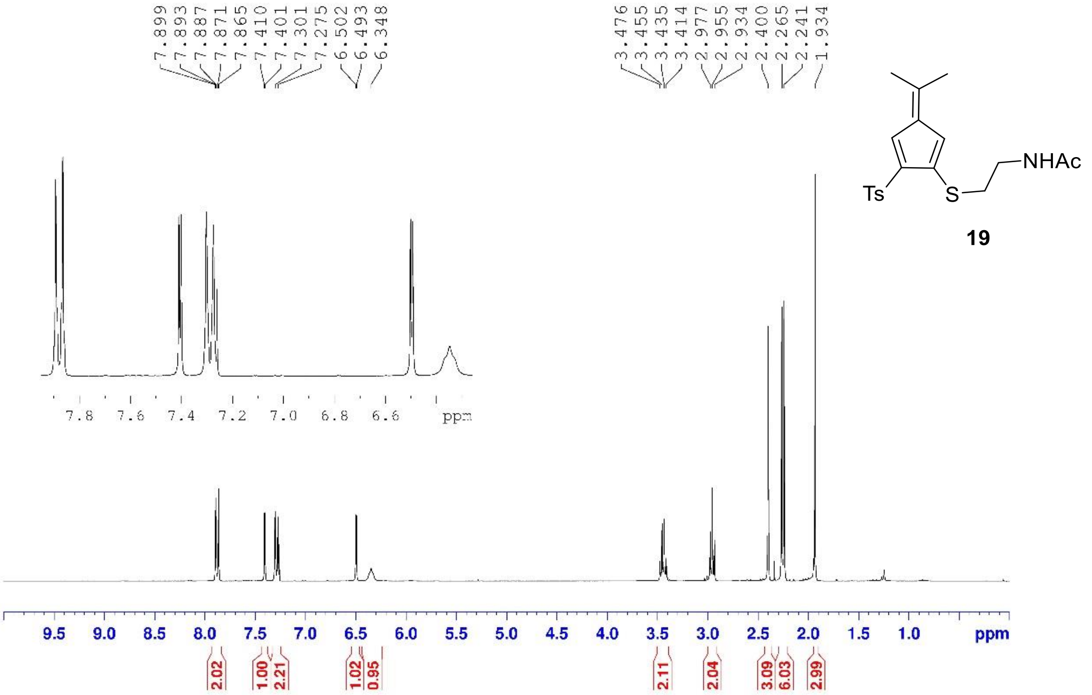

^{1}H-NMR (300 MHz, $CDCl_3$) of **19**.

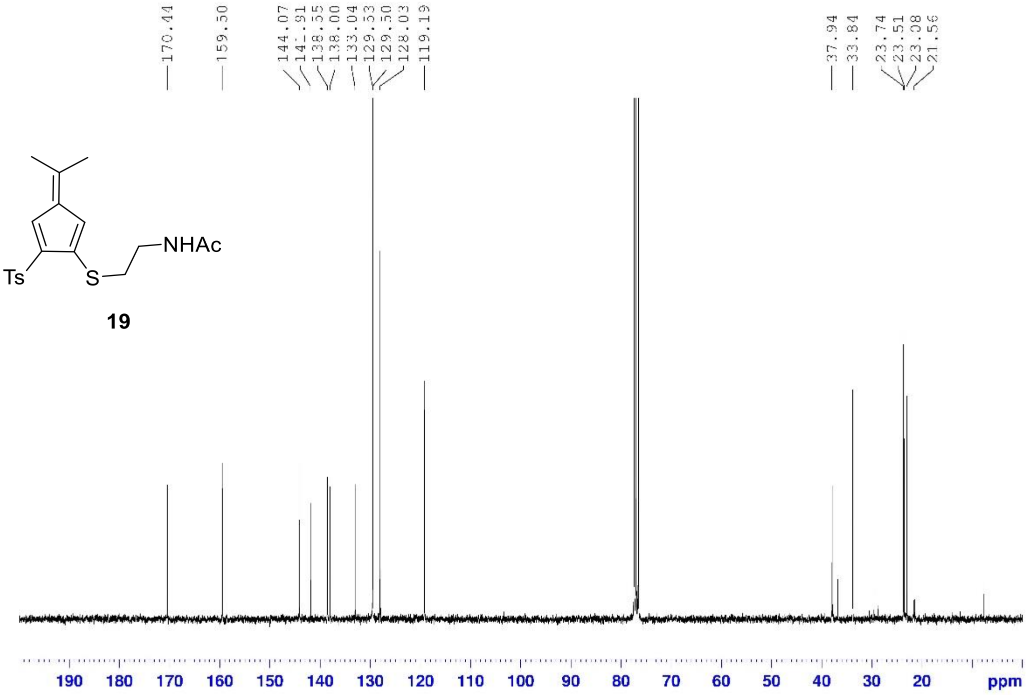

$^{13}C\{^{1}H\}$ NMR (75.4 MHz, $CDCl_3$) of **19**.

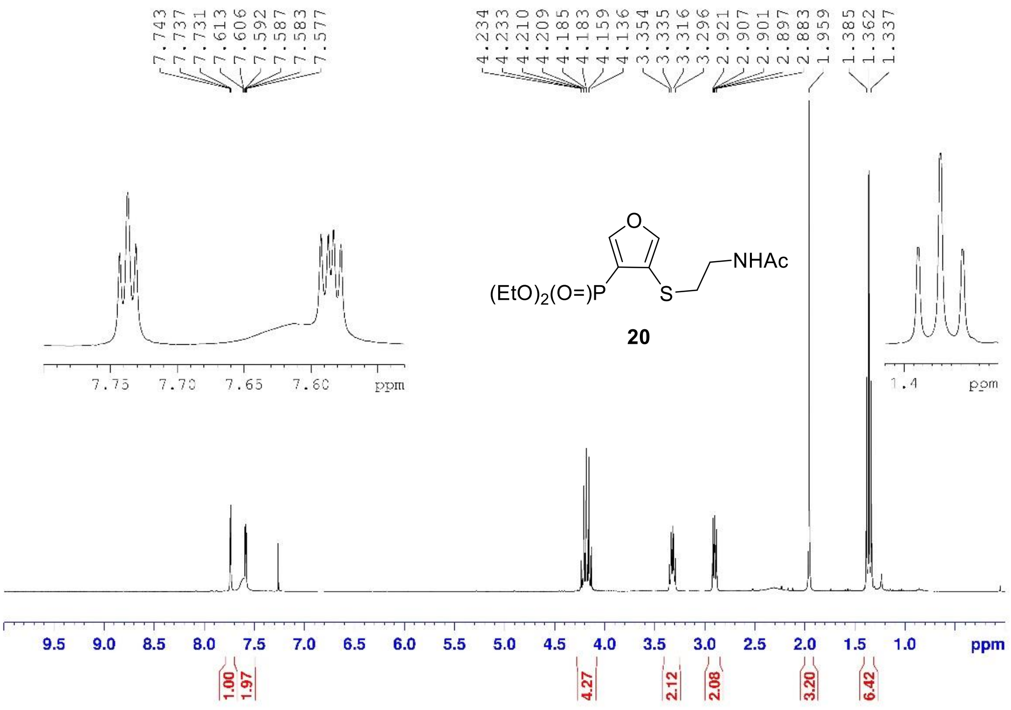

^{1}H-NMR (300 MHz, $CDCl_3$) of **20**.

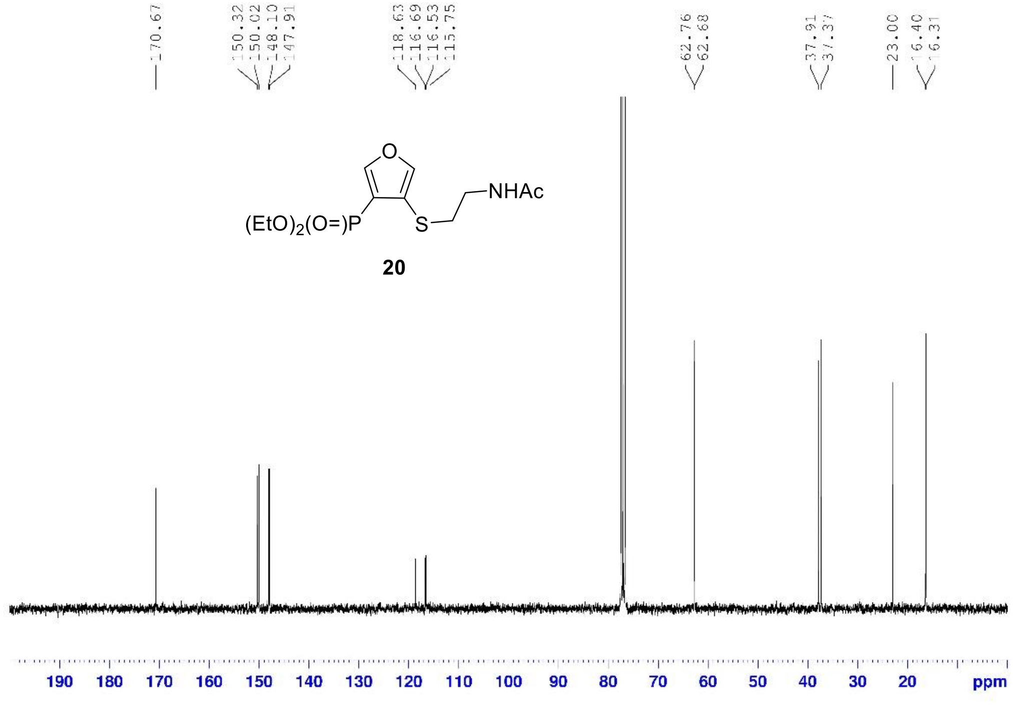

$^{13}C\{^{1}H\}$ NMR (75.4 MHz, $CDCl_3$) of **20**.

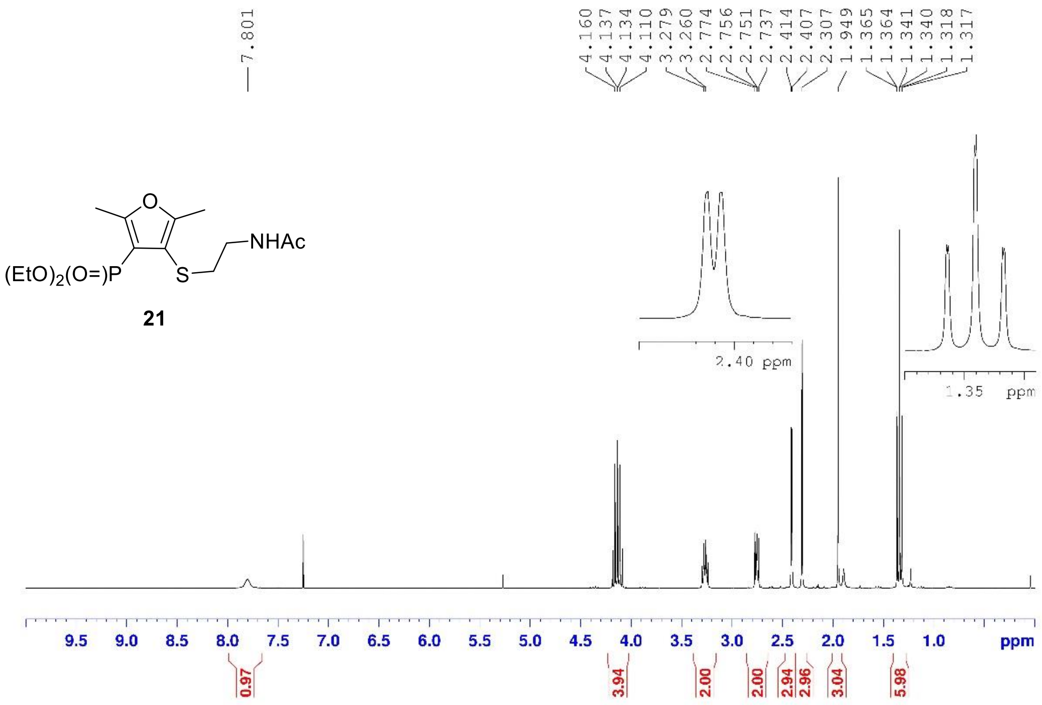

^{1}H-NMR (300 MHz, $CDCl_3$) of **21**.

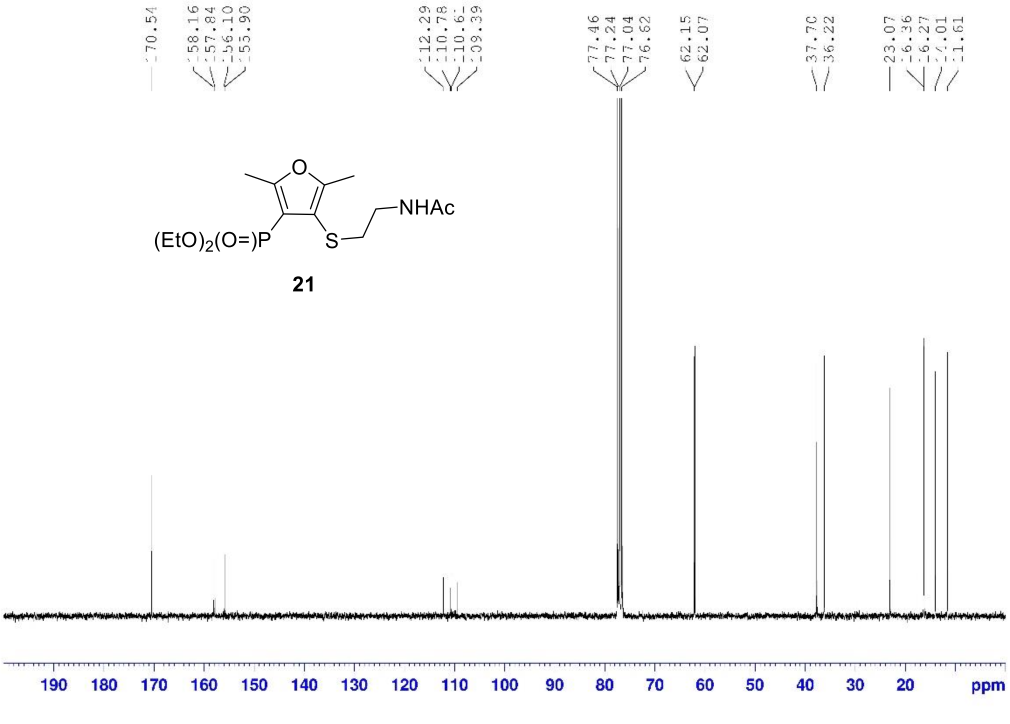

^{13}C{^{1}H } NMR (75.4 MHz, $CDCl_3$) of **21**.

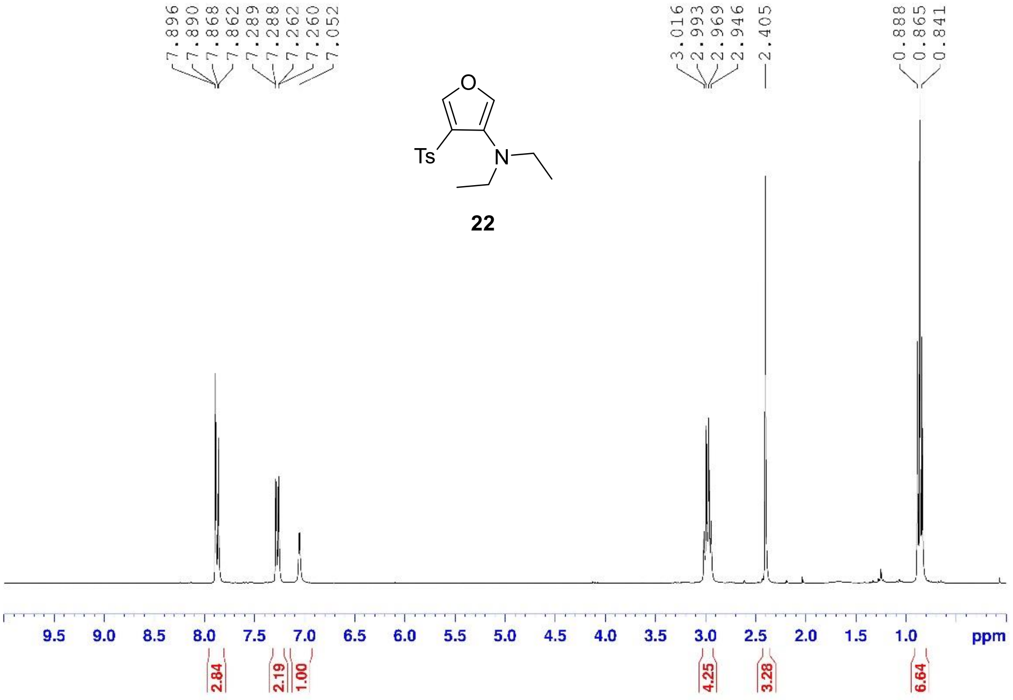

^{1}H-NMR (300 MHz, $CDCl_3$) of **22**.

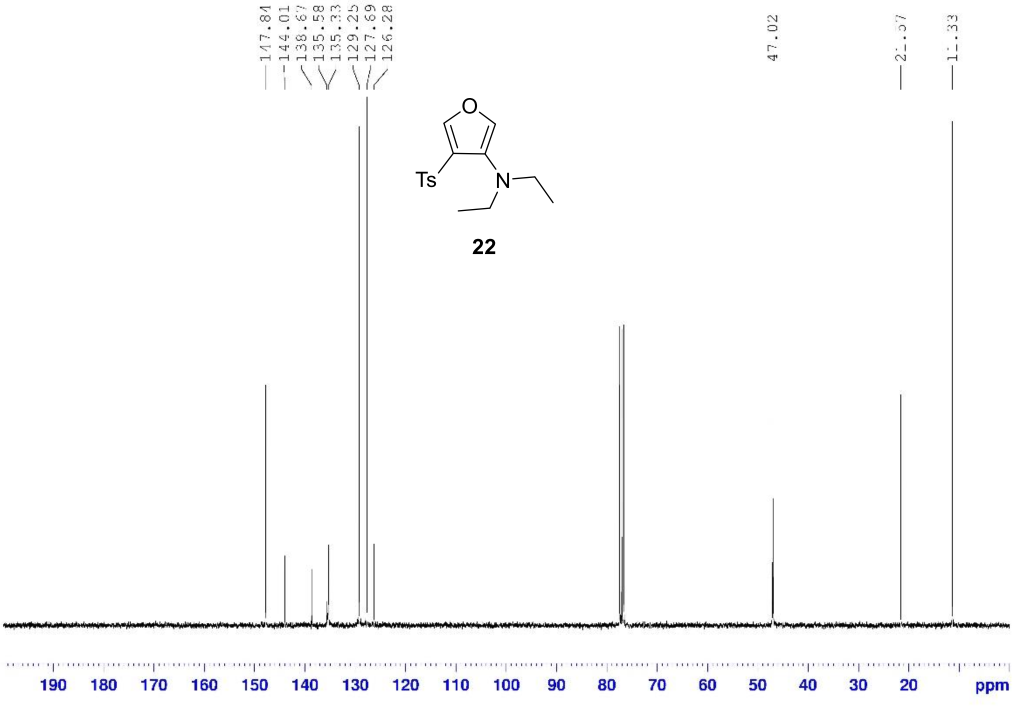

^{13}C{^{1}H } NMR (75.4 MHz, $CDCl_3$) of **22**.

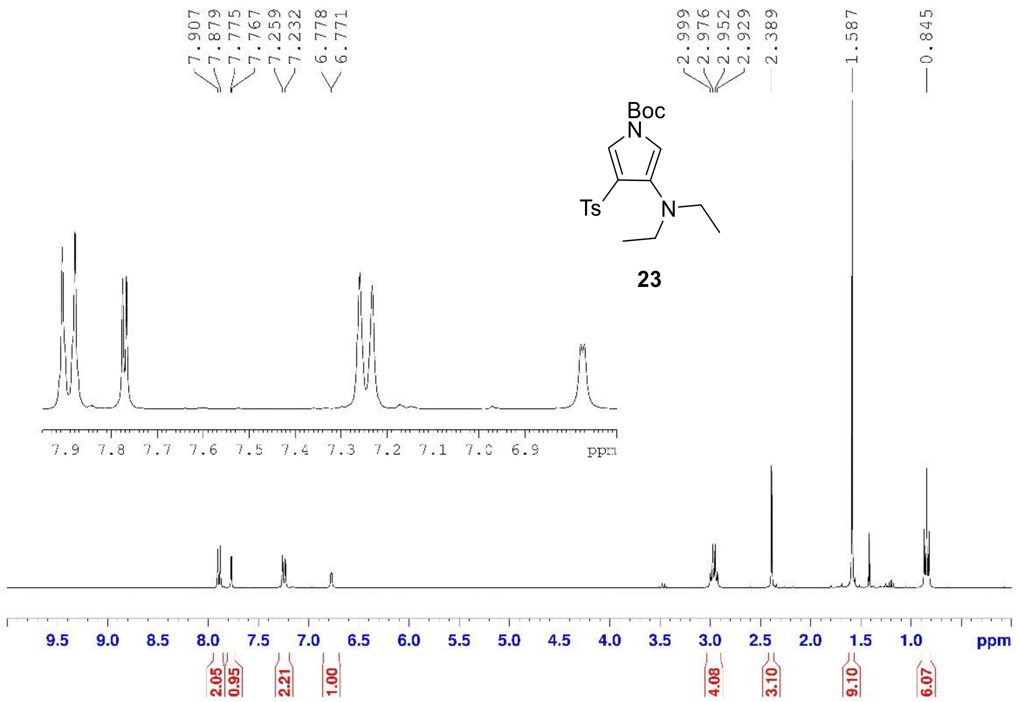

^{1}H-NMR (300 MHz, $CDCl_3$) of **23**.

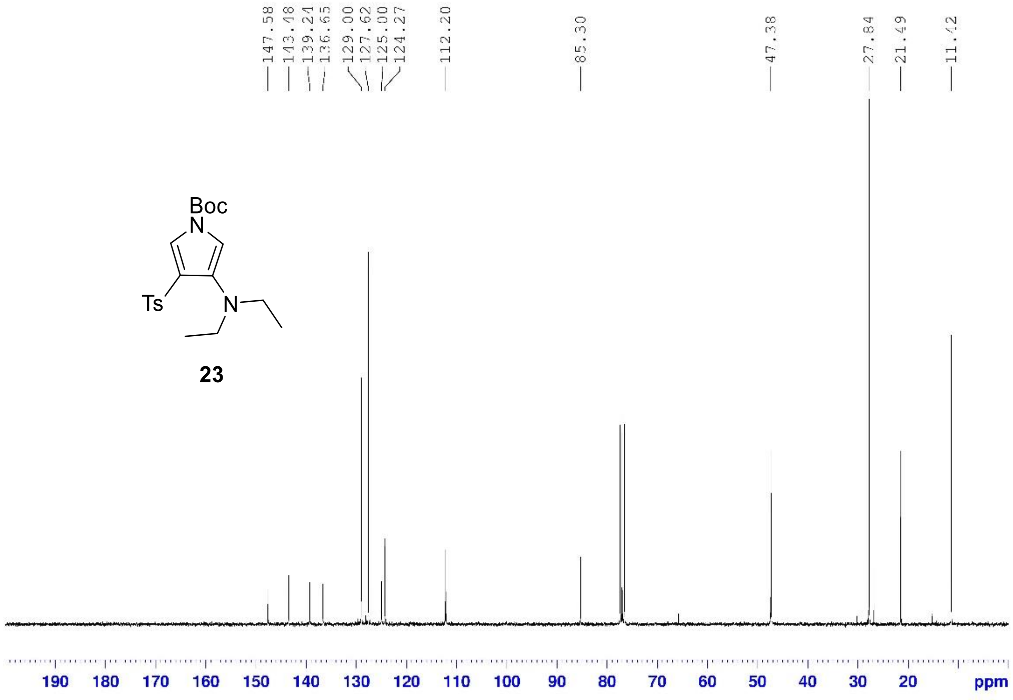

^{13}C{^{1}H } NMR (75.4 MHz, CD_3OD) of **23**.

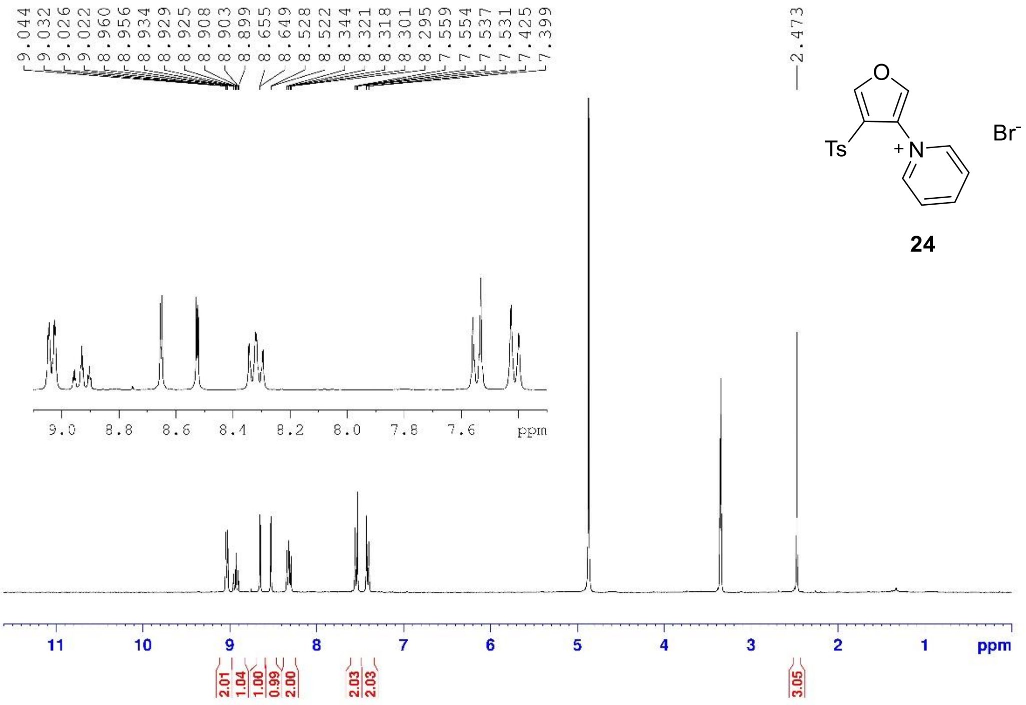

^{1}H-NMR (300 MHz, CD_3OD) of **24**.

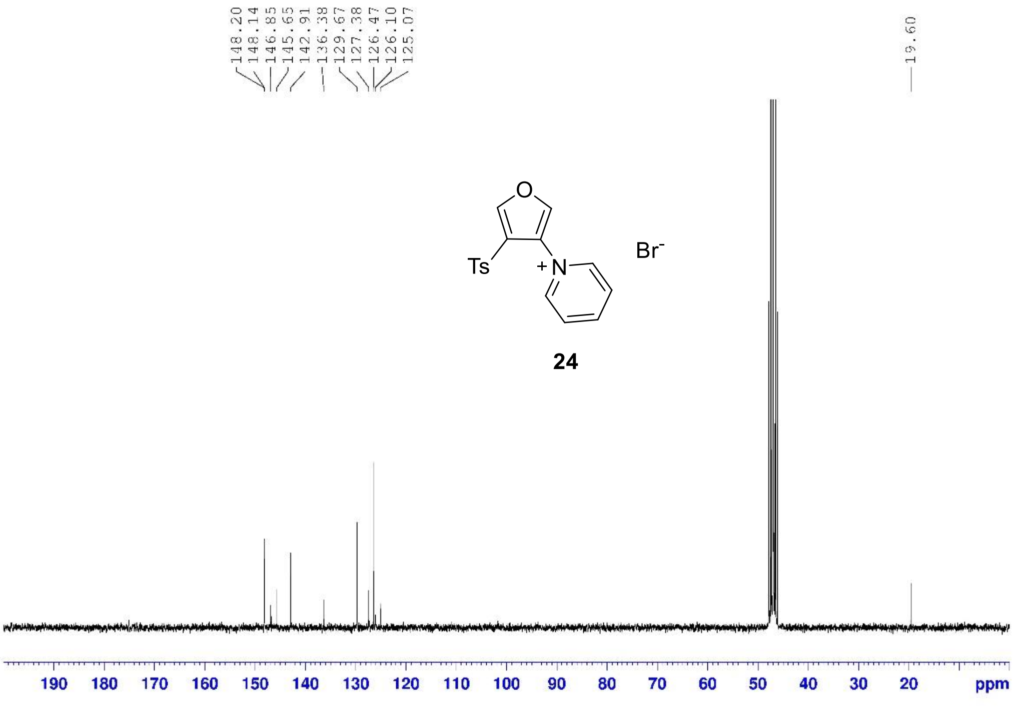

^{13}C{^{1}H } NMR (75.4 MHz, CD_3OD) of **24**.

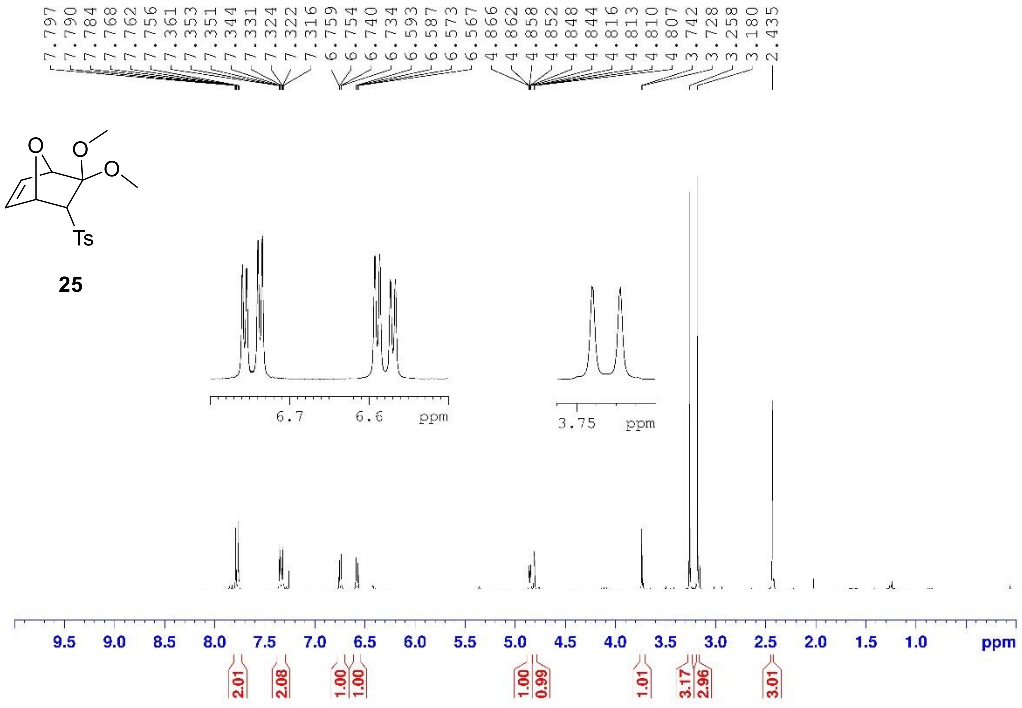

^{1}H-NMR (300 MHz, $CDCl_3$) of **25**.

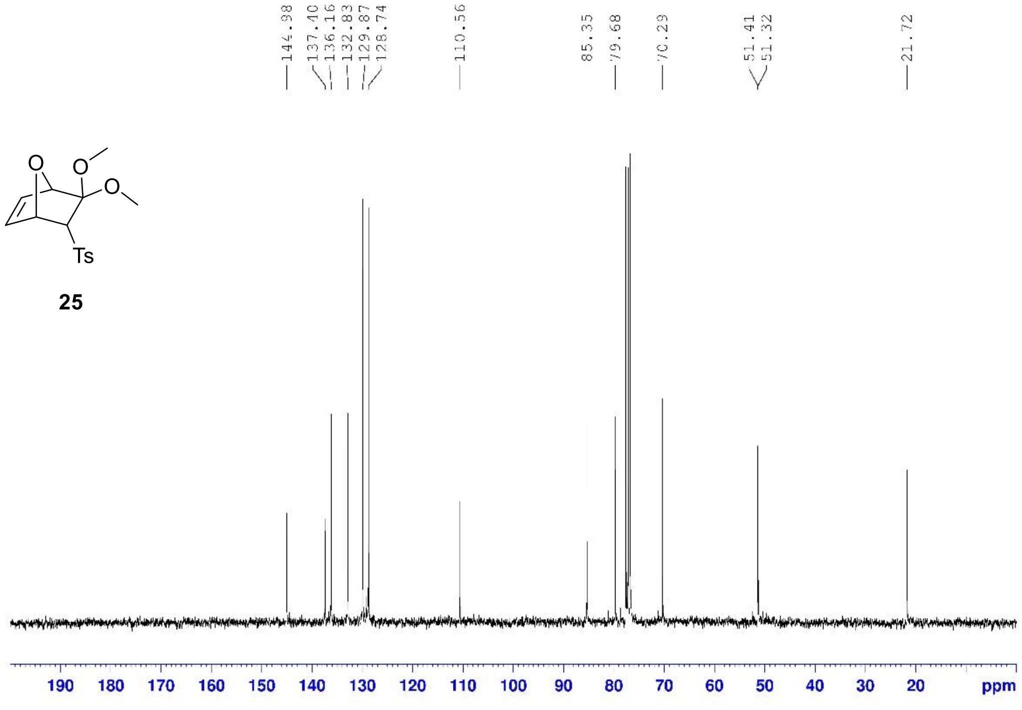

^{13}C{^{1}H } NMR (75.4 MHz, $CDCl_3$) of **25**.

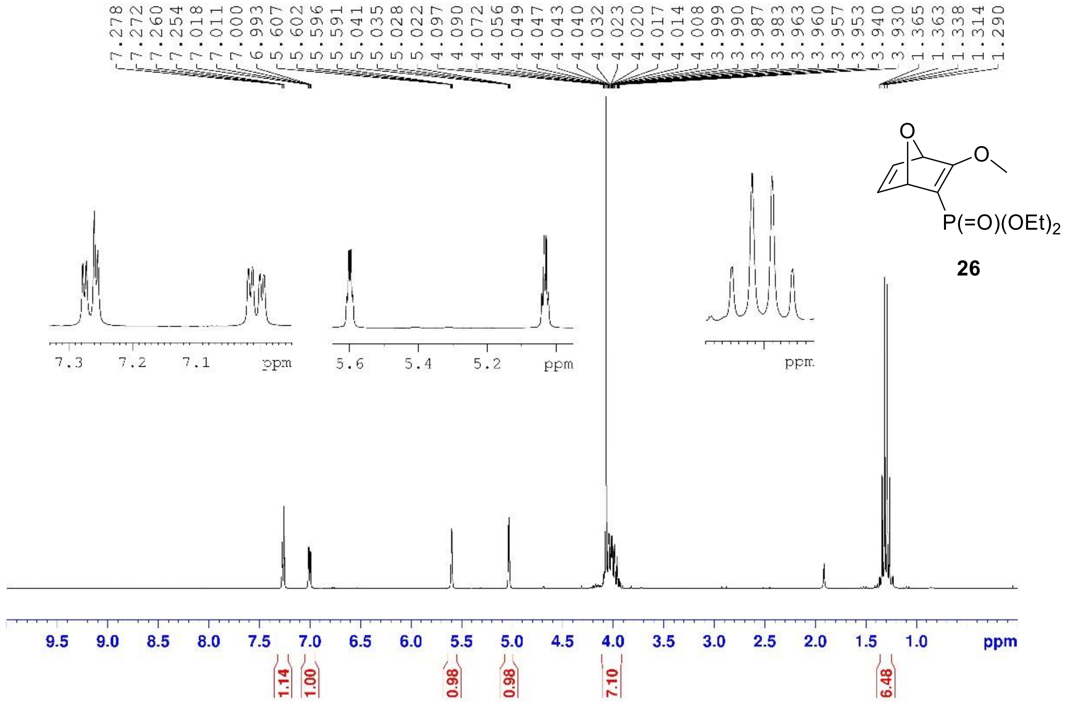

$^{13}C\{^{1}H\}$ NMR (300 MHz, $CDCl_3$) of **26**.

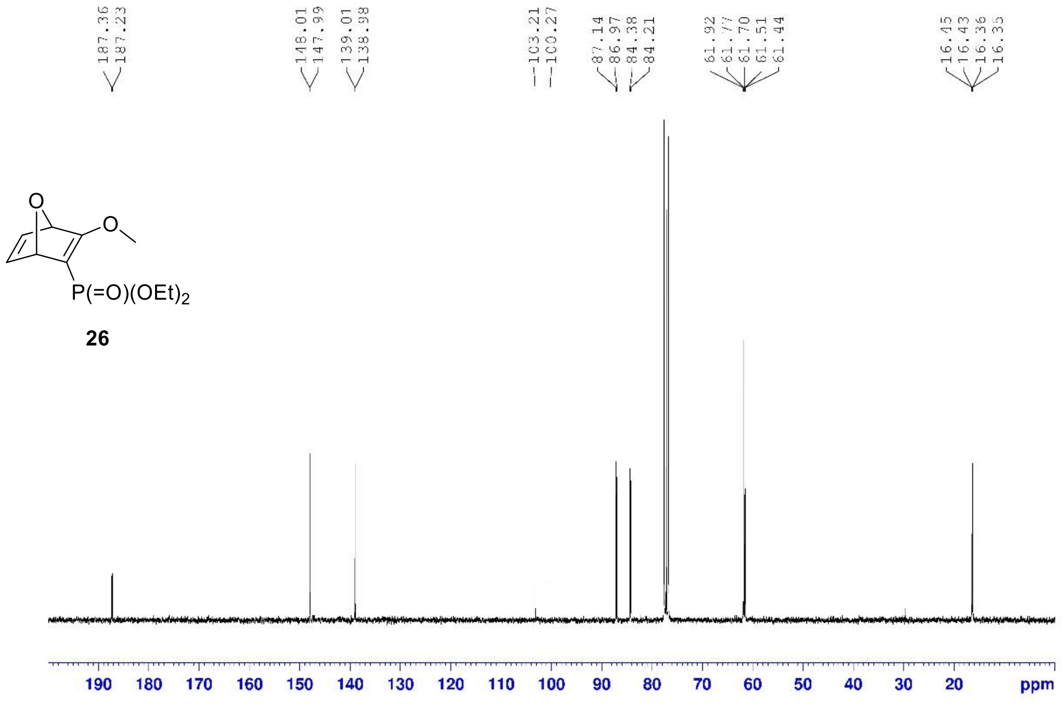

$^{13}C\{^{1}H\}$ NMR (75.4 MHz, $CDCl_3$) of **26**.

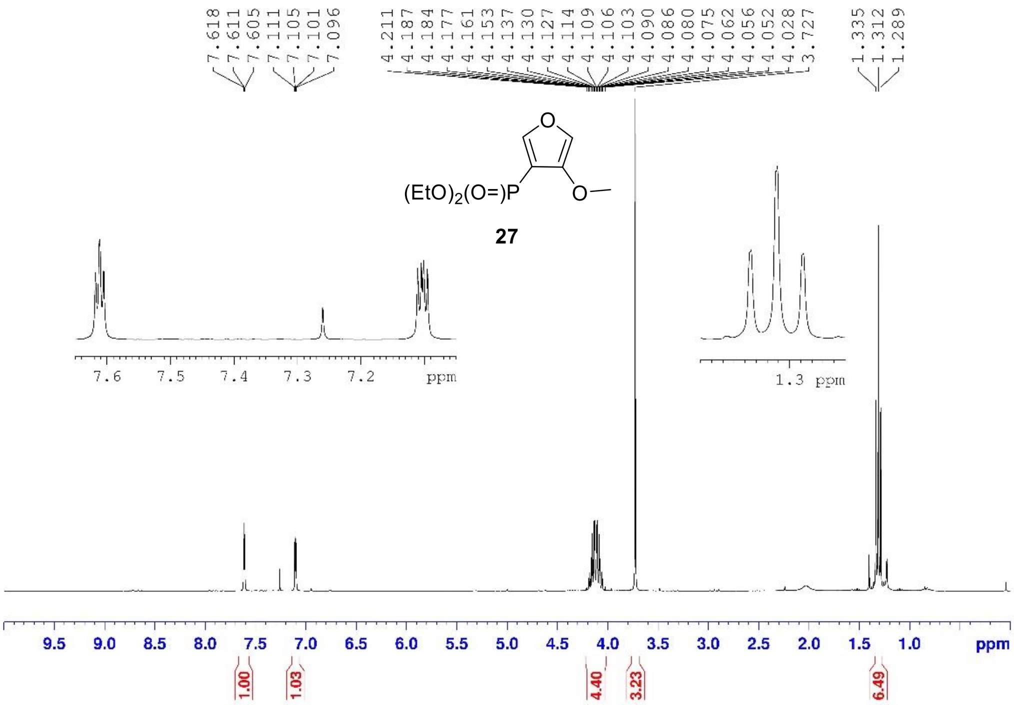

^{1}H-NMR (300 MHz, $CDCl_3$) of **27**.

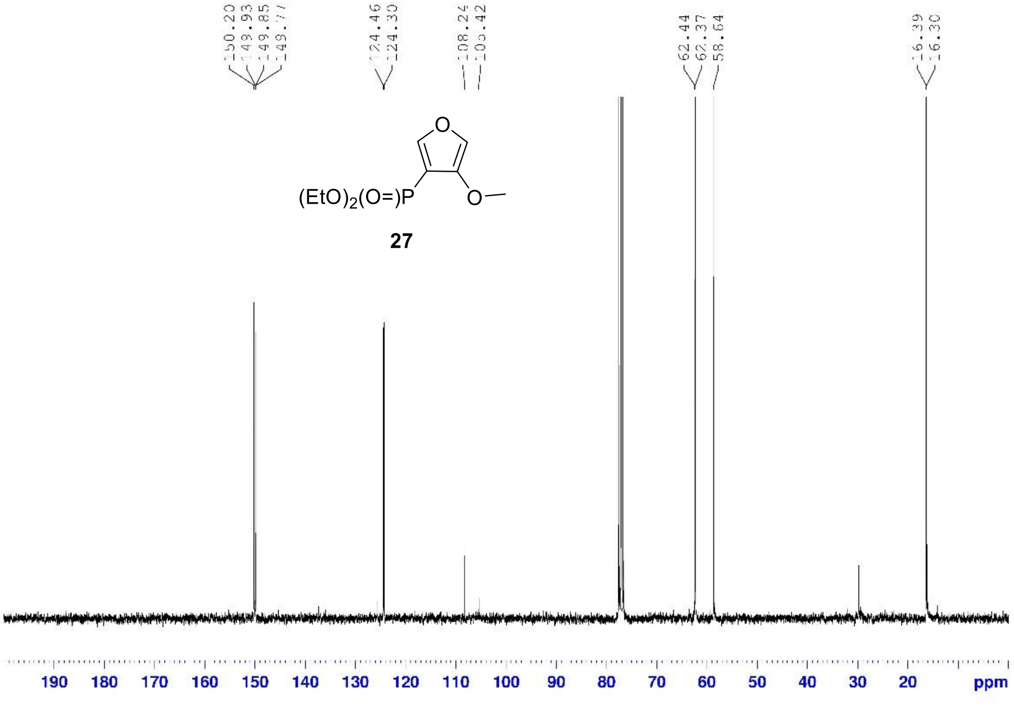

$^{13}C\{^1H\}$ NMR (75.4 MHz, $CDCl_3$) of **27**.

6. References

[1] Gil de Montes, E.; Jiménez-Moreno, E.; Oliveira, B. L.; Navo, C. D.; Cal, P. M. S. D.; Jiménez-Osés, G.; Robina, I.; Moreno-Vargas, A. J.; Bernardes, G. J. L. Azabicyclic vinyl sulfones for residue-specific dual protein labelling. *Chem. Sci.,* **2019**, *10*, 4515.

[2] Chen, Z.; Trudell, M. L. A Simplified Method for the Preparation of Ethynyl P-Tolyl Sulfone and Ethynyl Phenyl Sulfone. *Synth. Commun*., **1994**, *24*, 3149.

[3] Zhang, C.; Ballay II, C. J.; Trudell, M. L. 2-Bromoethynyl Aryl Sulfones as Versatile Dienophiles: a Formal Synthesis of Epibatidine. *J. Chem. Soc., Perkin Trans. 1*, **1999**, 675.

[4] Poulsen, T. B.; Bernardi, L.; Alemán, J.; Overgaard, J.; Jørgensen, K. A. Organocatalytic Asymmetric Direct α-Alkynylation of Cyclic β-Ketoesters. *J. Am. Chem. Soc.* **2007**, *129*, 441.

[5] Leroy, J. *Synth. Commun.* **1992**, *22*, 567

[6] Gil de Montes, E.; Martínez-Bailén, M.; Carmona, A. T.; Robina, I.; Moreno-Vargas, A. J. Regioselectivity of the 1,3-Dipolar Cycloaddition of Organic Azides to 7-Heteronorbornadienes. Synthesis of β-Substituted Furans/Pyrroles. *J. Org. Chem.*, **2020**, *85*, 8923.

[7] (a) Wanat, P.; Walczak, S.; Wojtczak, B. A.; Nowakowska, M.; Jemielity, J.; Kowalska, J. Ethynyl, 2-Propynyl, and 3-Butynyl C-Phosphonate Analogues of Nucleoside Di- and Triphosphates: Synthesis and Reactivity in CuAAC. Org. Lett., 2015, 17, 3062). (b) Oakdale, J. S.; Sit, R. K.; Fokin, V. V. Ruthenium-Catalyzed Cycloadditions of 1-Haloalkynes with Nitrile Oxides and Organic Azides: Synthesis of 4-Haloisoxazoles and 5-Halotriazoles. *Chem. Eur. J.* **2014**, *20*, 11101.

[8] Ouairy, C.; Michel, P.; Delpech, B.; Crich, D.; Marazano, C. Synthesis of N-Acyl-5-aminopenta-2,4-dienals via Base-Induced Ring-Opening of N-Acylated Furfurylamines: Scope and Limitations. *J. Org. Chem*. **2010**, *75*, 4311.

[9] (a) For the synthesis of **9a**, see: Plumet, J.; Rincon, R.; Aljarilla, A.; Criado, M. Straightforward Synthesis of Strained α,β-Epoxysulfones via Epoxidation of Vinylsulfones Using *N*-Methylmorpholine *N*-Oxide as Epoxidizing Reagent. *Synlett* **2007**, 1948−1950. (b) For the synthesis of **9b** and **9o**, see: Zhang, C.; Ballay II, C. J.; Trudell, M. L. 2-Bromoethynyl Aryl Sulfones as Versatile Dienophiles: a Formal Synthesis of Epibatidine. *J. Chem. Soc., Perkin Trans. 1* **1999**, 675−676. (c) For the synthesis of **9e** and **9f**, see: van Berkel, S. S.; Dirks, A. J.; Debets, M. F.; van Delft, F. L.; Cornelissen, J. J. L. M.; Nolte, R. J. M.; Rutjes, F. P. J. T. Metal-free triazole formation as a tool for bioconjugation. *ChemBioChem* **2007**, 8, 1504-1508. (d) For the synthesis of **9g**, see: Rainier, J. D.; Xu, Q. A Novel Anionic Condensation, Fragmentation, and Elimination Reaction of Bicyclo[2.2.1]heptenone Ring Systems. *Org. Lett.* **1999**, *1*, 27−30. (e) For the synthesis of **9i**, see: Kislukhin, A. A.; Higginson, C. J.; Finn, M. G. Aqueous-phase deactivation and intramolecular [2+2+2] cycloaddition of oxanorbornadiene esters. *Org. Lett*. **2011**, *13*, 1832-1835. (f) For the synthesis of **9n**, see: Leung-Toung, R.; Liu, Y.; Muchowski, J. M.; Wu, Y.-L. Synthesis of Conduramines from N-tert-Butoxycarbonylpyrrole. *J. Org. Chem*. **1998**, *63*, 3235−3250. (g) For the synthesis of **9p**, see reference 6. (h) For the synthesis of **9r**, see: Weeresakare, G. M.; Xu, Q.; Rainier, J. D. An Anionic Condensation and Fragmentation Approach to Substituted 3-Pyrrolines. *Tetrahedron Lett*. **2002**, *43*, 8913−8915. (i) For the synthesis of **9s**, see: Troelsen, N. S.; Shanina, E.; Gonzalez-Romero, D.; Danková, D.; Jensen, I. S. A.; Śniady, K. J.; Nami, F.; Zhang, H.; Rademacher, C.; Cuenda, A.; Gotfredsen, C. H.; Clausen, M. H. The 3F Library: Fluorinated Fsp^3-Rich Fragments for Expeditious 19F-NMR Based Screening. *Angew. Chem. Int. Ed*. **2020**, *59*, 2204 –2210.

[10] Moreno-Clavijo, E.; Moreno-Vargas, A. J.; Carmona, A. T.; Robina, I. *Org. Biomol. Chem*., **2013**, *11*, 7016-7025.
